# The WNT/β‐catenin dependent transcription: A tissue‐specific business

**DOI:** 10.1002/wsbm.1511

**Published:** 2020-10-21

**Authors:** Simon Söderholm, Claudio Cantù

**Affiliations:** ^1^ Wallenberg Centre for Molecular Medicine Linköping University Linköping Sweden; ^2^ Department of Biomedical and Clinical Sciences, Division of Molecular Medicine and Virology, Faculty of Health Science Linköping University Linköping Sweden

**Keywords:** cell signaling, genomics, transcription, Wnt signaling, β‐catenin

## Abstract

β‐catenin‐mediated Wnt signaling is an ancient cell‐communication pathway in which β‐catenin drives the expression of certain genes as a consequence of the trigger given by extracellular WNT molecules. The events occurring from signal to transcription are evolutionarily conserved, and their final output orchestrates countless processes during embryonic development and tissue homeostasis. Importantly, a dysfunctional Wnt/β‐catenin pathway causes developmental malformations, and its aberrant activation is the root of several types of cancer. A rich literature describes the multitude of nuclear players that cooperate with β‐catenin to generate a transcriptional program. However, a unified theory of how β‐catenin drives target gene expression is still missing. We will discuss two types of β‐catenin interactors: transcription factors that allow β‐catenin to localize at target regions on the DNA, and transcriptional co‐factors that ultimately activate gene expression. In contrast to the presumed universality of β‐catenin's action, the ensemble of available evidence suggests a view in which β‐catenin drives a complex system of responses in different cells and tissues. A malleable armamentarium of players might interact with β‐catenin in order to activate the right “canonical” targets in each tissue, developmental stage, or disease context. Discovering the mechanism by which each tissue‐specific β‐catenin response is executed will be crucial to comprehend how a seemingly universal pathway fosters a wide spectrum of processes during development and homeostasis. Perhaps more importantly, this could ultimately inform us about which are the tumor‐specific components that need to be targeted to dampen the activity of oncogenic β‐catenin.

This article is categorized under:

Cancer > Molecular and Cellular Physiology
Cancer > Genetics/Genomics/Epigenetics
Cancer > Stem Cells and Development

## Graphical abstract

The Wnt/β‐catenin dependent transcription is executed via a complex network of dynamically interacting co‐factors whose reciprocal requirements and timely modes of action we do not fully understand.

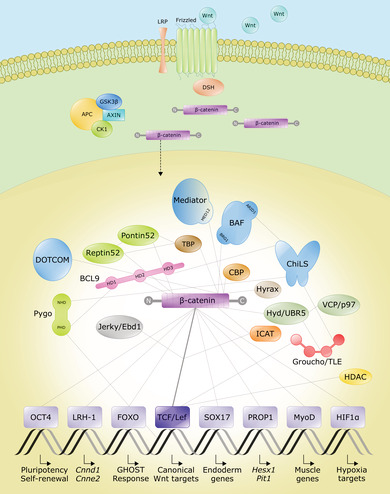

## 1 INTRODUCTION

Wnt signaling refers to a series of cell‐to‐cell communication pathways that take place when a cell receives a WNT signal molecule produced by another cell and responds by triggering well‐defined intracellular biochemical events. The mammalian genome harbors 19 *WNT* genes which possess both distinct as well as overlapping functions during animal development (Du, Purcell, Christian, McGrew, & Moon, 1995). The cascades that the WNTs trigger are classically separated into three types: canonical Wnt signaling, where β‐catenin transforms the signal into a gene‐expression program; the Wnt/PCP (Planar Cell Polarity), that organizes cells in the plane of the tissue; and the protein kinase C (PKC)‐dependent Wnt pathway, that causes increased intracellular Ca^2+^ levels and the activation of downstream effectors. Collectively, these pathways are evolutionarily conserved in the animal kingdom, and they control cell behavior, stem cell pool maintenance, regeneration, differentiation, and many other cellular processes. For the reader interested in the differences between these mechanisms we refer to a number of authoritative reviews written in the course of the last two decades (Cadigan & Nusse, 1997; Nusse & Clevers, 2017; Tortelote, Reis, de Almeida Mendes, & Abreu, 2017). Here we will focus on the β‐catenin‐mediated Wnt pathway.

β‐catenin is a peculiar bi‐functional protein that normally resides at the cytoplasmic side of the *adherence junctions* in epithelial cells, in contact with the cadherins complex (Dickinson, Nelson, & Weis, 2011; Peifer, Rauskolb, Williams, Riggleman, & Wieschaus, 1991). A small pool of E‐cadherin‐free β‐catenin is also present in the cytoplasm; however, cytosolic β‐catenin is rapidly marked for proteasome‐mediated degradation. In the Wnt/β‐catenin pathway, the primary effect triggered by the extracellular WNT proteins is the stabilization of cytosolic β‐catenin (Peifer et al., 1991). Thus, β‐catenin is “saved” from degradation, accumulates, and travels to the nucleus where, in concert with several other players, activates a highly specific gene‐expression program. Wnt/β‐catenin signaling is fundamental for virtually all developmental processes, from gastrulation (Kelly, Pinson, & Skarnes, 2004), axes specification (Hikasa & Sokol, 2013), organogenesis, tissue regeneration (Tanaka & Reddien, 2011; Whyte, Smith, & Helms, 2012), and adult stem cells maintenance (Sato et al., 2011).

β‐catenin's mode of action is thought to be universally conserved across species and cell types to the point of earning the status of “canonical” Wnt signaling. However, consistently with its role in various processes, Wnt/β‐catenin signaling activates different sets of genes in different contexts (Cantù et al., 2018; Sinner, Rankin, Lee, & Zorn, 2004). Even individual genes that are historically consolidated as targets of this cascade, such as *Ccnd1* (encoding for Cyclin D1), can be regulated in a context dependent manner rather than being ubiquitous targets (Sansom et al., 2005). The ample spectrum of roles and responses that Wnt/β‐catenin signaling sustains has been often attributed to the number of possible permutations between the “use” of different WNT ligands and the available receptors and co‐receptors on the cell. These permutations might certainly confer variability in the subsequent activation of the downstream events (Nusse & Clevers, 2017). However, we find it difficult to hypothesize that different ligand/receptor combinations could lead to divergent outputs if their signals have to be transduced anyway by a universal transcriptional complex. The presumed universality of nuclear β‐catenin's mechanism of action fails to explain the range of transcriptional responses that the WNT>β‐catenin axis triggers in different cellular contexts. Far from dissenting on the central role of β‐catenin, here we will argue for the existence of “extra‐canonical” mechanisms via which β‐catenin regulates transcription. We hypothesize that what we refer to as canonical Wnt signaling is a set of tissue‐ or cell‐specific entities scaffolded by key preserved features, and how cells respond to extracellular WNTs might depend on the transcriptional machinery that they express. As we will describe, a conspicuous number of nuclear proteins is directly involved in the regulation of Wnt target genes. Different combinations of their usage might contribute in explaining the tissue‐ or cell‐specific nature of the final message delivered by stabilized β‐catenin.

Hence, while we do not exclude that many upstream Wnt signaling components could contribute to the observed variability, our purpose is to focus on the interplay between β‐catenin and other nuclear factors. We will likely not be able to provide a final solution to this conundrum; on the other hand, we hope we will succeed in exposing some of the missing pieces required to complete the nuclear Wnt signaling puzzle.

## 2 OVERVIEW OF WNT SIGNALING—BUILDING THE CANON

### 2.1 An oncogenic extracellular signal

Possibly the first discovery of the Wnt field of research occurred in 1982, when the genomic locus *int‐1* was discovered (Nusse & Varmus, 1982). *int‐1* was identified as a common proviral integration site for the mouse mammary tumor virus (MMTV), an oncogenic murine retrovirus. *int‐1* was initially simply considered as a novel proto‐oncogene. In the following years the structure and sequence of the gene were elucidated, and its homology with the *Drosophila melanogaster* segment‐polarity gene *wingless* (*wg*) exposed, suggesting a signaling function in the development of multicellular organisms (McMahon & Moon, 1989; Nusse, Van Ooyen, Cox, Fung, & Varmus, 1984; Rijsewijk et al., 1987). These discoveries inaugurated what would truly become a pillar of two fields of research, developmental and cancer biology. With time, many genes related to *int‐1* were discovered (Gavin, McMahon, & McMahon, 1990), and at the beginning of the 1990s a revised nomenclature was established, in which the hybrid name “Wnt” was chosen to represent the *int‐1*/*wingless* gene family (Nusse et al., 1991).

Milestone studies revealed that downstream components along the Wnt pathway, and not the *WNT* genes themselves, were often altered in several types of human cancer. Notably, genetic analyses identified mutational hotspots in the adenomatous polyposis coli (*APC*) gene in patients with colorectal cancer (CRC) (Groden et al., 1991; Kinzler et al., 1991). APC was subsequently found to exhibit physical interaction with the recently characterized membrane protein β‐catenin. This observation provided two important new connections for the Wnt pathway: a mechanistic one concerning the role of APC in the cascade, and the relevance of Wnt signaling for CRC onset (Rubinfeld et al., 1993; Su, Vogelstein, & Kinzler, 1993). We now know that APC is part of a multi‐protein complex—the so‐called “destruction complex”—responsible for the uninterrupted β‐catenin degradation in the absence of WNT signal molecules. In addition to APC, this complex consists of other components, including AXIN, glycogen synthase kinase 3 (GSK3), casein kinase 1 (CK1), and protein phosphatase 2A (PP2A). For an extensive description of the destruction complex we refer to the elegantly written review by van Kappel & Maurice in 2017.

As previously mentioned, cytosolic β‐catenin is rapidly marked by phosphorylation at specific amino‐terminal Serine and Threonine residues (Ser33, Ser37, Thr41, and Ser45; for a complete list see fig. 4 in Valenta, Hausmann, & Basler, 2012) by the destruction complex: phosphorylation is the first step to target β‐catenin for degradation by the proteasome. β‐catenin was first implicated in Wnt signaling in *Drosophila melanogaster* by the observation that Arm protein abundance (Arm is the name of β‐catenin in Drosophila) was sensitive to the Wingless (Wg; WNT in flies) ligands secreted from nearby cells (Peifer et al., 1991; Riggleman, Schedl, & Wieschaus, 1990).

The Wnt/β‐catenin pathway is activated only upon WNT ligands reaching target cell. They bind to the extracellular portion of the Frizzled transmembrane receptors (Bhanot et al., 1996) and the Arrow/LRP co‐receptors (Wehrli et al., 2000). When this happens, the destruction complex is suppressed. This occurs in ways that are not fully understood but that involve polymerization of Disheveled (DSH) (Schwarz‐Romond et al., 2007). Newly synthesized β‐catenin is therefore no longer degraded, it accumulates in a free cytosolic form and translocates to the nucleus (Funayama, Fagotto, McCrea, & Gumbiner, 1995; Li et al., 2012). But how nuclear β‐catenin ultimately influences cell fate—and sometimes causes cancer—still remained to be determined.

### 2.2 From signal to transcription

The T Cell Factor/Lymphoid Enhancer Factor (TCF/LEF) is a family of four transcription factors (TFs) named TCF7, LEF1, TCF7L1, and TCF7L2 (previously designated as TCF1, LEF1, TCF3, and TCF4, respectively). They had been formerly discovered as important regulators of gene expression in immune cells, neural crest, tooth germs, and whisker follicles during embryogenesis (Brantjes, Roose, van de Wetering, & Clevers, 2001; Waterman, Fischer, & Jones, 1991). The identification of the TCF/LEF family members as key TFs that associate with nuclear β‐catenin was a momentous discovery, filling an enormous conceptual gap in the pathway (Behrens et al., 1996; Huber et al., 1996; Molenaar et al., 1996). The β‐catenin/TCF partnership is so evolutionarily ancient that an identical mechanism is also found in insects (Brunner, Peter, Schweizer, & Basler, 1997; van de Wetering et al., 1997). Mechanistically, in the absence of stabilized β‐catenin (that is in “WNT‐OFF” condition), TCF/LEF associate with the transducing‐like enhancer of split (TLE)/Groucho repressors (Brantjes et al., 2001). By recruitment of histone acetylases (HDACs) to this complex, gene expression is silenced through chromatin condensation (Sekiya & Zaret, 2007) (see Box 1 for chromatin regulation). Upon translocation into the nucleus (in “WNT‐ON” condition), β‐catenin is thought to displace TLE/Groucho and interacts with TCF/LEF: this is generally considered to be sufficient for de‐repression of target genes (van de Wetering et al., 2002). Strong transcriptional activation is subsequently achieved by the recruitment of additional co‐factors that were discovered in the following years (see Section 4).

BOX 1 **The catch‐22 of genomic regulation**

DNA is not a static molecule and its spatiotemporal activity and accessibility is highly regulated. In eukaryotic cells, DNA is tightly rolled up around nucleosomes, octamers of histone proteins wrapped within ~147 bp of DNA. This packaging is nonrandom and is an important determinant of dynamic gene expression. Genomic regions can be made looser (euchromatin) or tighter (heterochromatin) via post‐translational modifications (e.g., ubiquitinylation/acetylation/methylation) of the histone proteins. As a consequence, DNA is more or less accessible to transcriptional regulators, respectively. When accessible, transcription factors can make contact with the regulatory regions of specific genes, such as promoters, enhancers, and insulators, and turn genes ON and OFF (Klemm, Shipony, & Greenleaf, 2019). Certain regions of the mammalian genome contain clusters of functionally grouped enhancers, the “super‐enhancers.” Super‐enhancers constitute a platform for the converging action of signaling pathways to regulate genes that control cell identity during development (Hnisz et al., 2015). In addition, long‐range DNA–DNA interactions occur between distal chromatin regions defining loops of co‐regulated DNA stretches with an average size of 1 Mb known as “topologically associated domains” (TADs), whose position is conserved between cell types (Remeseiro, Hörnblad, & Spitz, 2016). TADs also provide strong boundaries to insulate super‐enhancers for their usage as functional units (Gong et al., 2018).

Here is the catch‐22: is this hierarchical chromatin organization a constraint that determines which groups of genes can be turned on by a signaling pathway? Or rather, do signaling cascades mold the chromatin structure genome‐wide and this has the consequence of turning genes ON and OFF?

**FIGURE 1:**
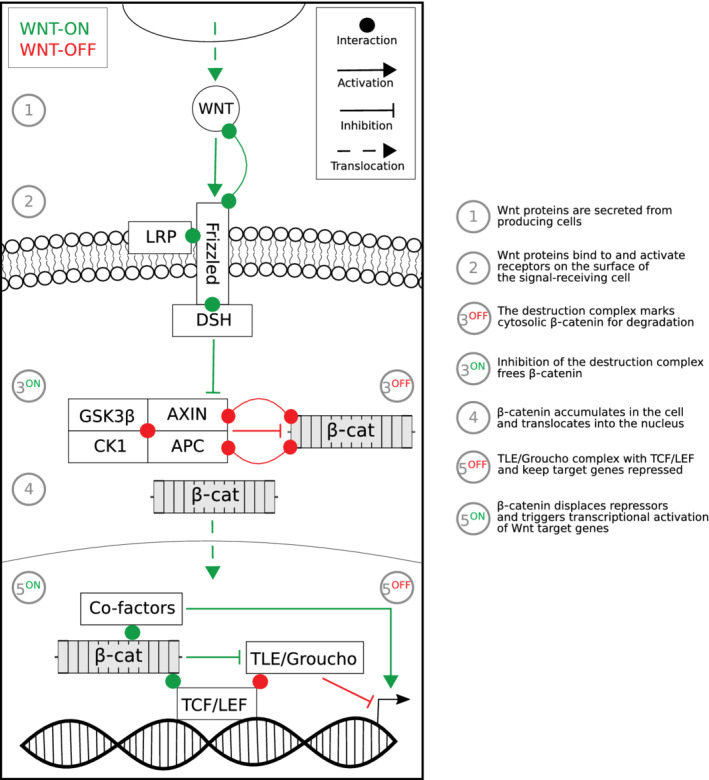
Schematic representation of the canonical Wnt signaling pathway. Key events along the pathway are numbered within the figure and described in the legend. The different types of relation between the pathway components are listed in the legend in the top‐right corner, and are represented in the diagram in red if they occur in the OFF state (when WNT ligands are absent), and in green if they take place in the ON state (when WNT binds to the receptor). Briefly, WNT proteins are produced and secreted by a cell. Upon reaching target cell, the WNT proteins trigger a cascade of intracellular events when they contact the Frizzled receptor and LRP co‐receptor located at the membrane surface. The receptor complex recruits the cytosolic protein Disheveled (DSH), which plays an important role in the inhibition of the β‐catenin destruction complex, composed by GSK3, AXIN, CK1, and APC. Free cytosolic β‐catenin is commonly phosphorylated by the destruction complex and thus marked for degradation. When the destruction complex is inhibited, increased levels of β‐catenin causes its translocation to the nucleus. Here, β‐catenin physically binds to the TCF/LEF family of transcription factors and is thought to displace the TLE/Groucho co‐repressors via not completely understood mechanisms. β‐catenin subsequently recruits a series of transcriptional co‐factors required for the activation of Wnt target genes

A more detailed account of the milestone discoveries in the field can be found elsewhere (van Amerongen, 2020; Wiese, Nusse, & van Amerongen, 2018). At the start of the 21st century, a coherent model of the Wnt signaling pathway, including explanations of the nuclear activity of β‐catenin, appeared to have taken a mature shape (Figure 1).

### 2.3 Who is β‐catenin?

β‐catenin belongs to a group of cell–cell adhesion catenin proteins, initially identified by the Kemler group as physical interactors of the integral to membrane molecule E‐cadherin/Uvomorulin, and their main role is to sustain epithelial integrity (Ozawa, Baribault, & Kemler, 1989). It is interesting to note that β‐catenin had just been previously discovered in Drosophila as the product of a gene affecting the patterning of the larval cuticle when mutated, in the remarkable Nobel prize‐winning screen for identifying developmental regulators (Jürgens, Wieschaus, Nüsslein‐Volhard, & Kluding, 1984). The dual structural/signaling nature of this protein became apparent when it was shown that the *CTNNB1* gene, encoding for the structural β‐catenin protein, was the mammalian homolog of the embryologically‐relevant Drosophila segment polarity gene *armadillo* (*arm*) (Mccrea, Turck, & Gumbiner, 1991).

Among the first observations connecting β‐catenin to Wnt signaling was the realization that Arm/β‐catenin protein abundance was sensitive to the *wg* gene product (Wg/WNT) secreted from adjacent cells in developing Drosophila embryos (Peifer et al., 1991; Riggleman et al., 1990). Moreover, the striking similarity of phenotype induced by mutations of *arm* and *wg* in Drosophila embryo and imaginal discs (Peifer, Sweeton, Casey, & Wieschaus, 1994), together with a wealth of epistasis analyses (see Box 2 for the concept of epistasis) in Drosophila consolidated the gene product of *arm* as a necessary downstream player of the Wg/Wnt pathway (Noordermeer, Klingensmith, Perrimon, & Nusse, 1994). Key was also the observation that β‐catenin overexpression in *Xenopus laevis* embryos induced the formation of a complete secondary body axis, assay that became a classical readout of active Wnt signaling (Funayama et al., 1995). Accumulation of β‐catenin and the consequent axis duplication were accompanied by the unexpected nuclear translocation of β‐catenin (Funayama et al., 1995), providing a first hint that this protein could be key to explain how extracellular WNT signals are transduced to the cell nucleus.

BOX 2 **The epistatic flatland**

Epistasis refers to a genetic phenomenon that reveals functional relationships between genes. If a loss‐of‐function mutation in gene *B* masks the phenotypic consequence of a variant of gene *A*, we infer that *B* is required after *A* in a cascade of events. Conversely, gain‐of‐function of *B* would produce a specific phenotype in a dominant manner, regardless of the status of *A*. We conclude that the product of *A* acts “upstream” of the gene product of *B* which is required “downstream.” This type of evidence is fundamental to understand the order in which biochemical and signaling events occur; epistasis experiments are among the most powerful tools in the hands of developmental biology, biochemistry and genetics (Jenny & Basler, 2014; Wiese et al., 2018). As a consequence of this modality of discovery, signaling pathways are conceived and represented as domino‐like, unidimensional series of events. We speculate that this might be a simplistic view. It is possible that different cells express distinct arsenals of proteins that cooperate in a combinatorial manner to generate varying responses to the same extracellular signal. A single upstream stimulus could then result in a spectrum of predictable outcomes that ultimately depends on the precise combination of factors initially expressed in the cell. We believe that the number of possible permutations of proteins assisting nuclear β‐catenin might contribute to explain the cell‐specific nature of the final message delivered by WNT molecules. As an open‐eyed inhabitant of Edwin Abbott Abbott's *Flatland*, a unidimensional Wnt/β‐catenin signaling might conquer new dimensions of complexity, in space (across organisms/cell‐types/disease) and time (throughout developmental/differentiation stages).

Several lines of evidence showed that the two functions of β‐catenin co‐exist in epithelial cells. Mouse Embryonic Stem Cells (mESC) lacking β‐catenin maintain pluripotency but fail to generate meso‐endodermal and neuroepithelium layers. A signaling‐defective β‐catenin rescues the *adherens junction* formation and the ability of mESC to differentiate into endoderm and neuroepithelium, but not into mesoderm (Lyashenko et al., 2011). The two functions of β‐catenin have also been uncoupled in vivo by specific mutations in the *arm*/*Ctnnb1* coding sequence, and can be studied separately in model organisms (Orsulic & Peifer, 1996; Valenta et al., 2011). For the reader interested in the many “faces and functions” of β‐catenin in addition to that during transcription, we refer to the rich and exhaustive review from Valenta and colleagues. The aim of this review is to revisit the ensemble of mechanisms that revolve around β‐catenin nuclear action and make possible the specific activation of Wnt target genes.

### 2.4 The two‐fold “handicap” of β‐catenin

**FIGURE 2:**
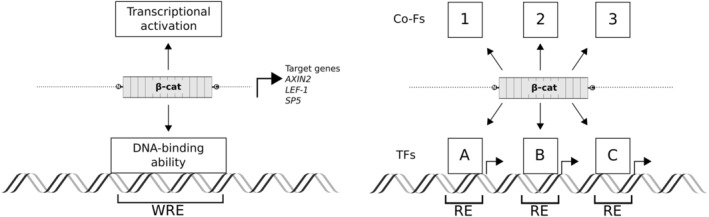
The two‐fold handicap of β‐catenin. β‐catenin is considered central for the activation of Wnt target genes. Yet, it lacks both DNA‐binding ability and a transcription trans‐activating domain. Great efforts have been devoted in the last two decades to understand how β‐catenin solves these shortcomings so that its action results in exclusive association to WRE (Wnt responsive elements) and specific Wnt target gene expression (left panel). The solution must rely on the existence of transcription factors (TFs) that permit specific association of β‐catenin on select regulatory elements (RE), and the recruitment of co‐factors (Co‐Fs) capable of open the chromatin and assist RNA polymerase II–mediated transcription (right panel; Box 1). As it will be discussed, the rigid scaffold structure of β‐catenin allows simultaneous anchorage of multiple protein complexes. In theory, different combinations of TF/Co‐F (e.g., from the figure: A1, A2, A3, B1, B2 et cetera) could complex with β‐catenin to stimulate tissue‐ or cell‐specific gene expression programs. As we will see, the TCF/LEF TFs are the main players conferring β‐catenin its functions (Section 3). However, β‐catenin is found to engage with several other TFs and Co‐Fs in various contexts, and this might contribute in explaining the tissue‐specific outcomes downstream of the Wnt signaling

The central role of β‐catenin in the assembly of the nuclear transcriptional complex downstream of WNT signals, recently referred to as Wnt enhanceosome (Fiedler et al., 2015; van Tienen et al., 2017), is now established. In fact, talking about the Wnt‐dependent transcription is tantamount to describing the nuclear function of β‐catenin. Interestingly however, β‐catenin protein does not possess precisely the two features that are required for gene regulation: (1) DNA binding ability and (2) transactivation potential (Figure 2). The solution to these two mechanistic riddles will be discussed separately in Sections 3 and 4, respectively. We will treat these as two independent problems, as each of them demands its own solution. We will see that a quasi‐universal explanation has been identified for Problem 1: how β‐catenin achieves DNA‐binding specificity. This is based on the central role of the TCF/LEF family of TFs—a rule that seems to account for a number of exceptions whose quantitative contribution remains unclear. In Section 4, we will revisit the identification of the many co‐factors that interact with β‐catenin and grant transcriptional activation potential to it. In antithesis with the universality of the TCF/β‐catenin association, a clear picture of the complete armamentarium of proteins that β‐catenin musters onto regulatory regions is still missing. Whether a “ubiquitous” solution to this problem exists in the first place, is an important question we desire to raise. Perhaps a universal transcriptional complex is elusive precisely because β‐catenin manages tissue‐specific complexes that assist and coordinate cell‐specific gene expression programs downstream of WNT signals.

## 3 β‐CATENIN SITS ON DNA: TCF/LEF DEPENDENT AND INDEPENDENT REGULATION

### 3.1 β‐Catenin and TCF/LEF: A silver wedding anniversary

The observations that β‐catenin physically interacts with the architectural TF LEF1 was literally a breakthrough discovery, for it provided the first explanation of how β‐catenin could get in contact with DNA at specific genomic locations (Behrens et al., 1996; Huber et al., 1996; Molenaar et al., 1996). Evidence of direct interaction between these two proteins was accompanied by other measurable functional readouts. First, epistasis analysis (Box 2) in Drosophila positioned Pangolin (Pan; the only TCF/LEF ortholog in flies) functionally downstream of Arm/β‐catenin (Brunner et al., 1997; van de Wetering et al., 1997). Second, microinjection in *Xenopus laevis* embryos of *LEF1* or *xTcf3* mRNA resulted in (i) nuclear translocation of β‐catenin, (ii) activation of target genes, and (iii) body axis duplication—a sign of aberrantly activated Wnt pathway (Behrens et al., 1996; Funayama et al., 1995; Huber et al., 1996; Molenaar et al., 1996). The “marriage in the nucleus” between β‐catenin and TCF/LEF was celebrated (Cavallo, Rubenstein, & Peifer, 1997), conferring to the β‐catenin/TCF complex the status of bipartite TF necessary for the activation of canonical Wnt target genes (Cadigan & Waterman, 2012; Nusse & Clevers, 2017).

The four TCF/LEF TFs share an evolutionarily conserved High‐Mobility‐Group (HMG)‐box DNA‐binding domain that displays remarkable amino‐acid sequence conservation (ca., 95–99% sequence identity). β‐catenin binds their N‐terminus, which is also considerably conserved (amino‐acids 1–50; ca., 60% sequence identity). It is therefore not surprising that TCF/LEF sustain overlapping functions (Cadigan & Waterman, 2012). On the other hand, it was shown that they also possess β‐catenin‐dependent diverging activities. For example, in mESCs, TCF7L1 is mainly considered a repressor and it is required to counteract the activation of target genes mediated by TCF7 (Molenaar et al., 1996; Yi et al., 2011). However, despite the different transactivation capacities, TCF7L1, and TCF7 are alone sufficient to guarantee mESCs tri‐lineage differentiation capability, indicating genetic redundancy (Moreira et al., 2017). Nonoverlapping TCF/LEF functions have also been recently suggested in a meta‐analysis of gene expression data from human CRC transcriptomics datasets. The expression of individual *TCF7*, *LEF1*, *TCF7L1*, and *TCFL2* genes correlated with transcriptomes that are both quantitatively and qualitatively different, supporting branching transcriptional regulation of Wnt target genes by different TCF/LEF during CRC progression (Mayer, de La Giclais, Alsehly, & Hoppler, 2020). Many might be the additional functions that TCF/LEF sustain, but their central role in the transduction of the Wnt/β‐catenin pathway holds true even when challenged with the scrutiny of state‐of‐the‐art high‐throughput approaches—with only a few exceptions (see Section 3.3).

#### 3.1.1 Never interfere between husband and wife: Regulators of the TCF/β‐catenin interaction

As in all good tales, there are several antagonists to the TCF/β‐catenin partnership. Attempts in identifying novel β‐catenin interactors revealed the existence of proteins whose primary role seems precisely that of attenuating the TCF/β‐catenin binding by physical interference. This is the case of the inhibitor of β‐catenin and TCF (ICAT), which binds to the region of β‐catenin spanning from repeat 10 to repeat 12 (Tago et al., 2000). ICAT repressed a constitutively active version of β‐catenin by impeding its interaction to TCF7L2, both on a transcriptional reporter in vitro and in Xenopus embryos in vivo. Here ICAT induced a ventralized phenotype, a recognized readout of loss or reduced Wnt signaling (Brannon, Gomperts, Sumoy, Moon, & Kimelman, 1997). Another nuclear protein, Chibby (Cby), was identified through protein–protein interaction screens in yeast and human HEK293T cells as yet another interactor of the β‐catenin C‐terminus (Takemaru et al., 2003). The results suggested that Cby competes with TCF/LEF, thereby downregulating β‐catenin‐mediated transcription. Also, inhibition of Cby in Drosophila embryos led to segment polarity defects that mimic a strong Wg/WNT gain‐of‐function. While the existence of proteins that dampen the TCF‐β‐catenin interaction betrays the cellular requirement of subtle fine‐tuning the activation status of this cascade, their role is not yet fully understood. It is clear however, that their expression influences Wnt/β‐catenin dependent cancer cells behavior, and makes these proteins attractive predictors of clinical outcomes or even therapeutic targets (Fan et al., 2019; Tsai et al., 2020).

The *TCF/LEF* genes themselves produce antagonizing isoforms that limit Wnt signaling by preventing β‐catenin binding. For instance, *LEF1* and *TCF7* loci both possess an alternative promoter in the second intron that leads to a 5′‐terminally truncated mRNA that precisely lacks the sequence encoding for the β‐catenin‐binding domain (Hovanes et al., 2001; Van de Wetering, Castrop, Korinek, & Clevers, 1996). These isoforms are referred to as “dominant negative” dnLEF1 and dnTCF7 (note that this does not entirely fit the definition of dominant negative that commonly designates mutations causing impairment of dimers formation). Importantly, dnLEF and dnTCF prevent activation of Wnt targets by binding to Wnt Responsive Elements (WREs) on the DNA without allowing β‐catenin association to buttress an activator transcriptional complex (Blauwkamp, Chang, & Cadigan, 2008). dnLEF and dnTCF are often co‐expressed with their full‐length counterparts and might be important to moderate the influence of Wnt signaling on cell growth. For instance, evidence supporting this derives from the quasi‐paradoxical observation that loss of TCF7 in mice is accompanied by the formation of mammary gland adenomas and polyp‐like intestinal neoplasms. This suggested that a variant encoded by the *TCF7* locus counteracts oncogenesis thus behaving as tumor suppressor (Roose et al., 1999). Consistently, circa 80% of human CRC display aberrantly expressed LEF1, while the promoter driving the dnLEF1 isoform appears actively suppressed (Li et al., 2006). Overall, the expression of dominant‐negative forms of TCF/LEF may be a general feature of these loci, used to moderate the effects of Wnt signaling in several developmental and lineage differentiation contexts.

Also, positive regulators of the TCF/β‐catenin interaction exist, such as the Ring Finger Protein 14 (RNF14). RNF14 was found to interact with TCF/LEF and promote Wnt signaling both in vivo in zebrafish and in human CRC cells, where it is required for their survival (Wu et al., 2013). As RNF14 is a E3 ubiquitin ligase that acts on unknown substrates, it has been speculated that RNF14 enzymatic activity might induce a conformational change of TCF/LEF that renders it prone for stable interaction with β‐catenin (Cantù, Valenta, Basler, 2013). TCF/LEF function is in fact affected by post‐translational modifications also in other contexts (Yamamoto, Ihara, Matsuura, & Kikuchi, 2003). For example, the poly(ADP‐ribose) polymerase 1 (PARP1) physically interacts with the β‐catenin/TCF7L2 module and functionally potentiates its transcriptional activity in CRC likely via PARylarion (Idogawa et al., 2005), or the transducin β‐like protein 1 (TBL1), which seems to recruit β‐catenin to Wnt target‐gene promoters for transcriptional activation during oncogenesis (Li & Wang, 2008). It is plausible that these coactivators are relevant in certain tissues or cell types where they modulate the intensity of Wnt signaling ad hoc to influence the balance between proliferation and cell differentiation.

### 3.2 β‐Catenin commits adultery

**FIGURE 3:**
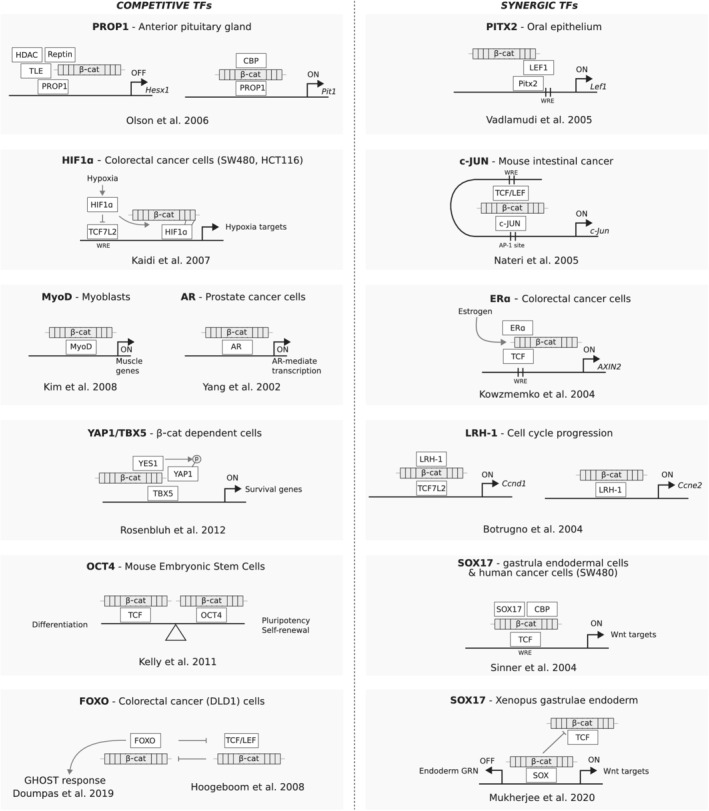
Alternative transcription factors (TFs) that team up with β‐catenin. The TFs are arbitrarily divided in two categories: those that compete with TCF/LEF for β‐catenin association (left column) and those that cooperate with TCF/LEF and β‐catenin in a ternary complex (right column). Each model is individually enclosed in a gray box to schematically reflect the discovery of its mechanism. Within each box we indicate the name of the TF and the main tissue/cellular model used for the discovery. Below each model we provide the reference of the relevant article. Activation events are indicated by a green arrow, while inhibition is represented with red arrows lacking arrowheads. Phosphorylation is displayed as a circled “P” connected to a target protein. WRE, Wnt responsive element

Several TFs other than TCF/LEF have been found to “flirt” with β‐catenin. This is a controversial aspect in the field, as in some cases the observation of a novel interacting TF stood up as a challenge to the singularity of the TCF‐β‐catenin “central dogma". Below we will list the most relevant examples known to date. They are here crudely divided in two groups: alternative TFs that compete with TCF/LEF (Section 3.2.1), and TFs that synergize with TCF/LEF (Section 3.2.2) All these interactions are also schematically represented in Figure 3.

#### 3.2.1 Alternative partners for β‐catenin

PROP1—In differentiating cells of the anterior pituitary gland, β‐catenin interacts with the cell‐lineage specific homeodomain factor PROP1—instead of TCF/LEF (Olson et al., 2006). It is proposed that the β‐catenin‐PROP1 couple acts as a “binary switch” that activates the expression of the pituitary lineage‐determining gene *Pit1*, while simultaneously repressing the lineage‐inhibiting factor *Hesx1*. This happens in response to WNT ligands and depends on β‐catenin. However, it cannot be defined as canonical Wnt signaling, for in this context PROP1 competes with TCF/LEF for β‐catenin binding. β‐catenin/PROP1 and β‐catenin/TCF regulate nonoverlapping sets of genes. The competitive nature of this relationship is also indicated by the finding that the armadillo repeats 5–9 is sufficient to mediate the interaction with PROP1, and this partially overlaps with the TCF/LEF interaction domain (Behrens et al., 1996). This binary switch mechanism in pituitary gland cells constitutes a way for generating diverse cell types from pluripotent precursor cells in response to a common upstream WNT signal. Perhaps other tissues employ tissue‐specific homeodomain TFs to the same purpose?

HIF1α—During hypoxic conditions, the oxygen‐sensitive hypoxia‐inducible factor‐1α (HIF‐1α) increases in abundance and competes with TCF7L2 for direct binding to β‐catenin. Here, β‐catenin‐HIF1α regulates classical HIF1 target genes that ultimately promote cell survival and adaptation to hypoxia. β‐catenin physically associates with HIF‐1α on target loci, and enhances HIF‐1α‐mediated transcription by a mechanism not entirely elucidated (Kaidi, Williams, & Paraskeva, 2007).

MYOD1—Genetic evidence indicated that while β‐catenin is required for mouse C2C12 myoblasts differentiation into multinucleated myotubes, both TCF/LEF as well as the cell adhesion function of β‐catenin are fully dispensable for this process (Kim, Neiswender, Baik, Xiong, & Mei, 2008). The authors show that, in this context, β‐catenin interacts directly with the basic helix–loop–helix transcription factor MYOD1. MYOD1/β‐catenin regulates genes by binding to consensus‐specific E box elements on the DNA and are both essential for muscle cell differentiation (Santoro, Yi, & Walsh, 1991). It is surprising that a tissue‐specific master regulator of cell fate, such as MYOD1, requires β‐catenin for transcriptional transactivation as if β‐catenin acts as a basal factor for the RNA Polymerase II (RNAPII) activity. But this is supported by compelling lines of evidence: mutations of the E box elements in transcriptional reporters abolished β‐catenin dependent potentiation of MYOD1 activity. Moreover, MYOD1 transcription is potently reduced when its interaction with β‐catenin is disrupted (Kim et al., 2008).

AR—The androgen receptor (AR) is a type of intracellular receptor activated by the binding of androgenic hormones, such as testosterone and dihydrotestosterone. When found by its ligand in the cytoplasm, AR translocates to the nucleus where it acts as TF: it binds DNA and regulates gene expression (Kokal, Mirzakhani, Pungsrinont, & Baniahmad, 2020). In prostate cancer cells, β‐catenin was shown to selectively bind to AR in an androgen (but not estrogen) dependent manner. Transfection of β‐catenin in prostate cancer cells augmented the ligand‐dependent transcription downstream of AR. Intriguingly, reconstituted expression of E‐cadherin in E‐cadherin‐negative prostate cancer cells caused the redistribution of β‐catenin to the cell membrane, and consequent reduction of AR‐dependent activity (Yang et al., 2002). Therefore, β‐catenin can sustain malignant cell behavior in prostate cancer cells in a TCF/LEF‐independent manner (Cronauer, Schulz, Ackermann, & Burchardt, 2005).

YAP1/TBX5—In a powerful screen for identifying essential genes in human cancer cell lines whose survival depends on β‐catenin, Rosenbluh et al. (2012) found that the YES‐associated protein 1 (YAP1) and the T‐box containing TF TBX5 are needed for the survival of these cells. Mechanistically, YAP1 and TBX5 form a ternary complex with β‐catenin, independently from TCF/LEF. Phosphorylation of YAP1 by the tyrosine‐kinase YES1 leads to localization of this complex to the promoter of antiapoptotic genes (e.g., *BCL2L1* and *BIRC5*) allowing cell propagation and resistance to apoptosis‐inducing stimuli. These observations might entail that this ternary complex could be essential for transformation and survival of several others Wnt/β‐catenin‐driven tumors.

OCT4—The Doble group identified a mechanism that regulates the balance between differentiation and renewal in mESCs. Under conditions where the signaling pool of β‐catenin is stabilized, a fraction of it associates with the pluripotency TF OCT4 as an alternative to TCF/LEF. While β‐catenin coactivates the expression of different sets of genes in concert with either TCF/LEF or OCT4, it is the β‐catenin‐OCT4 partnership that sustains mESC pluripotency. Consistently, attenuation of the β‐catenin/TCF branch, via overexpression of a dnTCF protein variant, impairs differentiation into all germ layer lineages but enhances retention of pluripotency markers (Kelly et al., 2011).

FOXO—While the FOXO TFs constitute an interesting ample chapter (see below, Section 3.3), it is worth mentioning it in this category. FOXO proteins compete with TCF/LEF for binding to β‐catenin (Hoogeboom et al., 2008). Reduced binding between TCF/LEF and β‐catenin is observed upon *FOXO3a* and *FOXO4* overexpression, and small interfering RNA‐mediated knockdown of these genes releases β‐catenin to enhance TCF/LEF transcription. β‐catenin might be the pivot of a new nuclear swapping between the FOXO and TCF/LEF pathways (Essers et al., 2005).

#### 3.2.2 More than one at the same time: A polygamous β‐catenin

PITX2—In the region of the oral epithelium from which teeth will develop, *LEF1* expression is sequentially required right after the activity of the homeodomain TF PITX2 (Mitsiadis & Graf, 2009). In a nice piece of molecular biology, Vadlamudi and colleagues identified that it is precisely PITX2 that promotes *LEF1* transcription in the oral epithelium by activating its promoter, and it does so synergistically with β‐catenin, in a manner that is independent of the motifs present within classical WREs. Once produced, LEF1 protein complexes with PITX2‐β‐catenin in a ménage à trois that positively feeds back to *LEF1* gene activation, reinforcing the successive requirement for canonical Wnt signaling (Vadlamudi, 2005). Could this represent a more general mechanism in which cell specific TFs co‐regulate the expression of Wnt targets during epithelial differentiation?

JUN—In a similar fashion as observed for PITX2‐β‐catenin‐LEF1 on *LEF1* promoter, the proto‐oncoprotein c‐JUN interacts with TCF7L2 to activate *c‐JUN* promoter itself (Nateri, Spencer‐Dene, & Behrens, 2005). This occurs in human CRC cells (HCT116) but not in other contexts that do not display active Wnt signaling and/or nuclear β‐catenin, suggesting tissue‐specific action of this complex. Phosphorylated c‐JUN forms a ternary complex with TCF7L2 and β‐catenin, and genetic abrogation of c‐JUN in the ApcMin mouse model of intestinal cancer (in which tumors are essentially caused by an aberrantly activated Wnt pathway upon partial loss‐of‐function of *Apc*; Jackstadt & Sansom, 2016) caused reduced neoplastic size and prolonged life span, suggesting that c‐JUN protein has a central role in β‐catenin‐dependent intestinal tumorigenesis (Nateri et al., 2005).

ERα—The estrogen receptor alpha (ERα) immunoprecipitated β‐catenin in human CRC cells (also here HCT116 were used; Kouzmenko et al., 2004). The two proteins reciprocally enhanced the transactivation of cognate reporter genes (that is, β‐catenin promoted the transcription from estrogen responsive elements [ERE] while ERα enhanced β‐catenin dependent WRE activation). They were also cooperatively recruited to cognate response elements in the promoters of endogenous target genes. This was interpreted as the first direct evidence of crosstalk between Wnt and estrogen signaling pathways. It is interesting to note that ERα can also act as a co‐factor at non‐ERE sites via interaction with c‐JUN/c‐FOS on the AP‐1 DNA binding sites (Kushner et al., 2000). It is tempting to speculate that—and worthwhile testing if—this noncanonical activity of ERα is precisely mediated by the aforementioned interplay between c‐JUN and β‐catenin (Nateri et al., 2005).

LRH‐1—Liver receptor homolog 1 (LRH‐1) is an orphan nuclear receptor expressed in endodermal tissues and is involved in the control of cholesterol homeostasis. LRH‐1 induces cell proliferation in pancreatic and hepatic cells via the concomitant activation of both *CCND1* and *CCNE1* (encoding for cyclin D1 and E1, respectively; Botrugno et al., 2004). Compelling evidence points to the coexistence of two mechanisms both necessitating β‐catenin. First, LRH‐1 induces *CCND1* expression by acting as a coactivator for β‐catenin/TCF7L2—that is, without “touching” the DNA. Second, LRH‐1 directly binds *CCNE1* promoter via a conserved LRH‐1‐ responsive element, but also in this case it is nuclear β‐catenin responsible for the transcriptional activation (Botrugno et al., 2004; Yumoto et al., 2012). This case is exemplary, as it shows how within the same cells two mechanisms—one of cooperation and one of competition—can live side by side.

SOX17—Another class of TFs interacting with WNT signaling is the SOX family of TFs (Kormish, Sinner, & Zorn, 2009; Zorn et al., 1999). This constitutes an interesting case as SOX proteins, similarly to TCF/LEF, bind DNA via the high‐mobility‐group (HMG) box (Cantù et al., 2011). SOX and TCF/LEF seem to bind similar but distinct variants of the same core consensus sequence (Van Beest et al., 2000). One is tempted to hypothesize that SOX proteins could even compete for the WREs on DNA. As it happens, a functional interaction between SOX factors and Wnt signaling was first revealed in Xenopus embryos: SOX17 and SOX3 were found to inhibit β‐catenin activity by suppressing dorsal‐anterior axis formation. Their expression resulted in headless embryos, a classical readout of inhibited WNT activity (Kormish et al., 2009). Recent work identified that SOX17 and β‐catenin co‐occupy genome‐wide a number of enhancers during endoderm formation in Xenopus (Mukherjee et al., 2020). As predicted, SOX17 and β‐catenin synergistically activate transcription both in the absence as well as in the presence of TCF/LEF by interplay with different sets of enhancers (Mukherjee et al., 2020; Sinner et al., 2004). Mukherjee and colleagues elegantly suggest that these observations point to a novel paradigm where genomic specificity of β‐catenin binding is determined through interplay between lineage‐specific TFs, of which SOX could be just one example. As it happens for SOX proteins or LRH‐1, could the coexistence of two modes of action be a more general feature of the β‐catenin‐dependent transcriptional response?

### 3.3 Use of systematic approaches to test how frequent these affairs are

All the cases where β‐catenin cooperates unconventionally with other TFs have been discovered in select cellular models or when studying particular stages of development or cell lineage differentiation. It is therefore possible that each of them, rather than representing a general feature of nuclear Wnt signaling, only concerns how this particular cell type converts the WNT signal into a transcriptional output. In other words, whether they represent tissue‐specific or general mechanisms is still unknown and awaits clarification.

Recent studies attempted to tackle this problem in a systematic manner, making use of unbiased high‐throughput approaches. At the onset of the CRISPR/Cas9 era, the “canonicity” of the TCF‐β‐catenin interaction was for example probed in Drosophila Kc embryonic cells via mutagenesis followed by transcriptomics studies. In cell carrying loss‐of‐function mutations of *arm* (β‐catenin) or *pan* (TCF) the transcriptome‐wide gene expression pattern normally occurring upon Wg/WNT stimulation was dramatically amputated. Also the genomic Wg/Wnt‐responsive enhancer elements showed full dependency on the presence of Pan, as indicated by STARR (self‐transcribing active regulatory region)‐sequencing, a method to identify novel enhancers based on the activity of millions of candidates from a genomic DNA library (Arnold et al., 2013). These led the authors to reject the existence of alternative branches of the Wg/Wnt pathway (Franz, Shlyueva, Brunner, Stark, & Basler, 2017). But novel questions emerged from this study. A paradigmatic view of the Wnt pathway implies that, in the absence of Pan/TCF, target genes would become de‐repressed—that is, not subjected to active repression (van de van de Wetering et al., 2002). Of surprise, only a fraction (ca., 37%) of the positively regulated target genes were de‐repressed in *pan* null Kc cells. Also, some genes were expressed at lower levels in the absence of Pan, possibly suggesting that Pan/TCF might activate their transcription before any Wg/Wnt stimulus (Franz et al., 2017). This is interesting, yet it is still lacking a clear mechanistic explanation.

Schuijers, Mokry, Hatzis, Cuppen, and Clevers (2014) used chromatin immunoprecipitation coupled to massive parallel sequencing (ChIP‐seq) to test the genome‐wide interplay between TCF/LEF and β‐catenin binding sites and—due to the almost complete overlap between β‐catenin and one or the other TCF/LEF—they concluded that β‐catenin transcriptional activation is exclusively mediated by TCF/LEF. In this case, the authors identified two classes of β‐catenin binding sites based on TCF7L2 (referred to as TCF4 in the original article) signal intensity distribution. The first “β‐catenin‐TCF7L2‐high” class constituted 77% of the DNA‐bound β‐catenin and likely represents the main canonical β‐catenin activity. The second “β‐catenin‐TCF7L2‐low” class presented β‐catenin peaks that were associated neither with TCF7L2 nor with TCF7 or LEF‐1. Consistently, these peaks were often missing a TCF/LEF consensus binding motif and seemed not to be associated with transcribed genes (Schuijers et al., 2014). While these observations suggested that TCF/LEF cannot account for the full extent of β‐catenin recruitment, the authors do not find evidence supporting the presence of non‐TCF transcription factors. A dominant‐negative ΔNTCF7L2 protein was in fact capable of diminishing both classes of β‐catenin peaks. This study was carefully executed in three different model systems: (i) the crypts of the murine intestinal epithelium as Wnt signal dependent homeostatic proliferation model, (ii) LS174t colon cancer cells harboring Wnt‐activating mutations, and (iii) HEK293T embryonic kidney cells with a well‐characterized physiological Wnt‐response.

These high‐throughput studies largely support the paradigm of nuclear Wnt signaling that sees TCF/LEF as the prominent mediators of β‐catenin function. However, they also highlight novel unknown features of this pathway. The exploitation of state‐of‐the‐art technologies will allow accumulation of valuable datasets that will be merged in a system perspective analysis to fully understand if ever, and how frequently, β‐catenin engages in tissue‐specific circuits via selective interplay with other TFs.

#### 3.3.1 A β‐catenin GHOST response

In another recent study, HEK293T clonal cell populations lacking all four TCF/LEF factors were generated, enabling to test how the β‐catenin genomic occupancy (via ChIP‐seq) appears in the effective absence of all TCF/LEF TFs (Doumpas et al., 2019). While *TCF*/*LEF* quadruple knockout cells expectedly could not transduce canonical Wnt‐dependent transcription, β‐catenin preserved physical binding to a small fraction of its target genomic loci (ca., 30 out of 1,300) even in the absence of TCF/LEF. These target regions (by definition TCF/LEF‐independent) exhibited enriched consensus motifs of the Forkhead box‐containing (FOX) TFs, and genes in their vicinity were transcriptionally sensitive to FOXO4 downregulation. FOXO TFs (FOXO3a and FOXO4 in particular) have been previously implicated as partners of β‐catenin (Essers et al., 2005; Hoogeboom et al., 2008). It is conceivable that, in specific tissues, individual β‐catenin molecules must choose whether to team up with TCF/LEF or FOXO proteins; β‐catenin might fulfill a critical role in balancing TCF/LEF and FOXO signaling. Consistently with this observation, HEK293T cells exhibit a β‐catenin GHOST response (genes hidden outside standard targets) that occurred upon β‐catenin stabilization only when all the four TCF/LEF proteins are mutated. Of note, the GHOST response was in part mediated by FOXO4 (Doumpas et al., 2019). This response included the regulation of a group of targets genes that are sensitive to β‐catenin/FOXO4 activity only in the absence of TCF/LEF, or when physiological inhibitors of the β‐catenin‐TCF/LEF interactions, such as ICAT, were present (Doumpas et al., 2019; Hossain, Yu, Xu, & Sen, 2008). Admittedly, this activity might be generated by the artificiality of the set up: it might never naturally occur that all four TCF/LEF proteins are not expressed in a cell. On the other hand, it is plausible that the β‐catenin/FOXO‐GHOST response could physiologically compete with the β‐catenin‐TCF/LEF canonical activity depending on the local concentration of the β‐catenin‐TCF interaction‐modifiers such as ICAT, Cby, or the dnTCF/dnLEF (see Section 3.1.1; Hossain et al., 2008; Yokoyama, Pate, Sprowl, & Waterman, 2010). Of note, the WNT/FOX connection is far from being resolved, and new FOX TFs are currently being unraveled as activators downstream of WNT signals. Two recent examples are FOXH1 that, together with Nodal/TGFβ signaling, defines the coregulatory context for maternal (that is, after conception but before zygotic activity) β‐catenin physical association to its targets (Afouda et al., 2020), and FOXB2, that stimulates Wnt/TCF reporter and target genes without activating LRP6 and β‐catenin (Moparthi, Pizzolato, & Koch, 2019). The TCF/LEF versus FOX dilemma is also reminiscent of other exceptions mentioned above, such as the existence of a binary switch in differentiating pituitary gland cells occurring through divergent gene regulation by β‐catenin‐TCF/LEF versus β‐catenin‐PROP1 complexes (Olson et al., 2006). We speculate that a future identification of more than two competing TFs in the same cells will allow testing the existence of ternary (or N‐ary) choices, especially when cell lineage commitment does not occur upon subsequent binary branch points but rather along continuous trajectories (Velten et al., 2017). We speculate that in these cases, the versatility of the β‐catenin “switch” might favor some cell state transitions and lineage commitment combinations, rendering some more likely to occur than others.

## 4 THE β‐CATENIN HUB: A SCAFFOLD FOR TRANSCRIPTIONAL COMPLEXES

### 4.1 β‐Catenin in its puberty: A growing transcriptional complex

The companionship with TCF/LEF or other TFs provides β‐catenin with the competence to selectively recognize regulatory regions on the DNA. A long list of scientific articles was needed to address the other problem of how β‐catenin can forge a transcriptionally active locus. Initial hints derived from the original identification of TCF as main partner. It was found that while the N‐terminus of Arm/β‐catenin was required for this physical interaction, its C‐terminus was free to act as a transactivation domain (van de Wetering et al., 1997). However, the molecular mechanism underlying this behavior was not understood. Could β‐catenin directly position and activate RNAPII on transcriptional start sites (TSSs)? Early experiments were instructive for realizing that additional co‐factors were likely required for the expression of β‐catenin target genes. For instance, overexpressed β‐catenin and LEF‐1 were not sufficient to elicit significant activation of a transcriptional reporter despite their concomitant nuclear localization (Prieve & Waterman, 1999).

Possibly the first β‐catenin co‐factor identified, a 52‐kDa protein named Pontin52 (from Latin, *pons*, meaning bridge), seemed to provide a conclusive explanation. Pontin52 exhibited simultaneous binding to β‐catenin and TATA‐box binding protein (TBP), a basic component of the eukaryotic transcriptional machinery associated with RNAPII (Bauer, Huber, & Kemler, 1998). Curiously, Pontin52 binds to the N‐terminus of β‐catenin over a region spanning the Armadillo‐repeats 2–5. The N‐terminus, hence, brings together Pontin52 and LEF1 in a nuclear complex with TBP (Bauer et al., 1998). Notwithstanding, this did not explain the transactivation potential of the β‐catenin C‐terminus. Its mechanistic independence from the N‐terminus was subsequently revealed in protein‐deletion studies showing that it is the C‐terminus that, in particular, conferred strong and direct association with TBP (Hecht, Litterst, Huber, & Kemler, 1999). β‐catenin displayed a tight grasp on TBP with both its N‐terminal (via Pontin52) and C‐terminal arms.

These studies were of capital importance, as they connected for the first time the nuclear action of β‐catenin with the general machinery required for the RNAPII‐mediated transcription of genes. However, the interactions with Pointin52 and TBP were considered unlikely to be sufficient for β‐catenin activity. In fact, a β‐catenin deletion mutant that retained the armadillo repeats 1–9 failed in activating reporter expression even though it should be fully capable of binding Pontin52. Also TBP, though it is ubiquitously present, could not confer transactivation potential to the nuclear β‐catenin/TCF complex in all cellular contexts tested (Prieve & Waterman, 1999). The scavenger hunt to find the other elusive co‐factors required for β‐catenin's job had just started.

### 4.2 Histone acetylation at target genes

In two independent studies the C‐terminus of β‐catenin was brought into play, for it was found to interact with the histone acetyltransferase (HAT) CREB‐binding protein (CBP) and the closely related p300 (Hecht, Vleminckx, STemmler, van Roy, & Kemler, 2000; Takemaru & Moon, 2000). HAT enzymes had been recently discovered as important players in gene regulation. They contribute to chromatin opening at promoters by converting acetylated histone H3 and histone H4 into transcriptional adaptors, a process that permits access to additional transcriptional regulators (Verdone, Agricola, Caserta, & Di Mauro, 2006; see Box 1). Thanks to their HAT activity, CBP/p300 were supposedly requested for all β‐catenin outputs. Surprisingly however, CBP/p300 acted in a seemingly promoter‐specific fashion. In the same experimental setup, CBP/p300 potentiated the activation of the *siamois* promoter (*siamois* is a known Wnt target gene in Xenopus, encoding for a dorsally expressed homeobox TF; Brannon et al., 1997), while the *Ccnd1* promoter was refractory to CBP/p300 stimulation (Hecht et al., 2000). These findings may suggest that β‐catenin could activate transcription both in a CBP/p300‐dependent as well as CBP/p300‐independent manner. Based on their data, Hecht and colleagues define CBP/p300 as “a promoter‐specific coactivator of β‐catenin.” This observation is not much debated in recent years, yet it raised a question that still lacks a clear response. Is the Wnt/β‐catenin transcriptional complex varying depending on the target gene? Perhaps cells possess alternative machineries that drive the activation of individual Wnt‐sensitive promoters?

The role of CBP/p300 generated another conundrum in the field. Drosophila CBP was previously shown to regulate Wg/Wnt signaling negatively (Waltzer & Bienz, 1998). However, as we are seeing, overexpression of CBP in vertebrate model systems had an activating effect (Hecht et al., 2000; Takemaru & Moon, 2000). Overall, the consensus steered toward a crucial function as co‐activators for CBP/p300 via their HAT activity, to the point of even considering them as relevant therapeutic targets in Wnt driven cancers (Ludlam et al., 2002; Ma, Nguyen, Lee, & Kahn, 2005). It is possible however that the function of CBP (and perhaps other β‐catenin partners) diverges in different species. Alternatively, CBP may behave as a repressor in the absence of Wnt signals when it is recruited by TCF to shut down transcription, while it switches into a coactivator only upon β‐catenin binding (Takemaru & Moon, 2000). Accordingly, ChIP experiments in Drosophila revealed a CBP‐dependent widespread histone H3 and H4 acetylation—marking actively transcribed chromatin—present at Wnt target loci after stimulation (Parker, Ni, Chang, Li, & Cadigan, 2008; Box 1). Of note, while Pan/TCF, Arm/β‐catenin and methylation of histone H3 (H3K4me3) were found at specific locations close to the WREs, histone acetylation was broadly distributed even at a distance of 30 kb from the β‐catenin binding site. This activity depended on the presence of CBP, occurred in a short time (less than 5.5 hr), and took place also when transcription was blocked using the RNAPII inhibitor α‐amanitin (interestingly this prevented the Wnt‐dependent methylation of histone H3 together with transcription; Parker et al., 2008). This seminal study suggested that chromatin acetylation could be uncoupled by both transcription and chromatin methylation, and likely occurs before both.

### 4.3 Plowing the ground: Association with the SWI/SNF nucleosome repositioning complex

The HAT activity associated with CBP/p300 connected β‐catenin's signaling role with the regulation of the chromatin and its accessibility (Box 1). However, several lines of evidence indicated that this was not sufficient to set the stage for transcriptional activation. For example, β‐catenin mutants lacking the domain that binds CBP could still transactivate transcription (van de Wetering et al., 1997) and, as seen before, the cooperative action of CBP and β‐catenin only affected a subset of known Wnt target genes (Hecht et al., 2000). A two‐hybrid screen performed in the Clevers group used the Armadillo repeats 1–12 as bait against a library of human fetal brain cDNA to uncover a novel interaction between β‐catenin and the brahma‐related gene 1 (BRG‐1) (Barker et al., 2001). BRG‐1, also known as SMARCA4, is the central ATP‐dependent catalytic unit of the chromatin‐remodeling SWI/SNF (SWItch/Sucrose NonFermentable) complex. Nucleosomes have a central role in controlling gene expression as their presence generally prevents the binding of transcription factors. SWI/SNF uses the energy generated through hydrolysis of ATP to slide and dislodge nucleosomes, and promotes or represses transcription of genes by increasing or decreasing accessibility of DNA to transcriptional machineries in a locus‐specific manner (Griffin, Curtis, Davis, Muthukumar, & Magnuson, 2011). BRG‐1 overexpression doubled the β‐catenin induced TCF‐responsive gene activation driven by the *siamois* promoter. Consistently, stable expression of inactive forms of BRG‐1 in CRC cells inhibited the expression of endogenous Wnt targets. This effect appears to be evolutionarily conserved as reduction of *brahma* (the BRG‐1 orthologue in flies) in Drosophila suppressed the rough eye phenotype caused by activated Arm/β‐catenin (Barker et al., 2001). The role of BRG‐1 depended on its ATPase enzymatic activity: BRG‐1 lacking the catalytic domain inhibited β‐catenin signaling in a dominant‐negative fashion. Even though now established, the participation of SWI/SNF downstream of Wnt signaling demands additional scrutiny. Belying its seemingly universal role, this macromolecular complex consists of several variable subunits (reviewed in Mittal & Roberts, 2020). The ATPase BRG‐1/SMARCA4 is a core subunit, but no data available to date precludes the possibility that the variable components of the SWI/SNF play a locus‐specific function also in Wnt signaling. For example ARID1A, which only assembles with the canonical BRG1/BRM‐associated factor (BAF), has been often implicated in Wnt‐driven cancers (Heckl et al., 2018; Mathur et al., 2017; Mouradov et al., 2014; Renko et al., 2019), and several other canonical BAF components are simultaneously assembling on classical WRE (van Tienen et al., 2017). While the variable SWI/SNF parts have been associated with diverse forms of cancer (Mittal & Roberts, 2020), the relation of each of them to Wnt signaling remains to be elucidated.

### 4.4 New enzymatic activities—A requirement for methyl transferases

The list of β‐catenin interacting partners considerably grew with the advent of pull‐down approaches followed by mass spectrometry analyses. In a seminal study, several new interaction partners of the β‐catenin C‐terminus were identified in one shot by GST‐pulldown (Sierra, Yoshida, Joazeiro, & Jones, 2006). The new partners included the transcription/transformation domain‐associated protein TRRAP, the SNF2‐related helicase p400, the bacterial RuvB‐related DNA‐dependent ATPases TIP49a/Pontin52 (identified already in Bauer et al., 1998) and TIP49b/TIP48/Reptin, all of which are subunits of the TRRAP/TIP60 HAT complexes that have critical roles in transcriptional regulation (Cai et al., 2003). The pulldown fraction also included subunits from two other chromatin complexes: the Imitation Switch nucleosome‐remodeling ATPase ISW1, which is found in several distinct remodeling complexes, and the SET1‐type proteins mixed lineage leukemia MLL1 and MLL2. SET1 and MLL (also known as KMT2) are conserved from yeast to human and possess histone methyltransferase (HMT) activity. Their affinity is high for the major sites of lysine methylation on histone H3 and H4 tails. In particular, H3K4 trimethylation is associated with the promoter of actively transcribed genes (Del Rizzo & Trievel, 2011).

Via its multi‐partner interactions, β‐catenin is therefore juggling both HAT and HMT enzymatic properties. Intriguingly, in vitro experiments indicated that this complex at β‐catenin C‐terminus possessed low HAT but high HMT activity. Moreover, functional assays showed that it is the HMT activity that is critical for the activation of the target gene *c‐Myc*. It is interesting to note that, in this experiment, the authors could not identify the C‐terminally interacting HAT proteins CBP/p300 (Sierra et al., 2006). This is consistent with the transcription at other loci, such as *Ccnd1* that is activated in a CBP/p300‐independent fashion (Hecht et al., 2000). Is it possible that HAT and HMT complexes compete for β‐catenin binding? In alternative, could they mediate different stages of transcriptional regulation, from transcriptional start to RNAPII mediated elongation? These are still open problems, but we can conclude that β‐catenin contributes in the early stages of transcription by managing the allocation of a plethora of chemical modifications at target loci.

The requirement of HMT activity was also implied by the subsequent identification of the multisubunit Dot1 complex (DotCom), which includes several MLL players, TRRAP, and β‐catenin (Mohan et al., 2010). The human DotCom complex is enzymatically active and can catalyze H3K79 di‐ and tri‐methylation. Consistently, also in Drosophila the loss of DotCom‐associated proteins causes a reduction of H3K79 methylation and consequent turning off of Wg/Wnt targets (Mohan et al., 2010). Of note, from yeast to human, trimethylation of H3K79 and H3K4 in many instances requires monoubiquitylation of histone H2B (Vlaming et al., 2014), implying that ubiquitin‐addition is an enzymatic activity whose requirement precedes that of methylation.

### 4.5 Mediating β‐catenin complexes

As we have seen, β‐catenin engages the HAT activity of CBP/p300 to render the chromatin accessible, adopts SWI/SNF to reposition the nucleosomes and shuffle histones ad hoc, and induces methylation of WREs paving the way for RNAPII to initiate transcription. Often however, WREs are found in introns or in regions that are distant from the TSS (Jho et al., 2002). What directs all these activities coordinated by β‐catenin toward the right promoter? The answer to this riddle came from protein cross‐linking experiments in HeLa and HEK293 cells that identified the interaction of β‐catenin with a machinery widely required for RNAPII: the Mediator complex (Kim, Xu, Hecht, & Boyer, 2006). Mediator is an evolutionarily conserved multiprotein complex with modular organization of circa 30 subunits that provides the required interface to connect enhancer‐bound TFs with RNAPII and the general transcriptional machinery. Its activity occurs during the initiation phases of transcription (reviewed in Soutourina, 2018). Direct interaction between β‐catenin and the MED12 subunit of the Mediator complex seemed a predominant feature of a functional β‐catenin transcriptional complex (Kim et al., 2006). Both isolated MED12 and intact Mediator was shown to bind the β‐catenin C‐terminus, while disruption of the β‐catenin/MED12 interaction inhibited Wnt‐induced transactivation. Accordingly, loss of function of Mediator subunits caused several “Wnt phenotypes” in *C. elegans* and *Drosophila melanogaster* (Janody, Martirosyan, Benlali, & Treisman, 2003; Yoda, Kouike, Okano, & Sawa, 2005). Also in the mouse, genetic ablation of *Med12* induced embryonic lethality during gastrulation at 7.5 days post coitum (dpc), slightly after but similarly to loss of β‐catenin (Haegel et al., 1995). Of note, hypomorphic mutations of *Med12* delayed the lethality at 10.5 dpc, and caused cardiac, somitogenesis and neural tube closure defects compatible with concomitant impairment of canonical and noncanonical/PCP Wnt pathways (Rocha, Scholze, Bleiß, & Schrewe, 2010). Other studies also implicated MED12 in the modulation of hedgehog (Zhou, Kim, Ishii, & Boyer, 2006) and of transforming growth factor (TGF)‐β signaling (S. Huang et al., 2012). These data might indicate that Mediator is a general feature of the gene expression downstream of signaling pathways; nevertheless, MED12 remains an attractive therapeutic target for Wnt‐driven neoplastic tissues (Al‐Hendy et al., 2017).

### 4.6 Enters BCL9‐PYGO: A chain of adaptors to activate Wnt targets

The co‐factor Pygopus (Pygo) was simultaneously identified in four independent genetic screens carried out in Drosophila (Belenkaya et al., 2002; Kramps et al., 2002; Parker, Jemison, & Cadigan, 2002; Thompson, Townsley, Rosin‐Arbesfeld, Musisi, & Bienz, 2002). *pygo* loss‐of‐function caused a segment polarity phenotype reminiscent of strong mutations in *arm* or *wg* (Kramps et al., 2002; Thompson et al., 2002), implying that Pygo was necessary for transducing every canonical Wg/Wnt output. Genetic epistasis experiments (Box 2) placed Pygo downstream of Shaggy/GSK3 and at the same level of Arm/β‐catenin. However, Pygo does not directly bind the latter, but it requires the assistance of a “bridge” protein called Legless (Lgs). Lgs is a large unstructured protein that associates with Pygo via a N‐terminal homology domain 1 (HD1) (Kessler, Hausmann, & Basler, 2009; Kramps et al., 2002). Lgs simultaneously binds Arm/β‐catenin via another motif, the HD2, allowing the formation of a tripartite Arm‐Lgs‐Pygo module often referred to as “chain of adaptors,” in which each component recruits the next via dedicated protein–protein interaction surfaces (Städeli & Basler, 2005). Pygo has two vertebrate homologs, PYGO1 and PYGO2, that can potentiate Wnt signaling transcription in HEK293T (Kramps et al., 2002) and in CRC human cells (Thompson et al., 2002). Their downregulation in Xenopus by antisense deoxyoligonucleotides caused ventralization accompanied by a reduction of Wnt target genes expression (Belenkaya et al., 2002). Two evolutionarily conserved domains coordinate Pygo function: a C‐terminal PHD (plant homology domain) zinc‐coordinating finger that, as it occurs in other PHD‐containing proteins, confers affinity to methylated lysines on histone tails (such as di‐ and tri‐methylation of lysine 4 on histone H3, H3K4m2/3), and a N‐terminal homology domain (NHD) (Parker et al., 2002). The NHD contains a potent transactivation capacity that depends on a conserved NPF tripeptide: mutations of this motif abrogated the transcriptional capacity of Pygo in vitro and reduced Wg/Wnt signaling output in vivo in flies (Städeli & Basler, 2005). The NPF is also crucial for the association with the chromatin: experiments performed using the giant polytene chromosomes of the Drosophila salivary glands indicated that Pygo physically associates with WREs also in OFF‐pathway condition (de la Roche & Bienz, 2007). NPF mutant Pygo variants, on the other hand, displayed a considerably compromised association to these chromosomal locations. A molecular explanation of how this was achieved, and the partners of the NPF, remained however elusive. A suggestion came that, instead of being an activator, Pygo could operate as an anti‐repressor of the TCF/LEF‐Groucho/TLE complex. Mutating *groucho* in *pygo* mutant clones of cells in the Drosophila wing imaginal disc (to generate *groucho* and *pygo* double‐mutant clones) reinstated expression of the target gene *engrailed* (Mieszczanek, de la Roche, & Bienz, 2008). This would support that Pygo is not obligatory for transcription if the interaction between Groucho/TLE and Pan/TCF is depleted. Perhaps, by constitutively binding onto WREs together with Pan/TCF, Pygo predisposes the locus for rapid Wg/Wnt‐mediated activation. More recent evidence that we will describe later, point to a more complex function of the NPF in mediating the assembly of additional players within the transcriptional complex (Fiedler et al., 2015).

To connect Pygo to Arm/β‐catenin it takes a Lgs. This bridge protein, and its two vertebrate homologs BCL9 and BCL9L (referred to as BCL9/9L; the name derives from their causative role in B‐cell lymphoma, Willis et al., 1998), were thought to have the sole function of tethering the NPF‐containing, NHD transactivation domain of Pygo to the β‐catenin‐dependent complex. Evidence arguing in favor of this were compelling: a Lgs protein fragment that only included the regions encoding for the HD1 and HD2 domains (that bind Pygo and β‐catenin, respectively) could rescue the segment polarity phenotype caused by full *lgs* loss (Kramps et al., 2002). Other regions of Lgs, such as the additional HD3 domain, seemed dispensable (Hoffmans, Städeli, & Basler, 2005; Kessler et al., 2009; Kramps et al., 2002; Städeli & Basler, 2005). As we will explore below, only recent work uncovered a function for the HD3 domain of Lgs/BCL9. As its happens, two “forgotten” domains, the HD3 of BCL9 and the NPF‐motif of Pygo, might be the main characters pulling the strings of the multiprotein Wnt transcriptional complex present at the WREs (van Tienen et al., 2017).

### 4.7 Pygo reads the histone code

An interesting feature of Pygo is its affinity to select post‐translational modifications of the histone proteins. Structural and functional studies from the Bienz group identified a particular affinity of Pygo for di‐ or tri‐methylated lysines of the histone 3 tail (H3K4me2/3) (Fiedler et al., 2008). These chromatin marks are known to be present at actively transcribed regions and “open” promoters (Calo & Wysocka, 2013), suggesting an important role for Pygo as histone “code reader” at Wnt target loci. Additional crystallographic data showed that the PHD contacts the HD1 domain of BCL9/9L via a pocket located on the opposite side in respect to the H3K4me2/3‐binding residues, allowing Pygo to interact simultaneously with H3K4me2/3 and Lgs/BCL9 (Fiedler et al., 2008). Specifically, the binding to Lgs/BCL9‐HD1 induced allosteric remodeling of the Pygo‐PHD that conferred enhanced affinity for H3K4me2/3, reinforcing the notion that Pygo might act as code reader in a Lgs/BCL9‐HD1‐dependent manner (Miller, Rutherford, Johnson, Fiedler, & Bienz, 2010). While the structural observations were rock‐solid, several lines of functional evidence thrusted doubts on the relevance of the Pygo‐H3K4me2/3 interaction. Mutation of one of the residues responsible for interplay with H3K4me2/3 does not lead to obvious abrogation of Wnt signaling outputs nor to dramatic phenotypic defects both in mouse and in flies (Cantù, Valenta, Hausmann, et al., 2013; Kessler et al., 2009). It is conceivable that the relevance of the Pygo‐H3K4me2/3 interaction varies depending on the tissue context, developmental stage, or even the species under investigation. For example, vertebrate PYGO proteins—as opposed to Drosophila Pygo—appear to have a much higher affinity for H3K4me2/3 due to a single amino‐acid residue difference [a tryptophan (W) instead of a phenylalanine (F)] within the binding groove of the PHD (Kessler et al., 2009). If a naturally weaker interaction is fully functional in flies, this might explain why its strong reduction in the mouse does not perturb development (Cantù, Valenta, Hausmann, et al., 2013). It is interesting to notice that this function of PYGO might constitute an interesting exception from the two types of β‐catenin's partners that we described, Co‐Fs and TFs (see Section 2.4). While interacting with β‐catenin from “above”, PYGO also interacts with the chromatin “below” (Figure 2). This might render PYGO a crucial node for the positioning and the stabilization of the β‐catenin‐associated, DNA‐interacting TFs, and facilitate their interplay with the transactivation potential brought about to these loci by the other β‐catenin Co‐Fs. Importantly, while this issue remains controversial, future testing to inhibit this interaction might be needed for breast cancer cells where PYGO1/2 seem to play a crucial role in maintaining stemness traits and epithelial‐to‐mesenchymal transition (EMT;Andrews, Lake, Popadiuk, & Kao, 2007; Gu et al., 2009; Gu, Watanabe, Sun, Fallahi, & Dai, 2013). Recent work, in particular, highlights that the interaction with modified histones is important for driving de‐differentiation features of breast neoplastic lesions in the mouse, and its genetic abrogation induces differentiation pathways that attenuate primary tumor growth and metastasis formation (Saxena et al., 2020). This is exciting as the H3K4me2/3‐binding pocket can be effectively targeted by small molecules (Miller et al., 2014). The possibility that the “magic bullet” should hit a molecular interaction whose abrogation does not profoundly disturb homeostatic tissues surrounding the cancerous growth is the long‐standing ambition of cancer research.

### 4.8 Hyrax, a new link between β‐catenin and RNAPII activity

The discovery of new nuclear components of the Wnt pathway became one of the most pressing concerns in the field, and many of the genetic mutation screens carried out in model organisms reached saturation (Jenny & Basler, 2014). Complementary assays were designed in the attempt to identify novel factors. In the Basler group, an overexpression‐based screen in the wing pouch primordium uncovered a new gene, christened *hyrax*, capable to ameliorate the dampening of Wg/Wnt signaling caused by weak *lgs* alleles when overexpressed (Mosimann, Hausmann, & Basler, 2006). Drosophila Hyrax and its human ortholog Parafibromin were shown to directly bind to the C‐terminus of Arm/β‐catenin. Interestingly, Parafibromin/Hyrax share high sequence similarity to Cdc73p, a nuclear component of the *Saccharomyces cerevisiae* Polymerase‐associated factor 1 (PAF1) complex. Hyrax is an important addition to the equation describing how the β‐catenin complex translates to the RNAPII‐dependent execution of transcription. PAF1 is in fact a conserved multifunctional complex that regulates all stages of the RNAPII transcription cycle—initiation, elongation, and termination—across the animal kingdom (Van Oss, Cucinotta, & Arndt, 2017). Through interaction between Parafibromin/Hyrax and β‐catenin, PAF1 is converted from a general transcription co‐factor complex to a locus‐selective machinery whose activity only impacts certain genes. Of note, the eukaryotic PAF1 complex is involved in the regulation of H2B ubiquitylation. Specifically, PAF1 stimulates monoubiquitylation (ub1) of H2B K120 through interaction with other proteins (e.g., RAD6 and BRE1) to catalyze H2B‐ub1 formation. This mark inhibits chromatin compaction and, as we have seen before, is a prerequisite for di‐ and trimethylation of H3K79 and H3K4 by MLL and Dotcom (Van Oss et al., 2017). This is suggestive of an important concept: perhaps the requirement of PAF1 association to the β‐catenin transcriptional complex occurs before and is preparatory to that between β‐catenin and the MLL/Dotcom methyltransferase partners, implying a temporal order of recruitment of different enzymatic complexes during the phases of transcription.

One outstanding question remains. The β‐catenin ChIP signal is strictly observed onto specific genomic locations (i.e., WREs, Doumpas et al., 2019), while RNAPII spans the whole transcribed region (Fong, Saldi, Sheridan, Cortazar, & Bentley, 2017). By what mechanism does the β‐catenin associated PAF1 factors govern RNAPII movement, pausing and polyA‐site selection? Future work is needed to understand if PAF1 detaches from β‐catenin and moves along the transcribed region hand by hand with RNAPII, or if, in alternative, the β‐catenin‐PAF1 association is maintained to favor the formation of DNA loops displaying higher local concentration of the required factors, as it happens for example during the response mediated by nuclear hormones (Pezone et al., 2019).

### 4.9 An adult Wnt/β‐catenin enhanceosome

**FIGURE 4:**
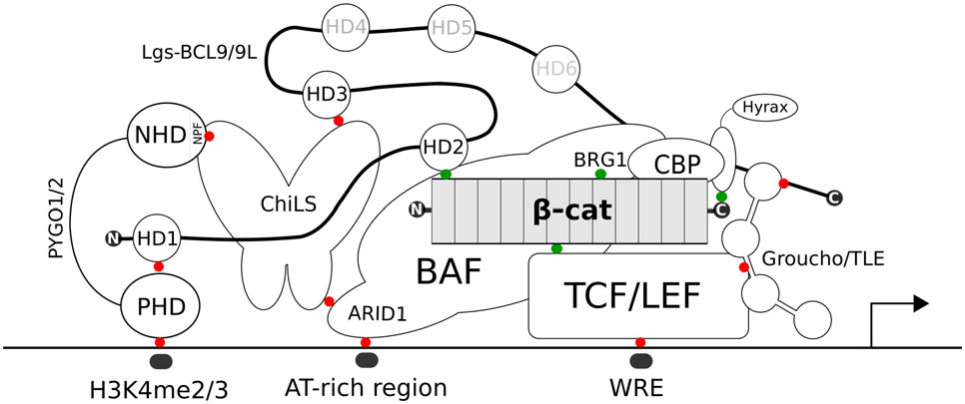
The Wnt/β‐catenin transcriptional complex, referred to as Wnt enhanceosome, as proposed by van Tienen and colleagues. Colored small balls indicate the physical interactions that glue each component within the complex. Green balls represent the interactions that only occur in the Wnt‐ON state, while all others (in red) appear to be constitutive. The long protein BCL9 or its paralogue BCL9L (BCL9/9L; orthologues of the Drosophila Lgs) run through the complex and keep it together via three fundamental interactions: with the PHD (Plant Homology Domain) of PYGO1/2 via the HD1 (Homology Domain 1); with the ChiLS complex via HD3; with TLE/Groucho via its C‐terminus (on the right side of the complex). The interaction between BCL9 and β‐catenin is mediated by the HD2 domain of BCL9/9L that contacts the N‐terminal portion of β‐catenin. Vertebrate BCL9 proteins possess additional homology domains (HD4‐6, in light gray), absent in the Drosophila Lgs, whose function is largely unknown. The NHD (N‐terminal Homology Domain) of PYGO1/2 contains the NPF tripeptide that interacts with the ChiLS complex. H3K4me2/3 stands for di‐ and trimethylated lysine 4 on the histone 3 tails, chromatin marks associated with active regulatory regions and promoters. WRE, Wnt Responsive Element

Sophisticated affinity purification‐based experiments followed by mass spectrometry from the Bienz laboratory recently brought about a complete and elaborated version of the nuclear transcriptional complex, sometimes referred to as Wnt enhanceosome (Gammons & Bienz, 2018; Lyou, Habowski, Chen, & Waterman, 2017; van Tienen et al., 2017) (Figure 4). Specifically, in two milestone studies, the three‐amino acid NPF motif of Pygo (Fiedler et al., 2015) and the so‐far neglected HD3 domain of BCL9 (van Tienen et al., 2017) were shown to mediate the cohesion among many proteins, including the usual suspects (e.g., TCF/LEF, Groucho/TLE, Osa/ARID1, and BRG1 of the BAF (SWI/SNF) complex) with additional ones, among which a new complex referred to as ChiLS. The ChiLS complex contains the LIM‐domain binding (LDB)/Chip factor, the single‐stranded DNA‐binding protein (SSDP), and a number of LIM‐domain proteins, such as LMO3/Beadex and LHX2/Apterous (Fiedler et al., 2015). Clonal analyses in Drosophila wing discs showed that LDB/Chip and SSDB are relevant to control the expression of Wnt target genes. Crystallography‐based systematic surface scanning by structure‐designed mutations showed that ChiLS adopts a rotationally symmetrical SSDP_2_‐LDB_2_‐SSDP_2_ architecture exposing two NPF binding pockets, whose integrity is required for Pygo binding (Renko et al., 2019). This kind of structural insights, in our opinion, will be the fundamental ingredient to reconstruct a clearer 3D model or the dynamics of the β‐catenin complex, and show how proteins interactions and positioning within the complex might underlie the integration of Wnt signals with the action of lineage‐specific factors.

In another set of experiments, co‐immunoprecipitation and in vitro binding assays based on nuclear magnetic resonance (NMR) revealed that also BCL9 associates directly to ChiLS via the HD3 domain, while simultaneously connecting with TLE/Groucho factors via the C‐terminus. This emphasizes the central role of Lgs/BCL9 as a core component of the complex in providing a scaffolding function, both in Wnt‐ON and Wnt‐OFF states. According to this model, Lgs/BCL9 would facilitate the complex assembly via three functionally separate interactions: with Pygo, ChiLS, and Groucho/TLE. These interactions precede the appearance of WNT signals, and a BCL9 conformational rearrangement accommodates the HD2‐dependent recruitment of β‐catenin to start transcriptional activation (van Tienen et al., 2017). Of note, LDB/Chip is known to mediate interactions with different tissue‐specific factors, and different LMO proteins might compete for its binding (Bronstein et al., 2010). As we will speculate later, the connection between LDB/Chip and β‐catenin might constitute a new toolbox to increase the promiscuity of the Wnt/β‐catenin‐dependent transcriptional complex.

## 5 HOW TO ACHIEVE TISSUE‐SPECIFIC TRANSCRIPTION: A COMPLEXITY WE DO NOT FULLY UNDERSTAND

### 5.1 Tissue‐specific β‐catenin co‐factors

Very early evidence suggested that the Wnt/β‐catenin transcriptional complex might vary depending on the target gene or the tissue‐context. In a genetic screen performed in Drosophila, the β‐catenin C‐terminus was caught binding the zinc finger protein Teashirt (Tsh), a segment‐specific homeotic TF (Gallet et al., 1998). This is interesting, as Tsh is a trunk‐specific modulator of cell fate and is exclusively expressed in the Drosophila embryonic trunk segments where it is required for segment identity in cooperation with other HOX proteins. Of note, Tsh is not expressed in the head and in the tail. The Tsh‐Arm interaction could therefore explain the transactivation potential of Arm/β‐catenin only in trunk cells, but not in other structures where Tsh is not expressed—but Arm/β‐catenin required. As early as 1999, the authors speculated that “the transactivating domain of Arm could bind several different and localized factors allowing tissue‐specific output” (Gallet et al., 1999). What could explain the transactivation potential of Arm/β‐catenin C‐terminus in the fly embryo rostral and caudal extremities?

Another instructive example, uncovered in an independent Drosophila genetic screen for Wg/Wnt signaling components, is that of the Centromere Binding Protein B (CENPB)‐type called Jerky (Earthbound1/Edb1 in flies; Benchabane et al., 2011). Edb1 loss could revert/suppress the apoptosis in retinal cells caused by mutations in *apc1* (these mutations constitutively activate the pathway). Ebd1 and its human orthologue Jerky share sequence similarity and function, and both interact directly with Arm/β‐catenin in a ternary complex with TCF. The problem is that, in contrast with the ubiquitous expression of other core pathway components, Ebd1 is only expressed in a restricted subset of Wg/Wnt responsive cells, including primarily myocytes, neurons, and glial cells, but is notably absent in ectoderm‐derived epithelia which are highly Wnt responsive (Benchabane et al., 2011). It follows that Edb1 must be relevant for only a limited number of signaling responses and be dispensable for β‐catenin action in ectoderm‐derived tissues. The vertebrate homolog Jerky is functionally equivalent to Edb1, as it rescues *ebd1* mutations when expressed in flies. Jerky also promotes Wnt signaling in human CRC cells, where it stabilizes the β‐catenin–TCF complex and facilitates recruitment of β‐catenin to the chromatin (Benchabane et al., 2011). This unequivocally suggests that also in vertebrates—and as a consequence in human cancers—we should expect to identify differences in the composition of the nuclear β‐catenin complex based on the presence or absence of tissue‐restricted players– like Edb1/Jerky or Tsh—that facilitate cell‐specific Wnt signaling responses by modulating β‐catenin/TCF activity.

### 5.2 Tissue responsiveness of Wnt target genes

Classical Wnt target genes contain WREs within their promoter or regulatory regions, and each WRE includes characterized TCF/LEF HMG consensus motif sequences (Yochum, 2011). It follows that an effective way of constructing a transcriptional Wnt reporter is to include tandem repeats of TCF/LEF HMG consensus motifs within a basal promoter (Maretto et al., 2003). Surprisingly, however, different enhancers in vivo display considerable variability in affinity for TCF/LEF, and this has been proposed to account for tissue‐specific expression of target genes (Chang, Chang, Barolo, et al., 2008; Chang, Chang, Gangopadhyay, et al., 2008; Lee & Frasch, 2000). While WREs might contain multiple clusters of TCF target sites, work from the Cadigan lab uncovered that Pan/TCF preferentially binds at specific TCF motifs among the many present, as it happens within the locus of the *nkd* target gene (Parker et al., 2008). These sites of preferential association contained a TCF‐Helper site consisting of seven nucleotides (GCCGCCA) in addition to the classical HMG binding motif (Chang, Chang, Barolo, et al., 2008). The Helper sites greatly increased the transcriptional activation in response to Wg/Wnt signaling, and was identified within the WRE of several other target genes (Chang, Chang, Barolo, et al., 2008). Mechanistically, the TCF‐Helper stretch is contacted by a C‐clamp domain present in certain isoforms of TCF/LEF (Chang, Chang, Gangopadhyay, et al., 2008; Hoverter, Ting, Sundaresh, Baldi, & Waterman, 2012). The Helper/C‐clamp interaction confers an additional layer of complexity to target gene regulation. A Helper site closely spaced to an HMG motif increased both binding and transcription from that locus. However, some of the four possible orientations of the Helper site in respect to the HMG motif conferred higher binding affinity for Pan/TCF when tested in gelshift competition assays (Archbold, Broussard, Chang, & Cadigan, 2014). It is important to specify that the chemical affinity between a TF with its cognate motif might not be linearly correlated with their function in vivo (Farley et al., 2015) and, consistently, all the four combinations of possible HMG/Helper orientations enhanced transcription to some degree (Archbold et al., 2014). The most interesting observation, in our opinion, is that when tested in Drosophila embryos different orientations of these binding motifs lead to flexible tissue‐specific enhancement when compared to HMG sites only. Some orientations increased reporter expression in wing imaginal discs, others in the eye/antennal discs, and even in non‐imaginal tissues such as the larval epidermis (Archbold et al., 2014). This series of experiments elegantly showed that even the DNA architecture might generate a repertoire of tissue‐specific responses to Wg/WNT signals. Perhaps, uneven expression of certain Pan/TCF isoforms having higher affinity for some combinations of Helper/HMG motifs cause target genes to vary depending on the tissue. In alternative, the C‐clamp/Helper site and its “optimal” placement increases the affinity for TCF/LEF thereby augmenting the sensitivity to respond to the Wg/Wnt morphogen. In this model, a lower local concentration of Wg/WNT would be sufficient to activate low‐sensitivity targets that are normally expressed only in cells proximal to the source of the Wg/WNT morphogen (Restrepo & Basler, 2011). This is compatible with a view in which tissue‐specificity of target gene expression is achieved by a combination of affinity for regulatory sequences and signal abundance. Evidence for this exists also in the mouse. An hypomorphic allelic series of the *Apc* gene demonstrated that each tissue during mouse development or in pathological homeostasis is differentially sensitive to thresholds of aberrant WNT‐dependent transcriptional activation: weaker or stronger *Apc* alleles disrupted cell function in a tissue‐dependent fashion (Buchert et al., 2010). According to this model, it is not the combination of proteins involved in the transcription, but a “just‐right” amount of signaling that would support the correct gene expression pattern or that could predict tissue transformation in WNT‐induced malignancies (Albuquerque et al., 2002).

### 5.3 The Pleiotropism of BCL9 and PYGO

**FIGURE 5:**
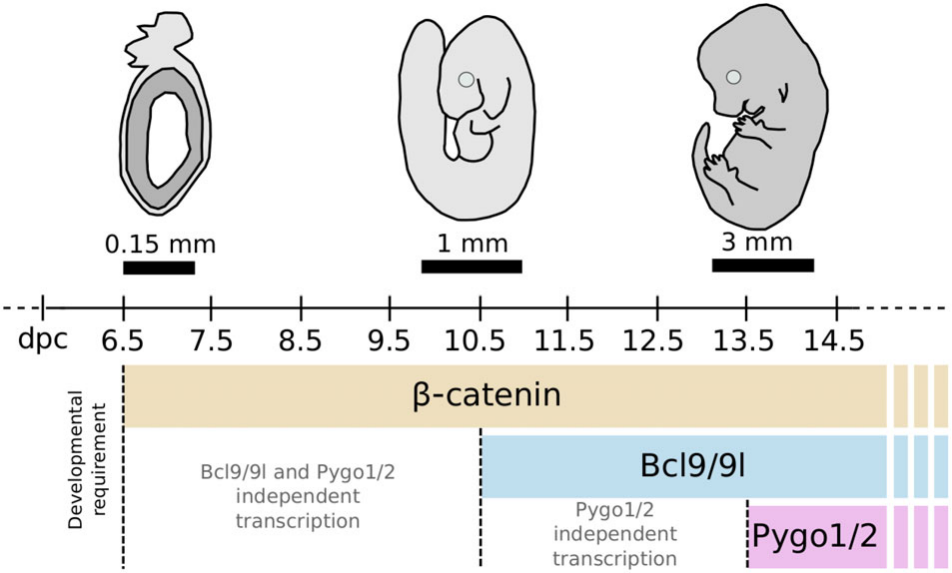
Developmental requirement of β‐catenin, BCL9/9L and PYGO1/2 as deduced from the stage in which lethality is induced by mutations in their genes during murine development. While *Ctnnb1* (encoding for β‐catenin) mutant embryos die during gastrulation at ca. 6.5 days post coitum (dpc), the lethality induced by mutations in *Bcl9/9l* or *Pygo1/2* occurs later, at ca. 10.5 and 13.5 dpc, respectively. This proves that β‐catenin can sustain the Wnt‐dependent transcription in the absence of the BCL9‐PYGO cooperation until 10.5 dpc, a stage in which the body axes have been established and organogenesis initiated. Analogously, *Pygo1/2* mutants die after 13.5 dpc, emphasizing that there must exist processes for which BCL9/9l are required but PYGO1/2 are not. Note that the lethality induced at 10.5 dpc by mutations in *Bcl9/9l* does not imply that all β‐catenin outputs must require BCL9/9L from this moment of development onward. It is in principle possible that only a single process required for survival is affected by loss of these genes

Upon their discovery, Pygo and Lgs/BCL9 immediately emerged as the ultimate activators of the Wg/WNT‐dependent transcription: loss of their function in Drosophila caused a complete loss of Wg outputs in all the contexts considered (Belenkaya et al., 2002; Kramps et al., 2002; Parker et al., 2002; Thompson et al., 2002). Hence, they were considered necessary for Arm/β‐catenin transcription. As mutations in *pygo* and *lgs* genes, in Drosophila, would coincide with loss‐of‐function of *wg* or *arm* but did not affect other processes or pathways, Lgs and Pygo were also defined as dedicated partners to β‐catenin (Hoffmans et al., 2005; Townsley, Thompson, et al., 2004; see also Sections 4.6 and 4.7). The reputation as loyal companions of β‐catenin changed when their role was investigated in the mouse. The results obtained by loss‐of‐function studies of *Pygo1/2* and *Bcl9/9l* were by and large “disappointing.” For instance, while the absence of β‐catenin induced developmental arrest during gastrulation at 6.5 dpc (Haegel et al., 1995) and this is entirely due to its signaling function (Valenta et al., 2011), compound mutations of the two *Pygo1* and *Pygo2* paralogs led to lethality between 13.5 dpc and birth (Cantù, Valenta, Hausmann, et al., 2013; Li, Rheaume, et al., 2007; Schwab et al., 2007) (Figure 5). Throughout different developmental stages, *Pygo1/2* loss affects some but not all WNT‐requiring tissues, and surprisingly it does not cause any malformation of the intestine whose renewal is highly dependent on Wnt signaling (Li, Rheaume, et al., 2007). Hence, also PYGO proteins must contribute to β‐catenin transcription in a tissue‐specific manner.

The two *lgs* orthologs, *Bcl9* and *Bcl9l*, performed only slightly better. Their compound loss‐of‐function causes lethality at ca. 10.5 dpc (Cantù et al., 2014), pinpointing that they are required before *Pygo1/2* during development. It is interesting to note that this observation leaves unexplained ca. 4 days of organogenesis between gastrulation and 10.5 dpc in which β‐catenin can regulate transcription in a manner that is completely independent of BCL9/9L (Figure 5). It seems however that neither PYGO1/2 nor BCL9/9L can fully explain the broad consequences of abrogating Wnt/β‐catenin dependent transcription during early development, or in mESC.

Sophisticated deletion experiments in mouse genetic models proved that the full tripartite β‐catenin/BCL9/PYGO “chain of adaptors” seems only active in specific cell types, such as the ectoderm‐derived migratory cardiac neural crest cells and mesodermal cardiomyocyte progenitors (Cantù et al., 2018). Another observation emerging from this study demands closer inspection. ChIP‐seq assays in vivo showed that the β‐catenin‐PYGO2 genomic targets in cardiac precursor cells are prominently genes involved in heart development (Cantù et al., 2018). These are by definition tissue‐specific physical targets, and are almost certainly not β‐catenin targets in other tissues where also β‐catenin regulates different cell fate decisions, such as, for example, cortical neurons (Draganova et al., 2014). In our opinion, this urges a redefinition of what we consider as “canonical” Wnt target genes. It has been suggested that PYGO, by constitutively sitting on the DNA, favors Arm/β‐catenin nuclear import via the Lgs/BCL9 tether (de la Roche & Bienz, 2007; Townsley, Cliffe, et al., 2004). Perhaps PYGO's function is even more precise: to drive Arm/β‐catenin in the nucleus and position it on the correct genomic target based on the right combination of chromatin marks. It will be interesting to test if the β‐catenin genome‐wide binding pattern is affected (and if so, in what tissues) in *Pygo* or *Bcl9* mutant cells.

But what additional co‐factors or “environmental” constraints could drive β‐catenin to the right genomic regions in other tissues where PYGO and BCL9 proteins are not relevant? As we discussed before, a possibility consists in the physical interaction between TCF/β‐catenin and tissue‐specific TFs, that could position the whole complex to novel regulatory regions based on the recognition of the cognate consensus motifs present in the proximity of tissue‐specific enhancers. In alternative, the pre‐existing chromatin conformation and its accessibility might dictate where TCF/LEF and β‐catenin can be recruited in the first place (Box 1). Several are the possible explanations: they do not necessarily exclude each other, and only new molecular analyses will be able to discriminate between them.

Concerning the role of PYGO and BCL9 proteins in Wnt/β‐catenin signaling, it gets even worse: PYGO and BCL9 are not only *non‐essential* for all β‐catenin outputs, but they are also *non‐dedicated*. PYGO and BCL9 proteins have in fact evolved β‐catenin independent functions. PYGO2—but not PYGO1—is required during spermiogenesis in a manner that is independent of Wnt signaling (Cantù, Valenta, Hausmann, et al., 2013; Nair et al., 2008). Furthermore, the PYGO‐BCL9 duet participates during eye lens formation in a PAX6‐dependent genetic circuit without involving β‐catenin (Cantù et al., 2014). BCL9 and PYGO even possess β‐catenin‐independent nontranscriptional functions in the oral epithelium‐derived enamel‐secreting ameloblasts, where they display a predominant cytosolic localization, and participate in the secretion of enamel components. This function likely evolved recently, concomitantly with the relatively new (evolutionarily speaking) enamel‐type extracellular matrix (Cantù et al., 2017). But the cytoplasmic localization might constitute a more general feature. It was recently shown that BCL9 moonlight in the cytoplasm also in mitotic Hela cells, where it regulates the β‐catenin destruction complex and competes with clathrin for binding the LRP6/AXIN1 complex, thus sustaining mitotic Wnt signaling (Chen et al., 2018).

The example of the oral epithelium is enlightening for an important reason: it emphasizes the need for tissue‐specific co‐factors for β‐catenin. The formation of teeth, which originate from this epithelium, is indeed dependent on the presence of β‐catenin (Liu et al., 2008). When *Ctnnb1* is conditionally deleted using a Keratin14 promoter‐driven Cre recombinase expressed in this tissue, or when only the signaling function of β‐catenin is perturbed, tooth development is arrested at its onset (Cantù et al., 2017; Liu et al., 2008). Hence, β‐catenin dependent transcription is needed for teeth development. On the other hand, compound conditional knockout of *Bcl9*/*9l* or of *Pygo1/2* using the same Cre‐driver leaves tooth development unaffected. Differently from cardiac neural crest, here β‐catenin regulates the transcription in the absence of the BCL9‐PYGO module (Cantù et al., 2017). Future work is needed to address what are the other yet‐unknown co‐factors that assemble the Wnt transcriptional machinery in the absence of the crucial scaffolding function of Lgs/BCL9 (van Tienen et al., 2017).

### 5.4 RUNX or not RUNX

The Runt‐related TF RUNX comes and goes from the β‐catenin transcriptional complex. In the cardinal work from Fiedler and colleagues, ChiLS was found in direct connection to the β‐catenin transcriptional complex via the NPF motif of Pygo (Fiedler et al., 2015). Interestingly, ChiLS gets also in contact with the NPF tripeptide present in other proteins, including RUNX, implying physical, and possibly functional association (Fiedler et al., 2015). Notably, recent experiments using the BioID proximity labeling technology could not identify any RUNX proteins participating in the β‐catenin transcriptional complex in HEK293T cells (van Tienen et al., 2017). Perhaps the epithelial HEK293T cells do not express any RUNX factor. Alternatively, RUNX might act in a “hit‐and‐run” style, dodging easy detection. Or, perhaps, RUNX proteins pioneer chromatin opening in the phases preceding the transcriptional initiation (Mevel, Draper, Lie‐A‐Ling, Kouskoff, & Lacaud, 2019), thereby assisting the subsequent assembly of other Wnt/β‐catenin co‐factors. But it is also possible that the RUNX proteins underscore a fundamental difference between tissues, and their participation occurs during early embryonic development or in uncommitted progenitors but not in differentiated epithelial cells, such as HEK293T (van Tienen et al., 2017).

The RUNX factors and Wnt signaling are friends going back a long way (Sweeney, Cameron, & Blyth, 2020). There are three RUNX paralogues in vertebrates, (RUNX1, 2, and 3) and they orchestrate a broad spectrum of processes such as lineage commitment in the hematopoietic system, a nontranscriptional role in DNA‐repair, and an activity as pioneer factors that facilitate opening of condensed chromatin (reviewed in Mevel et al., 2019). Interplay between Wnt and RUNX1 had been previously reported by evidence that LEF1 enhanced RUNX1 binding to chromatin, with the consequence of potentiating the transcriptional activity driven by the T‐cell receptor alpha (TCRα) enhancer (Mayall, Sheridan, Montminy, & Jones, 1997). RUNX1 is also known as AML1, for it is frequently involved in acute myeloid leukemia‐causing translocations (Licht, 2001). Of note, Wnt signaling activation facilitated translocation events in leukemic cells leading to the oncogenic activity of the AML‐ETO chimera (Licht, 2001). This is interesting, as it unearths the existence of a novel oncogenic paradigm of hemopoietic malignancies driven by the Wnt/β‐catenin signaling in concert with the RUNX1 pro‐leukemic activity (Sweeney et al., 2020).

The paralog RUNX2 behaves differently. RUNX2 protein is repressed by LEF1 via direct interaction through the HMG domain, and constitutively active β‐catenin enhances this LEF1‐mediated suppression (Kahler & Westendorf, 2003). This negative control is reciprocal, as enforced expression of RUNX2 in osteoblasts reduces β‐catenin levels and inhibits its transcriptional activity to fine‐tune bone resorption (Haxaire, Haÿ, & Geoffroy, 2016). In addition to a reciprocally negative regulation, β‐catenin and RUNX2 can cooperate for direct regulation of some genes, such as *FGF18* (Reinhold & Naski, 2007) and *Osteocalcin* (Tang et al., 2009), whose promoters display adjacent TCF/LEF and RUNX binding motifs.

The third sibling, RUNX3, can form a ternary complex with β‐catenin and TCF7L2, and attenuates their DNA‐binding affinity ultimately acting as a tumor suppressor in CRC (Ito et al., 2008).

As it is emerging in the context of their cooperation with Wnt signaling, RUNX proteins are so eclectic as to act both as oncoproteins or tumor suppressors. The “tango” between this family of TFs and the Wnt/β‐Catenin pathway is thoroughly examined in Sweeney et al., 2020.

Evidently, the complex interplay between RUNX and the Wnt dependent transcription demands further scrutiny.

### 5.5 ChiLS: A portal to new dimensions

As previously mentioned, ChiLS includes the LIM‐domain binding LDB (Chip in Drosophila), SSDP, and the LIM‐domain proteins LMO3 and LHX2 (Beadex and Apterous in flies, respectively; Fiedler et al., 2015; van Tienen et al., 2017). The participation of ChiLS in Wnt signaling was an important discovery that, as we speculate, might be a crossroad for the access of promiscuous protein complexes cooperating with β‐catenin. In addition to RUNX proteins, in fact, the ChILS complex allows additional combinations of co‐factors. LDB is a highly conserved, ubiquitous nuclear multi‐adaptor protein that mediates the interaction between different classes of TFs and regulators (we refer to the neat review by Matthews & Visvader, 2003 on this subject). Notably, one of the first interactors of LDB identified was precisely Apterous, and this connection was shown to be required for the formation of the wing margin in flies, compatibly with a potential role in Wg/Wnt signaling (Milán, Diaz‐Benjumea, & Cohen, 1998). The interest in LDB/Chip lies in the fact that concomitant protein–protein interaction with non‐LIM transcription factors increases the complexity of its downstream output. Specifically, LDB/Chip forms an alternative complex with the zinc‐finger family of GATA TFs, important in a plethora of processes from erythropoiesis (Bosè et al., 2006) to cardiac progenitors specification (Sachinidis et al., 2003). In alternative, the beta helix–loop–helix (BHLH) TFs Achete and Scute team up with LDB/Chip for the formation of the thoracic macrochaete (sensory bristles) differentiation (Ramain et al., 2000). Of note, the vertebrate ortholog Achaete‐scute like 2 (*ASCL2*) is a Wnt target gene in intestinal neoplasia (Jubb et al., 2006), where it acts as master regulator of intestinal stem cells identity. Recent work from the Clevers lab is consistent with a celltype‐specific cooperation between β‐catenin and ASCL2. In this work, it is shown that ASCL2 collaborates with β‐catenin‐TCF/LEF to activate the Wnt target genes responsible for maintaining the stem cell state. Among the relevant targets, *Ascl2* itself is controlled in a direct autoactivatory loop, enhancing the distinction between ON and OFF states downstream of WNT/R‐Spondin signaling (Schuijers et al., 2015). In this model, ASCL2 works as a transcriptional switch that is both Wnt responsive and Wnt dependent and contributes in defining stem cell identity. In Drosophila, LDB/Chip connects the GATA TF Pannier with Achaete/Scute proteins to allow enhancer–promoter interactions in the activation of the proneural genes, while Apterous antagonizes Pannier function by binding competition. The accurate stoichiometry relationship between these factors acts as a switch for the activation or inhibition of proneural compartments (Ramain et al., 2000). It is possible that a similarly subtle balance regulates the Wnt outputs also in the murine intestinal epithelium, where ASCL2 is specifically expressed in the stem cell compartment and sustains self‐renewal (and its own expression), while decreasing ASCL2 local concentrations might allow other LIM‐domain TF (e.g., LHX2/Apterous) to take over within the β‐catenin enhanceosome in differentiated epithelial cells. It seems that SSDP is required in both these LDB/Chip dependent complexes (van Meyel, Thomas, & Agulnick, 2003). Also this is consistent with the finding from the Bienz laboratory, as *ssdp* mutant clones within the Drosophila wing imaginal discs produce wing defects that phenocopy *pygo* mutant clones (Fiedler et al., 2015; Van Meyel et al., 1999).

In summary, these compelling observations underscore how the nuclear adaptor LDB/Chip is a prominent developmental regulator capable of forming tissue‐specific transcriptional complexes. Its cooperation with the β‐catenin complex makes it a valuable ally to increase the possible intricacies of gene regulation downstream of WNT signals.

### 5.6 Permutational Chaos

**FIGURE 6:**
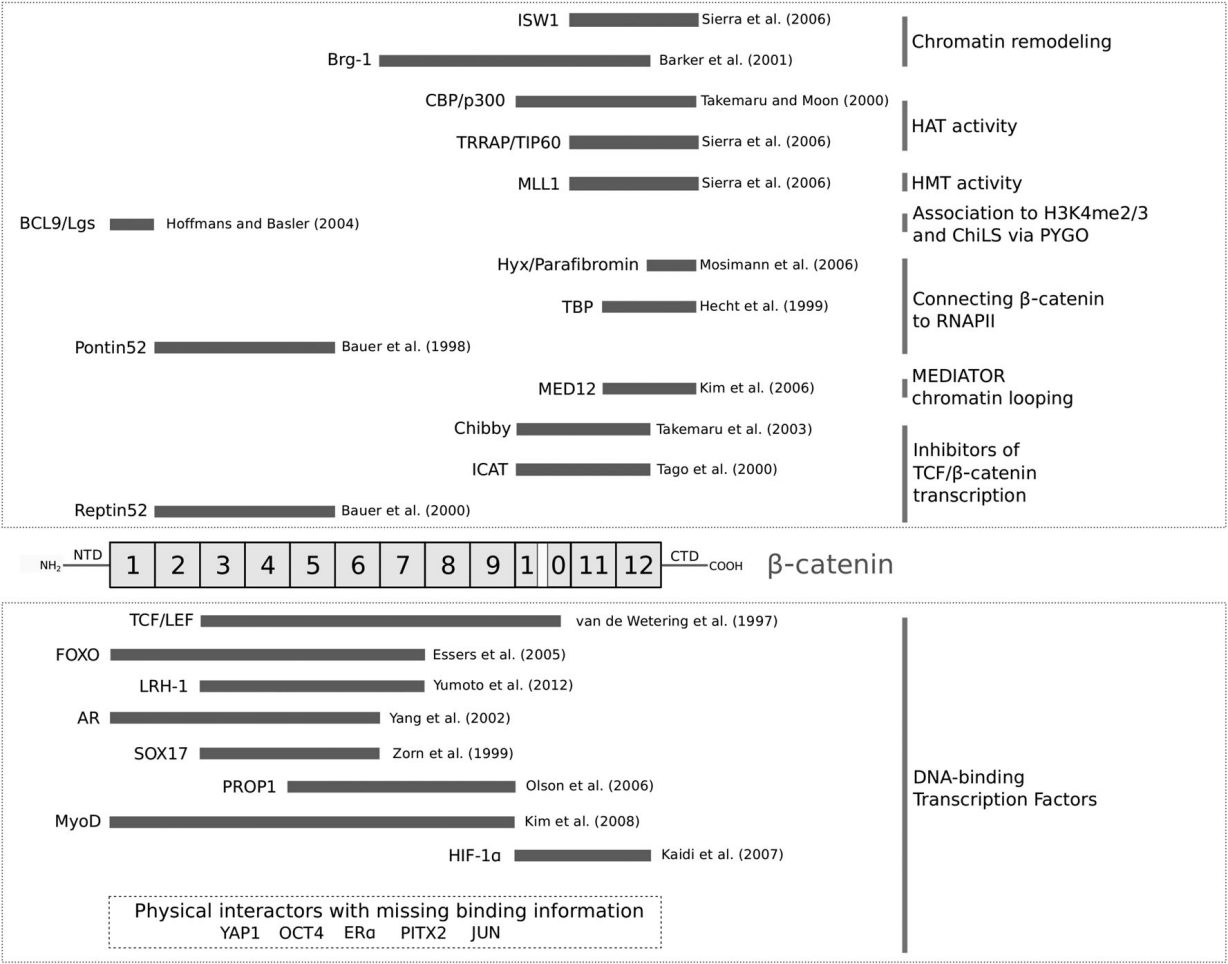
β‐catenin is a docking station for a plethora of nuclear proteins. β‐catenin is represented in the center of the figure, and its partners are listed below (transcription factors) and above (transcriptional regulators). For each interacting protein, a gray bar spans its binding surface along the corresponding domains of β‐catenin. On the right of each interactor, the relevant reference describing binding information. The main activity of each interactor (or group of interactors) is pinpointed on the right of the figure. The numbers within β‐catenin list the armadillo repeats. The white bar in armadillo repeat 10 indicates the 22 amino acid residues insertion causing the deviation from the armadillo repeat structure (Huber, Nelson, & Weis, 1997). For sake of simplicity, this is not represented in the other figures of this article. CTD, C‐terminal domain. H3K4m2/3, di‐ and tri‐methylated lysine 4 on histone 3 tail; HAT, histone acetyltransferase; HMT, histone methyl transferase; NTD, N‐terminal domain; RNAPII, RNA polymerase II

We have considered two sets of players managed by β‐catenin to fulfill its nuclear function. On one hand, in Section 3 we listed the cooperative and alternative TFs that solve the question of specificity: how β‐catenin finds its targets on the vast DNA informational space. On the other, in Section 4 we have described a deluge of partners and chromatin‐modifying complexes recruited by different armadillo repeats to prepare the chromatin ground and establish contacts with RNAPII. Importantly, β‐catenin permits simultaneous scaffolding of TFs on one side and of co‐activators on the other (Figure 2), as these two categories of interactors do not compete for binding. For instance, TCF/LEF might clash with FOXO factors, but they will not hinder binding of BCL9, allowing the formation of the tripartite complex TCF‐β‐catenin‐BCL9 (Kramps et al., 2002). As each of the two categories of β‐catenin partners comprises several members, an intriguing theoretical possibility emerges. If all members of one category do not compete for binding with members of the other, it is then conceivable that β‐catenin is the fulcrum of a combinatorial system of transcriptional complexes allowing all the possible permutations (Figure 6; see also graphical abstract and Figure 2). Such a system would imply the existence of various combinations of β‐catenin interacting factors in different tissues. A number of theoretical but testable predictions are raised by this conjecture, and some experimental instances are already corroborative of it. In the context where β‐catenin participates in SOX17‐mediated transcriptional regulation, the transactivation motif at the C‐terminus of β‐catenin is required for a subset of SOX17 targets, such as *Hnf1β* and *Foxa2*, but not for others, like *Foxa1*. The basis of this difference must rely on the recruitment of differential co‐factors. Perhaps β‐catenin recruits the co‐activators CBP/p300 and/or Hyrax/Parafibromin specifically on *Hnf1β* and *Foxa2*, but not on *Foxa1* promoter (Sinner et al., 2004). The combination SOX17/β‐catenin/CBP might regulate *Hnf1β*, while a rearranged SOX17/β‐catenin/BCL9 could be found at the *Foxa1* locus. In an analogous example, the pituitary homeodomain factor PROP1 appears to benefit of this functional pliability of β‐catenin, which recruits HDAC/TLE complexes to repress certain PROP1 target genes and CBP to activate others in a binary manner within the same cell (Figure 3; Olson et al., 2006). A third example comes from our recent work unraveling the T‐box containing TF TBX3 as part of the Wnt‐dependent transcriptional complex (Zimmerli et al., 2020). The participation of TBX3 is strictly dependent on the presence of BCL9 and occurs on classical WREs. The BCL9‐dependent TBX3 genomic occupancy of WREs confirms this model (Zimmerli et al., 2020). It is tantalizing to speculate that the previously mentioned interplay with TBX5 (Figure 3; Rosenbluh et al., 2012) and that with TBX3 are simply two cases of a more general networking between Wnt/β‐catenin and the TBX TFs. The β‐catenin/TBX teamwork might be extremely ancient, and ontogenically relevant by starting with the forefather of all the T‐box proteins, T/Brachyury, a Wnt/β‐catenin target required for mesoderm induction during gastrulation (Arnold et al., 2000; Papaioannou, 2014).

Overall, we conjecture—and await the discovery of—a colorful array of promiscuous factors all of which physically dock cooperatively onto β‐catenin armadillo repeats. Should we expect to find BCL9/9L‐PYGO, CBP/p300, or Hyrax/Parafibromin to be functionally linked to those loci where FOXO4, MYOD1, or HIF1α drive β‐catenin onto alternative regulatory sequences? The beauty of this hypothesis is that it is, in principle, verifiable (or falsifiable) with current technology.

While this remains a theoretical proposition, β‐catenin protein structure itself offered a first hint into its function as a hub of combinatorial and noncompetitive multiprotein complexes. A broad central region (from residues 141–664 in human β‐catenin) that consists of 12 imperfect Armadillo repeats forms a rigid scaffold required for the simultaneous anchoring of several interaction partners (Huber et al., 1997). The idea that evolution generated a multimeric scaffolding “pier” with several docking regions on two sides with similar moieties is persuasive. This would be a simple but very ingenious way of granting the assembly of alternating transcriptional complexes in distinct cells—or even on different loci within individual cells: an ideal recipe for combinatorial gene expression. This would not be even entirely new: classical TFs are known to exert a combinatorial control on gene expression by binding to multiple tissue‐specific players of the same or unrelated families in a combinatorial fashion by using different sets of interaction surfaces (Reményi, Schöler, & Wilmanns, 2004). This is the way of nature to produce varying transcriptional programs starting with a limited set of operating machineries.

### 5.7 Defining the Wnt/β catenin enhanceosome—A challenge for the field

In spite of this broad collection of knowledge consolidated in the last 30 years, a list of binding partners cannot be a sufficiently satisfactory explanation of how β‐catenin operates. Gene regulation is in fact achieved by a temporally coordinated and cooperative assembly of basal TFs and chromatin modifying enzymes to cis‐regulatory DNA elements, such as distant enhancers and promoters. The resulting nucleoprotein structure composed of DNA/chromatin, TFs and enzymatic activities is dynamic but also remarkably stable, and has been termed enhanceosome (Merika & Thanos, 2001). By definition, the description of the enhanceosome assembly requires details about the precise spacing between the binding sites of all the DNA‐contacting factors*—*ensuring that each constituent maintains simultaneous proximity to its companions and to the DNA *–*, as well as of the timing of their recruitment (Merika & Thanos, 2001).

When it comes to the nuclear action of β‐catenin, these strata of information are still poorly understood, and the real nature of the Wnt enhanceosome still eludes our grasp. In summary, we do not fully understand: (i) how different β‐catenin‐dependent factors that possess DNA‐binding capacity cooperate by contacting correctly spaced adjacent binding sites within the WRE; (ii) how chromatin modifying enzymes are called up in a temporal fashion to assist the access of RNAPII to promoters; and (iii) how nonubiquitous co‐factors and TFs contribute to the establishment of cell‐specific gene regulation.

A number of questions relevant to understand these points could be experimentally addressed by existing technology. For example, are all β‐catenin co‐factors simultaneously present on the WRE? If not, in what temporal/epistatic order are they brought together? What is the genomic distribution of all the TFs and co‐factors before the WNT stimulus triggers the pathway, and how is their rearrangement adjusted upon β‐catenin arrival? Is the Wnt/β‐catenin‐dependent transcriptional machinery equal in all cell types? Could its assembly even be different on each WRE within the same cell?

We are far from providing the answer to each of these questions. Nevertheless, we are confident that future work will address them. But this effort is much needed, and below we show why.

### 5.8 More work is needed—Do not leave anyone behind

**FIGURE 7:**
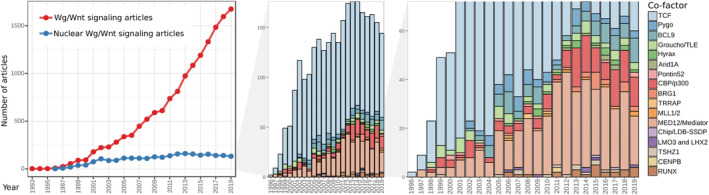
There is a steady and rapid increase in the number of published articles in the Wg/Wnt field (red curve, left panel). This is of no surprise considering the rapid exponential growth of the biomedical literature in every sector (Khare, Leaman, & Lu, 2014). What is surprising is the plateau phase reached by the subset of articles in the field that focus on nuclear components (blue curve, left panel). After their discovery as main transcription factors assisting β‐catenin in 1996, TCF/LEF have been mentioned in an exponentially growing number of articles for the subsequent decade (see the more detailed central panel). It appears however that the interest in nuclear TCF/β‐catenin co‐factors has not been growing in a manner similar to the rest of the field. The central and right charts represent the contribution of each of the β‐catenin co‐factors to the total number of nuclear Wg/Wnt signaling papers (each factor is listed in a color‐coded legend table on the right)

We interrogated the PubMed database for quantifying the scientific literature focusing on Wnt signaling. We used the R package RISmed (developed in 2017 and maintained by Stephanie Kovalchik) to scout all publications containing the term “Wg/Wnt” in the title and/or in the abstract. We also restricted the search to publications annotated by MEDLINE as “journal article” (e.g., not as “review”). It is unsurprising*—*but comforting*—*that this search produced a steeply rising curve displaying the annual increment of original articles in the field (Figure 7). We then wished to extract the studies directly investigating the mechanisms of nuclear Wnt signaling. We reasoned that to enrich for this category we should consider the articles that mention Pangolin or TCF or LEF (in addition to Wg/Wnt) within their title or abstract. When these terms were added, the resulting curve (which of course represents a fraction of the total Wg/Wnt articles) flattened in a prolonged plateau, especially when considering the last decade (Figure 7). We interpret the expanding gap between the total Wg/Wnt and the TCF/Wg/Wnt articles as a disproportional interest in different aspects of this cascade, with a deficit on the nuclear mechanisms of transcription. We rendered our search more detailed by asking how many of these papers look at the function of individual β‐catenin co‐factors discussed above. Only a considerably small fraction of the “nuclear Wnt signaling” articles is specifically focusing on at least one of these proteins (Figure 7, right panel). This is worrying, especially given the immeasurable relevance that the Wnt‐dependent transcription has for cell behavior during development and organismal pathophysiology (Section 6). This short paragraph has one goal: it is our appeal to bring again nuclear Wnt signaling under the spotlight.

## 6 WNT‐DRIVEN CANCERS: TURNING TO THE DARK SIDE

### 6.1 How could the empire be defeated? Difficulties in targeting oncogenic Wnt signaling

Wnt signaling is essentially a way of activating certain sets of genes in a cell. Among the genes typically turned on by the β‐catenin‐mediated transcription there are many that sustain cell cycle progression and cell division, such as *CCND1* (Shtutman et al., 1999; Tetsu & McCormick, 1999) and the proto‐oncogene *MYC* (He et al., 1998). For an up‐to‐date list of target genes the reader should consult the Wnt homepage regularly curated by the Nusse Lab, at https://web.stanford.edu/group/nusselab/cgi-bin/wnt/target_genes. The activation of these genes renders Wnt signaling a survival signal for many types of adult stem cells (Habib et al., 2013). Therefore, it is of no surprise that an overactive Wnt signaling is associated with neoplastic growth from many tissues. Many authors successfully summarized our knowledge connecting the Wnt pathway to cancer, and the attempts of the scientific community to translate it into a more clinical setting. Rather than being redundant with them, we wish to suggest a series of authoritative recent reviews on this subject: Flanagan, Vincan, & Phesse, 2017; Janovská & Bryja, 2017; Lyou et al., 2017; van Kappel & Maurice, 2017; Zimmerli, Hausmann, Cantù, & Basler, 2017.

Here, we wish to emphasize one aspect of this critical connection. We have described how Wnt signaling is mechanistically executed in different manners depending on the context, and how this elicits a varying gene expression response. Should we therefore expect that Wnt signaling bolsters carcinogenesis via different mechanisms? If this is the case, different Wnt‐driven cancers might require distinct interventions aimed at inhibiting the particular player(s) that are relevant in the corresponding context.

Well, there is evidence for this. Notably, available data appear to suggest that distinct tissues might be susceptible to particular cancer‐driving genetic perturbation of Wnt signaling components. While *APC* mutations are present in ca. 80% of CRC, mutations in *AXIN* is most prevalent in hepatocellular carcinomas, while activating mutations in *CTNNB1* have been found with high frequency in melanoma and thyroid tumors (Groden et al., 1991; Kahn, 2014; Mazzoni & Fearon, 2014; Powell et al., 1992). It appears that the Wnt pathway provides many possibilities for cancer cells to exploit normal developmental processes for their own unrestrained growth and proliferation. This heterogeneity in Wnt signaling activating mutations among different tumors certainly indicates a need to consider different treatment strategies in each specific case. To add to this complexity, while tumor initiation is often driven by activating Wnt signaling, there are also cases where maturation of a tumor into a fully malignant form is dependent on inhibition of β‐catenin‐dependent Wnt signaling, as it happens in melanoma (Chien et al., 2009). Even for the paradigmatic Wnt‐driven CRC, it has been proposed that while high TCF/Wnt activity sustains tumor initiation, aggressive metastatic CRCs establish a Wnt‐low but Hedgehog‐high gene expression signature, and inhibition of TCF activity is undesirable (Varnat et al., 2010).

This tremendous heterogeneity of tumors presenting variable Wnt signaling activity contributed to render the pathway a problematic target for therapeutic interventions. Nevertheless, much work has been dedicated toward finding a solution, ranging from blocking the secretion of Wnt ligands to inhibiting cell surface receptors like FZD and LRP. Particularly promising appears the pharmacological inhibition of the Wnt acyltransferase PORCN, required for lipid‐modification of WNT proteins that promote their secretion (Proffitt et al., 2013). Also this approach has its drawback, as use of such inhibitors is accompanied by loss of bone density that needs to be mitigated by treatment with drugs against osteoporotic conditions (Madan et al., 2018).

### 6.2 Hit the death star at its nuclear signaling core

One attractive approach is obviously that of targeting the nuclear components of the pathway. Interfering directly with the most downstream part of the cascade—such as inhibiting the TCF‐β‐catenin interaction—should in theory weaken all possible outputs of the aberrantly activated canonical Wnt signaling, irrespectively of the gene in which the culprit oncogenic mutation hit. Even in considering this approach, there are a number of obstacles that need to be overcome. To begin with, the “magic bullet” needs to be designed such that it solves the logistic problem of crossing both the plasma membrane and the nuclear envelope, navigating through legions of macromolecular complexes to finally find and disrupt only the specific protein–protein interaction of interest. If this is achieved, how could a drug reach this molecular resolution and simultaneously act in a tumor cell‐specific manner? Is it desirable to efficiently inhibit the TCF/β‐catenin interaction in tumor cells with the risk of affecting the surrounding healthy tissue? Considering that β‐catenin‐dependent Wnt signaling nurses tissue homeostasis and maintenance of the stem cell pool in many organs, any attempt to inhibit it should be carried out with caution (Kahn, 2014).

Another dilemma lies in how β‐catenin or TCF/LEF should be targeted. The binding affinity between β‐catenin and TCF/LEF is relatively high and sustained by a large chunk of the β‐catenin structure across seven armadillo repeats (Behrens et al., 1996). For chemical screenings aimed at developing small inhibitory molecules this is certainly a complicated case (Daniels, Eklof Spink, & Weis, 2001; Kahn, 2014; Wenqing Xu & Kimelman, 2007). This is rendered more troublesome by the convergence of binding modalities among positive and negative regulatory partners of β‐catenin. For example, β‐catenin associates with E‐cadherin as an important component of the *adherent junctions*, and the E‐cadherin interaction site on β‐catenin overlaps with that of TCF/LEF. Other proteins, such as APC, AXIN, and ICAT also contact a portion within the TCF/LEF interacting surface; interfering with this part of the β‐catenin molecule might therefore result in the undesired activation rather than inhibition of the pathway (Lyou et al., 2017).

Finally, an idiosyncratic worry derives from considering recent work on the promiscuity of β‐catenin. When the TCF/LEF‐β‐catenin interaction is abrogated, is not stabilized β‐catenin free to team up with other TFs and engage in alternative—and possibly detrimental—activities? For example, in the absence of TCF/LEF, β‐catenin sticks to FOXO TFs to activate a so‐called GHOST response (Doumpas et al., 2019), which includes the transcription of genes such as Matrix metalloproteases (MMP13) whose overexpression is a prognostic marker for CRC (Huang et al., 2010). Other cases mentioned above, such as the cooperation with LRH‐1 in inducing cell cycle progression (Botrugno et al., 2004), or the oncogenic activity with YAP1/TBX5 (Rosenbluh et al., 2012), might contribute to start a growing avalanche of unpredictable consequences.

Nevertheless, a number of promising alternative targets could be found in the thick of the multitude of nuclear β‐catenin co‐factors. Many screens have been performed with the aim of identifying pharmacologically useful inhibitors of the C‐ and N‐terminal β‐catenin co‐factors, even though only few have reached clinical trials as‐of‐yet. In SW480 CRC cells a screen of 5,000 molecules was performed to find inhibitors of the Wnt transcriptional reporter TOPflash. This identified ICG‐001 as the most potent candidate. This compound acts by shielding CBP from its interaction with β‐catenin (Emami et al., 2004). So much for its specificity: ICG‐001 blocked CBP but not its paralogue p300. ICG‐001 administration repressed the expression of Wnt targets *SURVIVIN* and *CCND1*, thereby pushing cell cycle withdrawal in the human CRC cell lines HCT116 and SW480. Recently, a second generation inhibitor of β‐catenin/CBP‐interaction has been developed, called PRI‐724, which to date successfully entered several clinical trials (Kimura et al., 2017).

Another interesting target is the cyclin‐dependent kinase 8 (CDK8), identified in a screening for oncogenes that modulate β‐catenin‐dependent transcription (Firestein et al., 2008). CDK8 encodes a member of the Mediator complex, and its expression potently vitalizes β‐catenin‐dependent transcription and transformation. As the genomic locus containing *CDK8* is amplified in CRC and several other cancers, it was deemed as a likely general targeting option. Moreover, a functional discrepancy between CDK8 downregulation or inhibition suggested that its activity in Wnt signaling is mediated by its kinase domain, an ideal enzymatic candidate for therapeutic inhibition. CDK8 promotes EMT of pancreatic cancer cells as a consequence of a K‐RAS > HIF‐1α upstream activation, and its activity correlated with increased nuclear β‐catenin (Wei Xu et al., 2015). Whether CDK8 acts in concert with β‐catenin, or it is activated by the coordinated action of HIF‐1a/β‐catenin remains to be determined. While the interest in CDK8 inhibition remains high also for several other pathological conditions, only a few inhibitors reached the testing in clinical trials to date (Xi et al., 2019).

### 6.3 A new hope

As previously suggested, the interest in the β‐catenin co‐factors BCL9 and PYGO dropped when it was shown that their loss in mouse models did not lead to the dramatic phenotypic defects expected for constitutive components of the pathway. But recent work brought back these proteins into the spotlight for their interest as appealing molecular targets against oncogenic Wnt signaling. Admittedly, BCL9 was originally discovered as causative genetic factor of B‐cell acute lymphoblastic leukemia, and thought to act as an oncogene at least in hematopoietic malignancies (Kramps et al., 2002; Mani et al., 2009; Willis et al., 1998). But the genomic region containing human *BCL9* (1q21) is amplified in several cancers (Beroukhim et al., 2010), and this correlates with poor clinical outcomes (Carrasco et al., 2006). In mouse model of CRC, BCL9 and its paralogue BCL9L promote both early stages of carcinogenesis (Brembeck et al., 2011), as well as late‐occurring traits associated with EMT and cancer metastasis (Brembeck et al., 2004; Deka et al., 2010; Mani et al., 2009). BCL9/9L are required for intestinal transformation induced by both loss of *Apc* or gain‐of‐function of *Ctnnb1*, and loss of epithelial *Bcl9/9l* dampens Wnt signaling transcription to a level that no longer permits cellular transformation (Gay et al., 2019). Interestingly, transcriptional downregulation of Wnt targets is lower than that caused by mutated *Ctnnb1* (Valenta et al., 2016). This supports the hypothesis that, in CRC cells, BCL9/9L enhances Wnt target gene transcription to the “just‐right” amount that allows carcinogenesis (Albuquerque et al., 2002; Gay et al., 2019) and their loss offers a perfect therapeutic window, for it causes the downregulation of gene expression just below a crucial threshold that impairs aggressive cancer behavior and regeneration but not normal homeostasis (Deka et al., 2010; Gay et al., 2019). In this context, BCL9/9L synergize with PYGO1/2, as the gene expression changes caused by their compound genetic deletion is broader than that provoked by recombination of *Bcl9/9l* or of *Pygo1/2* (Mieszczanek, van Tienen, Ibrahim, Winton, & Bienz, 2019). Intriguingly however, deletion of *Bcl9/9l* prolonged lifespan of mice carrying the *Apc* ^*1322T*^ mutation (which encodes a truncated but partially‐functional APC commonly found in CRC) significantly more than *Pygo1/2* genetic recombination (Mieszczanek et al., 2019), and PYGO1/2 also seem to be not particularly relevant in chemically induced colorectal carcinogenesis (Zimmerli et al., 2020). It is possible that other BCL9/9L interacting partners are more relevant in CRC, such as TBX3 (Zimmerli et al., 2020). The exact mechanisms of Wnt target gene activation in CRC by BCL9/9L are still elusive but are certainly worth investigating.

Regardless of the mode of action, when considering BCL9 as a therapeutic target in CRC, two earlier observations by Moor et al. (2015) are worth mentioning. First, genetic recombination of *Bcl9/9l* was induced by tamoxifen injection when established colorectal neoplastic lesions were already present. The rationale behind this was to mimic the “treatment” of tumor patients by inhibition of BCL9/9L. Of note, *Bcl9/9l* ablation in primary tumors caused a compelling shift of the tumor phenotype: the neoplastic epithelium displayed the sudden loss of traits associated with EMT and invasiveness without affecting the surrounding healthy epithelial cells. This is important, as it provides impetus to the idea that inhibition of BCL9 function could in principle treat CRC in advanced stages. Second, genetic deletion of only the β‐catenin binding motif of BCL9/9L (the HD2 domain) recapitulated the full loss of the protein, instilling hope that the proverbial magic bullet should be developed to precisely hit this protein–protein interaction surface (Moor et al., 2015). Importantly, work aiming at inhibiting the β‐catenin/BCL9 interaction has already started. The Bienz group customized a β‐catenin‐binding ELISA‐based assay to screen through a huge library of chemicals, and measured that carnosic acid (CA), found in rosemary, inhibited the interaction with a relatively high specificity (Ki of 3.3 ± 1.8 μM), and dampened target gene expression in CRC SW480 cells and HCT116 cells (de la Roche et al., 2012). While these in vitro results await validation in animal models, rosemary extracts (which contain high levels of CA) have been shown to cause cell death and reduced tumor growth in vivo (Pérez‐Sánchez et al., 2019). The Carrasco group used a different approach. Based on the crystal structure of the β‐catenin/BCL9 complex (Sampietro et al., 2006), they designed a synthetic stabilized α‐helix to interfere with the HD2‐mediated interaction (Takada et al., 2012). The authors took advantage of the fine details of the peculiar contacts between the α‐helix HD2 and the surface groove of β‐catenin within the armadillo repeat 1 with which BCL9 binds. The resulting peptide (SAH‐BCL9) is cell permeable and efficiently inhibits the ability of BCL9 to bind to β‐catenin in a dose‐dependent manner. SAH‐BCL9 also inhibited cancer growth and metastasis in mouse xenograft models of CRC using human Colo320 and multiple myeloma (INA‐6) cells. Importantly also, the reduction in tumor growth was not accompanied with toxicity or considerable weight loss of the mice (Takada et al., 2012). Similar approaches were used by others for the rational design of small‐molecule inhibitors capable of inhibiting this physical interaction and the expression of target genes in cultured cells (Hoggard et al., 2015; Wisniewski, Yin, Teuscher, Zhang, & Ji, 2016). All these studies are extremely encouraging, even though they still lack the fundamental proof‐of‐concept that abrogating this interaction in vivo is safe and can be achieved efficiently and with high specificity. This is a fundamental prerequisite needed to push the use of these chemicals beyond the preclinical purposes of scientific research.

## 7 CONCLUSION: A MULTI‐DIMENSIONAL, HIGH‐TECH WNT SIGNALING

Historically, natural sciences have been dominated by a methodologically reductionist approach with the desire of explaining the smaller possible components of each natural phenomenon. While this attitude is necessary for the experimentalist, it should not become ontological reductionism, that is the belief that phenomena under investigation are only composed of the small parts identified. Analogously, as we have argued in this review article, the recognition of cell‐ and tissue‐specific properties of the Wnt transcriptional machinery greatly expands the original complexity conceived, both considering how the cascade is executed and the range of gene expression outputs produced. As in the worst reductionist nightmare, many are also the instances in which Wnt/β‐catenin signaling even exhibits bivalent properties within the same cell. In one example, WNT signals functionally support pluripotency but also promote cell differentiation of mESCs in an intertwined regulatory network with the pluripotency factors OCT4, SOX2 and NANOG (Kelly et al., 2011; Zhang, Peterson, Liu, Mcmahon, & Ohba, 2013). In another, β‐catenin mechanistically adopts two ways of action on different target regulatory sequences when recruiting LRH‐1 as co‐factor onto TCF7L2 target genes but also acting itself as co‐factor on LRH‐1 regulated sequences (Figure 3; Botrugno et al., 2004). Thus, future exploration of Wnt signaling requires novel methods for navigating its complex web of paths and interactions, and a comprehensive map describing the “system” picture is an important goal.

The development of high‐throughput technologies, such as those based on next generation sequencing (commonly referred to as NGS), made possible the study of genetics and molecular systems in a holistic manner, revolutionizing the research in many areas of the natural world (Hasin, Seldin, & Lusis, 2017). Several fields—collectively termed with the suffix “omics”—have emerged to study for example the global association between genetic variants and disease (Genomics), or the all‐inclusive gene or protein expression (Transcriptomics/Proteomics). The integration and combination of these different layers of biological mechanisms, in a multi‐omics approach, holds great promise in uncovering the complex mechanisms underlying the functioning of biological systems, including Wnt signaling. The amount of omics data that are being/will be generated is extensive and is pushing the co‐evolution of novel computational methods.

As a consequence of the Wnt signaling ductility, also cancer cells have numerous strategies at their disposal to exploit the Wnt/β‐catenin machinery for tumor growth and metastatic progression. This complex pathophysiological relationship, together with the fundamental role that Wnt signaling plays in many biological contexts, makes therapeutic targeting of Wnt components problematic. No single magic bullet will likely be discovered to cure all types of Wnt‐driven cancers, each of which will need to rely on a distinct attack strategy. The realization that “canonical” Wnt/β‐catenin signaling is a set of highly diverse routes is an important first step to recognize the complexity underlying Wnt‐driven cancers. Mapping and discovering the details of this diversity will subsequently be a crucial step toward the successful design of small inhibitory molecules that act in each specific context with minimal adverse effects on the other Wnt‐mediated functions. In this endeavor, increased knowledge of Wnt signaling and its components, especially regarding the β‐catenin/TCF transcriptional complex—which can be considered by and large the main route that Wnt signals undertake—is still necessary. The past and future discoveries about how Wnt signaling is mechanistically executed in each context will be integrated in an incremental pool of information that will likely be difficult to depict as a simplified linear series of events. In this regard, system biology comes to the rescue, and offers several ways to analyze and visualize complex systems by integrating omics data with methods from statistics and graph theory (Barabási, Gulbahce, & Loscalzo, 2011; Menche et al., 2015). On top of this, advanced mathematical modeling assisted by machine learning will enable the simulation of dynamics signaling networks, and the prediction of their consequences both in normal as well as in pathological conditions (Motta & Pappalardo, 2012). These models will self‐improve over‐time by adsorbing newly emerging large datasets (Camacho, Collins, Powers, Costello, & Collins, 2018). We cannot wait for the emergence of a multi‐dimensional Wnt signaling as it will appear to the multi‐omics eyes of a super‐computer.

## CONFLICT OF INTEREST

The authors declare the absence of any conflict of interest.

## AUTHOR CONTRIBUTIONS

**Simon Söderholm:** Conceptualization; data curation; formal analysis; investigation; project administration; resources; software; writing‐original draft; writing‐review and editing. **Claudio Cantù:** Conceptualization; funding acquisition; project administration; software; supervision; visualization; writing‐original draft; writing‐review and editing.

## RELATED WIREs ARTICLE

Mechanisms of Wnt signaling and control

## References

[wsbm1511-bib-0003] Afouda, B. A. , Nakamura, Y. , Shaw, S. , Charney, R. M. , Paraiso, K. D. , Blitz, I. L. , … Hoppler, S. (2020). Foxh1/Nodal defines context‐specific direct maternal Wnt/β‐catenin target gene regulation in early development. Science, 23, 101314. 10.1016/j.isci.2020.101314 PMC7347983 32650116

[wsbm1511-bib-0004] Albuquerque, C. , Breukel, C. , van der Liijt, R. , Fidalgo, P. , Lage, P. , Slors, F. J. M. , … Smits, R. (2002). The “just‐right” signaling model: APC somatic mutations are selected based on a specific level of activation of the beta‐catenin signaling cascade. Human Molecular Genetics, 11(13), 1549–1560. 10.1093/hmg/11.13.1549 12045208

[wsbm1511-bib-0005] Al‐Hendy, A. , Laknaur, A. , Diamond, M. P. , Ismail, N. , Boyer, T. G. , & Halder, S. K. (2017). Silencing Med12 gene reduces proliferation of human leiomyoma cells mediated via Wnt/β‐catenin signaling pathway. Endocrinology, 158(3), 592–603. 10.1210/en.2016-1097 27967206 PMC5460776

[wsbm1511-bib-0006] Andrews, P. G. P. , Lake, B. B. , Popadiuk, C. , & Kao, K. R. (2007). Requirement of Pygopus 2 in breast cancer. International Journal of Oncology, 30(2), 357–363 http://www.ncbi.nlm.nih.gov/pubmed/17203217 17203217

[wsbm1511-bib-0007] Archbold, H. C. , Broussard, C. , Chang, M. V. , & Cadigan, K. M. (2014). Bipartite recognition of DNA by TCF/pangolin is remarkably flexible and contributes to transcriptional responsiveness and tissue specificity of wingless signaling. PLoS Genetics, 10(9), e1004591. 10.1371/journal.pgen.1004591 25188465 PMC4154663

[wsbm1511-bib-0008] Arnold, C. D. , Gerlach, D. , Stelzer, C. , Boryn, L. M. , Rath, M. , & Stark, A. (2013). Genome‐wide quantitative enhancer activity maps identified by STARR‐seq. Science, 339(6123), 1074–1077. 10.1126/science.1232542 23328393

[wsbm1511-bib-0009] Arnold, S. J. , Stappert, J. , Bauer, A. , Kispert, A. , Herrmann, B. G. , & Kemler, R. (2000). Brachyury is a target gene of the Wnt/β‐catenin signaling pathway. Mechanisms of Development, 91(1–2), 249–258. 10.1016/S0925-4773(99)00309-3 10704849

[wsbm1511-bib-0010] Barabási, A. L. , Gulbahce, N. , & Loscalzo, J. (2011). Network medicine: A network‐based approach to human disease. Nature Reviews Genetics, 12(1), 56–68. 10.1038/nrg2918 PMC3140052 21164525

[wsbm1511-bib-0011] Barker, N. , Hurlstone, A. , Musisi, H. , Miles, A. , Bienz, M. , Clevers, H. , … Dean, D. (2001). The chromatin remodelling factor Brg‐1 interacts with beta‐catenin to promote target gene activation. The EMBO Journal, 20(17), 4935–4943. 10.1093/emboj/20.17.4935 11532957 PMC125268

[wsbm1511-bib-0012] Bauer, A. , Huber, O. , & Kemler, R. (1998). Pontin52, an interaction partner of β‐catenin, binds to the TATA box binding protein. Proceedings of the National Academy of Sciences of the United States of America, 95(25), 14787–14792. 10.1073/pnas.95.25.14787 9843967 PMC24527

[wsbm1511-bib-0013] Behrens, J. , von Kries, J. P. , Kühl, M. , Bruhn, L. , Wedlich, D. , Grosscheld, R. , & Birchmeier, W. (1996). Functional interaction of b‐catenin with the transcription factor LEF‐1. Nature, 382, 638–642. 10.1038/382638a0 8757136

[wsbm1511-bib-0014] Belenkaya, T. Y. , Han, C. , Standley, H. J. , Lin, X. , Houston, D. W. , & Heasman, J. (2002). Pygopus encodes a nuclear protein essential for wingless/Wnt signaling. Development, 129(17), 4089–4101. 12163411 10.1242/dev.129.17.4089

[wsbm1511-bib-0015] Benchabane, H. , Xin, N. , Tian, A. , Hafler, B. P. , Nguyen, K. , Ahmed, A. , & Ahmed, Y. (2011). Jerky/earthbound facilitates cell‐specific Wnt/wingless signalling by modulating β‐catenin‐TCF activity. EMBO Journal, 30(8), 1444–1458. 10.1038/emboj.2011.67 21399610 PMC3102276

[wsbm1511-bib-0016] Beroukhim, R. , Mermel, C. H. , Porter, D. , Wei, G. , Raychaudhuri, S. , Donovan, J. , … Meyerson, M. (2010). The landscape of somatic copy‐number alteration across human cancers. Nature, 463(7283), 899–905. 10.1038/nature08822 20164920 PMC2826709

[wsbm1511-bib-0017] Bhanot, P. , Brink, M. , Samos, C. H. , Hsieh, J. C. , Wang, Y. , Macke, J. P. , … Nusse, R. (1996). A new member of the frizzled family from Drosophila functions as a wingless receptor. Nature, 382(6588), 225–231. 10.1038/382225a0 8717036

[wsbm1511-bib-0018] Blauwkamp, T. A. , Chang, M. V. , & Cadigan, K. M. (2008). Novel TCF‐binding sites specify transcriptional repression by Wnt signalling. The EMBO Journal, 27(10), 1436–1446. 10.1038/emboj.2008.80 18418383 PMC2396397

[wsbm1511-bib-0019] Bosè, F. , Fugazza, C. , Casalgrandi, M. , Capelli, A. , Cunningham, J. M. , Zhao, Q. , … Ronchi, A. (2006). Functional interaction of CP2 with GATA‐1 in the regulation of Erythroid promoters. Molecular and Cellular Biology, 26(10), 3942–3954. 10.1128/mcb.26.10.3942-3954.2006 16648487 PMC1489008

[wsbm1511-bib-0020] Botrugno, O. A. , Fayard, E. , Annicotte, J. S. , Haby, C. , Brennan, T. , Wendling, O. , … Schoonjans, K. (2004). Synergy between LRH‐1 and β‐catenin induces G1 cyclin‐mediated cell proliferation. Molecular Cell, 15(4), 499–509. 10.1016/j.molcel.2004.07.009 15327767

[wsbm1511-bib-0021] Brannon, M. , Gomperts, M. , Sumoy, L. , Moon, R. T. , & Kimelman, D. (1997). A β‐catenin/XTcf‐3 complex binds to the siamois promoter to regulate dorsal axis specification in Xenopus. *Genes and* . Development, 11(18), 2359–2370. 10.1101/gad.11.18.2359 PMC316518 9308964

[wsbm1511-bib-0022] Brantjes, H. , Roose, J. , van de Wetering, M. , & Clevers, H. (2001). All Tcf HMG box transcription factors interact with Groucho‐related co‐repressors. Nucleic Acids Research, 29(7), 1410–1419. 10.1093/nar/29.7.1410 11266540 PMC31284

[wsbm1511-bib-0023] Brembeck, F. H. , Schwarz‐Romond, T. , Bakkers, J. , Wilhelm, S. , Hammerschmidt, M. , & Birchmeier, W. (2004). Essential role of BCL9‐2 in the switch between beta‐catenin's adhesive and transcriptional functions. Genes & Development, 18(18), 2225–2230. 15371335 10.1101/gad.317604 PMC517514

[wsbm1511-bib-0024] Brembeck, F. H. , Wiese, M. , Zatula, N. , Grigoryan, T. , Dai, Y. , Fritzmann, J. , & Birchmeier, W. (2011). BCL9‐2 promotes early stages of intestinal tumor progression. Gastroenterology, 141(4), 1359–1370. 10.1053/j.gastro.2011.06.039 21703997

[wsbm1511-bib-0025] Bronstein, R. , Levkovitz, L. , Yosef, N. , Yanku, M. , Ruppin, E. , Sharan, R. , … Segal, D. (2010). Transcriptional regulation by CHIP/LDB complexes. PLoS Genetics, 6(8), 12–15. 10.1371/journal.pgen.1001063 PMC2921152 20730086

[wsbm1511-bib-0026] Brunner, E. , Peter, O. , Schweizer, L. , & Basler, K. (1997). Pangolin encodes a Lef‐1 homologue that acts downstream of Armadillo to transduce the wingless signal in Drosophila. Nature, 385(6619), 829–833. 10.1038/385829a0 9039917

[wsbm1511-bib-0027] Buchert, M. , Athineos, D. , Abud, H. E. , Burke, Z. D. , Faux, M. C. , Samuel, M. S. , … Ernst, M. (2010). Genetic dissection of differential signaling threshold requirements for the Wnt/β‐catenin pathway in vivo. PLoS Genetics, 6(1), e1000816. 10.1371/journal.pgen.1000816 20084116 PMC2800045

[wsbm1511-bib-0028] Cadigan, K. M. , & Waterman, M. L. (2012). TCF/LEFs and Wnt signaling in the nucleus. Cold Spring Harbor Perspectives in Biology, 4(11), a007906–a007906. 10.1101/cshperspect.a007906 23024173 PMC3536346

[wsbm1511-bib-0029] Cadigan, K. M. , & Nusse, R. (1997). Wnt signaling: A common theme in animal development. Genes and Development, 11(24), 3286–3305. 10.1101/gad.11.24.3286 9407023

[wsbm1511-bib-0030] Cai, Y. , Jin, J. , Tomomori‐Sato, C. , Sato, S. , Sorokina, I. , Parmely, T. J. , … Conaway, J. W. (2003). Identification of new subunits of the multiprotein mammalian TRRAP/TIP60‐containing histone acetyltransferase complex. Journal of Biological Chemistry, 278(44), 42733–42736. 10.1074/jbc.C300389200 12963728

[wsbm1511-bib-0031] Calo, E. , & Wysocka, J. (2013). Modification of enhancer chromatin: What, how, and why? Molecular Cell, 49(5), 825–837. 10.1016/j.molcel.2013.01.038 23473601 PMC3857148

[wsbm1511-bib-0032] Camacho, D. M. , Collins, K. M. , Powers, R. K. , Costello, J. C. , & Collins, J. J. (2018). Next‐generation machine learning for biological networks. Cell, 173(7), 1581–1592. 10.1016/j.cell.2018.05.015 29887378

[wsbm1511-bib-0033] Cantù, C. , Felker, A. , Zimmerli, D. , Prummel, K. D. , Cabello, E. M. , Chiavacci, E. , … Mosimann, C. (2018). Mutations in Bcl9 and Pygo genes cause congenital heart defects by tissue‐specific perturbation of Wnt/β‐catenin signaling. Genes and Development, 32(21–22), 1443–1458. 10.1101/gad.315531.118 30366904 PMC6217730

[wsbm1511-bib-0034] Cantù, C. , Pagella, P. , Shajiei, T. D. , Zimmerli, D. , Valenta, T. , Hausmann, G. , … Mitsiadis, T. A. (2017). A cytoplasmic role of Wnt/β‐catenin transcriptional cofactors Bcl9, Bcl9l, and Pygopus in tooth enamel formation. Science Signaling, 10(465), 1–11. 10.1126/scisignal.aah4598 28174279

[wsbm1511-bib-0035] Cantù, C. , Valenta, T. , & Basler, K. (2013). A RING finger to wed TCF and β‐catenin. EMBO Reports, 14(4), 295–296. 10.1038/embor.2013.21 23478335 PMC3615660

[wsbm1511-bib-0036] Cantù, C. , Valenta, T. , Hausmann, G. , Vilain, N. , Aguet, M. , & Basler, K. (2013). The Pygo2‐H3K4me2/3 interaction is dispensable for mouse development and Wnt signaling‐dependent transcription. Development (Cambridge, England), 140(11), 2377–2386. 10.1242/dev.093591 23637336

[wsbm1511-bib-0037] Cantù, C. , Zimmerli, D. , Hausmann, G. , Valenta, T. , Moor, A. , Aguet, M. , & Basler, K. (2014). Pax6‐dependent, but β‐catenin‐independent, function of Bcl9 proteins in mouse lens development. Genes & Development, 28(17), 1879–1884. 10.1101/gad.246140.114 25184676 PMC4197948

[wsbm1511-bib-0038] Cantu', C. , Grande, V. , Alborelli, I. , Cassinelli, L. , Cantu', I. , Colzani, M. T. , … Ronchi, A. (2011). A highly conserved SOX6 double binding site mediates SOX6 gene downregulation in erythroid cells. Nucleic Acids Research, 39(2), 486–501. 10.1093/nar/gkq819 20852263 PMC3025548

[wsbm1511-bib-0039] Carrasco, D. R. , Tonon, G. , Huang, Y. , Zhang, Y. , Sinha, R. , Feng, B. , … DePinho, R. A. (2006). High‐resolution genomic profiles define distinct clinico‐pathogenetic subgroups of multiple myeloma patients. Cancer Cell, 9(4), 313–325. 10.1016/j.ccr.2006.03.019 16616336

[wsbm1511-bib-0040] Cavallo, R. , Rubenstein, D. , & Peifer, M. (1997). Armadillo and dTCF: A marriage made in the nucleus. Current Opinion in Genetics and Development, 7(4), 459–466. 10.1016/S0959-437X(97)80071-8 9309175

[wsbm1511-bib-0041] Chang, J. L. , Chang, M. V. , Barolo, S. , & Cadigan, K. M. (2008). Regulation of the feedback antagonist naked cuticle by wingless signaling. Developmental Biology, 321(2), 446–454. 10.1016/j.ydbio.2008.05.551 18585374 PMC2892857

[wsbm1511-bib-0042] Chang, M. V. , Chang, J. L. , Gangopadhyay, A. , Shearer, A. , & Cadigan, K. M. (2008). Activation of wingless targets requires bipartite recognition of DNA by TCF. Current Biology, 18(23), 1877–1881. 10.1016/j.cub.2008.10.047 19062282 PMC3105462

[wsbm1511-bib-0043] Chen, J. , Rajasekaran, M. , Xia, H. , Kong, S. N. , Deivasigamani, A. , Sekar, K. , … Hui, K. M. (2018). CDK 1‐mediated BCL 9 phosphorylation inhibits clathrin to promote mitotic Wnt signalling. The EMBO Journal, 37(20), 1–18. 10.15252/embj.201899395 30217955 PMC6187222

[wsbm1511-bib-0044] Chien, A. J. , Moore, E. C. , Lonsdorf, A. S. , Kulikauskas, R. M. , Rothberg, B. G. , Berger, A. J. , … Moon, R. T. (2009). Activated Wnt/β‐catenin signaling in melanoma is associated with decreased proliferation in patient tumors and a murine melanoma model. Proceedings of the National Academy of Sciences of the United States of America, 106(4), 1193–1198. 10.1073/pnas.0811902106 19144919 PMC2626610

[wsbm1511-bib-0045] Cronauer, M. V. , Schulz, W. A. , Ackermann, R. , & Burchardt, M. (2005). Effects of WNT/beta‐catenin pathway activation on signaling through T‐cell factor and androgen receptor in prostate cancer cell lines. International Journal of Oncology, 26(4), 1033–1040. 10.3892/ijo.26.4.1033 15753999

[wsbm1511-bib-0046] Daniels, D. L. , Eklof Spink, K. , & Weis, W. I. (2001). β‐catenin: Molecular plasticity and drug design. Trends in Biochemical Sciences, 26(11), 672–678. 10.1016/S0968-0004(01)01952-1 11701326

[wsbm1511-bib-0047] de la Roche, M. , & Bienz, M. (2007). Wingless‐independent association of pygopus with dTCF target genes. Current Biology: CB, 17(6), 556–561. 10.1016/j.cub.2007.01.063 17320388

[wsbm1511-bib-0048] de la Roche, M. , Rutherford, T. J. , Gupta, D. , Veprintsev, D. B. , Saxty, B. , Freund, S. M. , & Bienz, M. (2012). An intrinsically labile α‐helix abutting the BCL9‐binding site of β‐catenin is required for its inhibition by carnosic acid. Nature Communications, 3, 680. 10.1038/ncomms1680 PMC3293410 22353711

[wsbm1511-bib-0049] Deka, J. , Wiedemann, N. , Anderle, P. , Murphy‐Seiler, F. , Bultinck, J. , Eyckerman, S. , … Aguet, M. (2010). Bcl9/Bcl9l are critical for Wnt‐mediated regulation of stem cell traits in colon epithelium and adenocarcinomas. Cancer Research, 70(16), 6619–6628. 10.1158/0008-5472.CAN-10-0148 20682801

[wsbm1511-bib-0050] Del Rizzo, P. A. , & Trievel, R. C. (2011). Substrate and product specificities of SET domain methyltransferases. Epigenetics, 6(9), 1059–1067. 10.4161/epi.6.9.16069 21847010 PMC3225744

[wsbm1511-bib-0051] Dickinson, D. J. , Nelson, W. J. , & Weis, W. I. (2011). A polarized epithelium organized by β‐ and α‐catenin predates cadherin and metazoan origins. Science, 331(6022), 1336–1339. 10.1126/science.1199633 21393547 PMC3152298

[wsbm1511-bib-0052] Doumpas, N. , Lampart, F. , Robinson, M. D. , Lentini, A. , Nestor, C. E. , Cantù, C. , & Basler, K. (2019). TCF/LEF dependent and independent transcriptional regulation of Wnt/β‐catenin target genes. The EMBO Journal, 38(2), e98873. 10.15252/embj.201798873 30425074 PMC6331726

[wsbm1511-bib-0053] Draganova, K. , Zemke, M. , Zurkirchen, L. , Valenta, T. , Cantù, C. , Okoniewski, M. , … Sommer, L. (2014). Wnt/β‐catenin signaling regulates sequential fate decisions of murine cortical precursor cells. Stem Cells (Dayton, Ohio), 33, 170–182. 10.1002/stem.1820 25182747

[wsbm1511-bib-0054] Du, S. J. , Purcell, S. M. , Christian, J. L. , McGrew, L. L. , & Moon, R. T. (1995). Identification of distinct classes and functional domains of Wnts through expression of wild‐type and chimeric proteins in Xenopus embryos. Molecular and Cellular Biology, 15(5), 2625–2634. 10.1128/mcb.15.5.2625 7739543 PMC230492

[wsbm1511-bib-0055] Emami, K. H. , Nguyen, C. , Ma, H. , Kim, D. H. , Jeong, K. W. , Eguchi, M. , … Kahn, M. (2004). A small molecule inhibitor of β‐catenin/cyclic AMP response element‐binding protein transcription. Proceedings of the National Academy of Sciences of the United States of America, 101(34), 12682–12687. 10.1073/pnas.0404875101 15314234 PMC515116

[wsbm1511-bib-0056] Essers, M. A. G. , de Vires‐Smiths, L. M. M. , Barke, N. , Polderman, P. E. , Burgering, B. M. T. , & Kirswagen, H. C. (2005). Functional interaction between beta‐catenin and FOXO in oxidative stress signaling. Science, 308(5725), 1181–1184. 10.1126/science.1109083 15905404

[wsbm1511-bib-0057] Fan, X. , Song, J. , Zhao, Z. , Chen, M. , Tu, J. , Lu, C. , … Ji, J. (2019). Piplartine suppresses proliferation and invasion of hepatocellular carcinoma by LINC01391‐modulated Wnt/β‐catenin pathway inactivation through ICAT. Cancer Letters, 460(June), 119–127. 10.1016/j.canlet.2019.06.008 31207322

[wsbm1511-bib-0058] Farley, E. K. , Olson, K. M. , Zhang, W. , Brandt, A. J. , Rokhsar, D. S. , & Levine, M. S. (2015). Suboptimization of developmental enhancers. Science, 350(6258), 325–328. 10.1126/science.aac6948 26472909 PMC4970741

[wsbm1511-bib-0059] Fiedler, M. , Graeb, M. , Mieszczanek, J. , Rutherford, T. J. , Johnson, C. M. , & Bienz, M. (2015). An ancient Pygo‐dependent Wnt enhanceosome integrated by chip/LDB‐SSDP. eLife, 4(AUGUST2015), 1–22. 10.7554/eLife.09073 PMC4571689 26312500

[wsbm1511-bib-0060] Fiedler, M. , Sánchez‐Barrena, M. J. , Nekrasov, M. , Mieszczanek, J. , Rybin, V. , Müller, J. , … Bienz, M. (2008). Decoding of methylated histone H3 tail by the Pygo‐BCL9 Wnt signaling complex. Molecular Cell, 30(4), 507–518. 10.1016/j.molcel.2008.03.011 18498752 PMC2726290

[wsbm1511-bib-0061] Firestein, R. , Bass, A. J. , Kim, S. Y. , Dunn, I. F. , Silver, S. J. , Guney, I. , … Hahn, W. C. (2008). CDK8 is a colorectal cancer oncogene that regulates β‐catenin activity. Nature, 455(7212), 547–551. 10.1038/nature07179 18794900 PMC2587138

[wsbm1511-bib-0062] Flanagan, D. J. , Vincan, E. , & Phesse, T. J. (2017). Winding back Wnt signalling: Potential therapeutic targets for treating gastric cancers. British Journal of Pharmacology, 174(24), 4666–4683. 10.1111/bph.13890 28568899 PMC5727303

[wsbm1511-bib-0063] Fong, N. , Saldi, T. , Sheridan, R. M. , Cortazar, M. A. , & Bentley, D. L. (2017). RNA pol II dynamics modulate co‐transcriptional chromatin modification, CTD phosphorylation, and transcriptional direction. Molecular Cell, 66(4), 546–557.e3. 10.1016/j.molcel.2017.04.016 28506463 PMC5488731

[wsbm1511-bib-0064] Franz, A. , Shlyueva, D. , Brunner, E. , Stark, A. , & Basler, K. (2017). Probing the canonicity of the Wnt/wingless signaling pathway. PLoS Genetics, 13(4), e1006700. 10.1371/journal.pgen.1006700 28369070 PMC5393890

[wsbm1511-bib-0065] Funayama, N. , Fagotto, F. , McCrea, P. , & Gumbiner, B. M. (1995). Embryonic axis induction by the armadillo repeat domain of β‐catenin: Evidence for intracellular signaling. Journal of Cell Biology, 128(5), 959–968. 10.1083/jcb.128.5.959 7876319 PMC2120405

[wsbm1511-bib-0066] Gallet, A. , Angelats, C. , Erkener, A. , Charroux, B. , Fasano, L. , & Kerridge, S. (1999). The C‐terminal domain of Armadillo binds to hypophosphorylated Teashirt to modulate wingless signalling in Drosophila. The EMBO Journal, 18(8), 2208–2217. 10.1093/emboj/18.8.2208 10205174 PMC1171304

[wsbm1511-bib-0001] Gallet, A. , Erkner, A. , Charroux, B. , Fasano, L. , & Kerridge, S. (1998). Trunk‐specific modulation of wingless signalling in Drosophila by Teashirt binding to Armadillo. Current Biology, 8(16), 893–902. 10.1016/S0960-9822(07)00369-7 9707400

[wsbm1511-bib-0067] Gammons, M. , & Bienz, M. (2018). Multiprotein complexes governing Wnt signal transduction. Current Opinion in Cell Biology, 51, 42–49. 10.1016/j.ceb.2017.10.008 29153704

[wsbm1511-bib-0068] Gavin, B. J. , McMahon, J. A. , & McMahon, A. P. (1990). Expression of multiple novel Wnt‐1/int‐1‐related genes during fetal and adult mouse development. Genes & Development, 4(12B), 2319–2332. 2279700 10.1101/gad.4.12b.2319

[wsbm1511-bib-0069] Gay, D. M. , Ridgway, R. A. , Müeller, M. , Hodder, M. C. , Hedley, A. , Clark, W. , … Sansom, O. J. (2019). Loss of BCL9/9l suppresses Wnt driven tumourigenesis in models that recapitulate human cancer. Nature Communications, 10(1), 1–16. 10.1038/s41467-019-08586-3 PMC6374445 30760720

[wsbm1511-bib-0070] Gong, Y. , Lazaris, C. , Sakellaropoulos, T. , Lozano, A. , Kambadur, P. , Ntziachristos, P. , … Tsirigos, A. (2018). Stratification of TAD boundaries reveals preferential insulation of super‐enhancers by strong boundaries. Nature Communications, 9(1), 542. 10.1038/s41467-018-03017-1 PMC5803259 29416042

[wsbm1511-bib-0071] Griffin, C. T. , Curtis, C. D. , Davis, R. B. , Muthukumar, V. , & Magnuson, T. (2011). The chromatin‐remodeling enzyme BRG1 modulates vascular Wnt signaling at two levels. Proceedings of the National Academy of Sciences of the United States of America, 108(6), 2282–2287. 10.1073/pnas.1013751108 21262838 PMC3038709

[wsbm1511-bib-0072] Groden, J. , Thliveris, A. , Samowitz, W. , Carlson, M. , Gelbert, L. , Albertsen, H. , … White, R. (1991). Identification and characterization of the familial adenomatous polyposis coli gene. Cell, 66(3), 589–600. 10.1016/0092-8674(81)90021-0 1651174

[wsbm1511-bib-0073] Gu, B. , Sun, P. , Yuan, Y. , Moraes, R. C. , Li, A. , Teng, A. , … Dai, X. (2009). Pygo2 expands mammary progenitor cells by facilitating histone H3 K4 methylation. The Journal of Cell Biology, 185(5), 811–826. 10.1083/jcb.200810133 19487454 PMC2711593

[wsbm1511-bib-0074] Gu, B. , Watanabe, K. , Sun, P. , Fallahi, M. , & Dai, X. (2013). Chromatin effector Pygo2 mediates wnt‐notch crosstalk to suppress luminal/alveolar potential of mammary stem and basal cells. Cell Stem Cell, 13(1), 48–61. 10.1016/j.stem.2013.04.012 23684539 PMC3703489

[wsbm1511-bib-0075] Habib, S. J. , Chen, B. C. , Tsai, F. C. , Anastassiadis, K. , Meyer, T. , Betzig, E. , & Nusse, R. (2013). A localized Wnt signal orients asymmetric stem cell division in vitro. Science, 339(6126), 1445–1448. 10.1126/science.1231077 23520113 PMC3966430

[wsbm1511-bib-0076] Haegel, H. , Larue, L. , Ohsugi, M. , Fedorov, L. , Herrenknecht, K. , & Kemler, R. (1995). Lack of beta‐catenin affects mouse development at gastrulation. Development (Cambridge, England), 121(11), 3529–3537. http://www.ncbi.nlm.nih.gov/pubmed/8582267 10.1242/dev.121.11.3529 8582267

[wsbm1511-bib-0077] Hasin, Y. , Seldin, M. , & Lusis, A. (2017). Multi‐omics approaches to disease. Genome Biology, 18(1), 83. 10.1186/s13059-017-1215-1 28476144 PMC5418815

[wsbm1511-bib-0078] Haxaire, C. , Haÿ, E. , & Geoffroy, V. (2016). Runx2 controls bone Resorption through the Down‐regulation of the Wnt pathway in osteoblasts. American Journal of Pathology, 186(6), 1598–1609. 10.1016/j.ajpath.2016.01.016 27083516

[wsbm1511-bib-0079] He, T. C. , Sparks, A. B. , Rago, C. , Hermeking, H. , Zawel, L. , Da Costa, L. T. , … Kinzler, K. W. (1998). Identification of c‐MYC as a target of the APC pathway. Science, 281(5382), 1509–1512. 10.1126/science.281.5382.1509 9727977

[wsbm1511-bib-0080] Hecht, A. , Litterst, C. M. , Huber, O. , & Kemler, R. (1999). Functional characterization of multiple transactivating elements in β‐ catenin, some of which interact with the TATA‐binding protein in vitro. Journal of Biological Chemistry, 274(25), 18017–18025. 10.1074/jbc.274.25.18017 10364252

[wsbm1511-bib-0081] Hecht, A. , Vleminckx, K. , STemmler, M. P. , van Roy, F. , & Kemler, R. (2000). The p300/CBP acetyltransferases function as transcriptional coactivators of beta‐catenin in vertebrates. The EMBO Journal, 19(8), 1839–1850. 10.1093/emboj/19.8.1839 10775268 PMC302022

[wsbm1511-bib-0082] Heckl, M. , Schmoeckel, E. , Hertlein, L. , Rottmann, M. , Jeschke, U. , & Mayr, D. (2018). The ARID1A, p53 and ß‐catenin statuses are strong prognosticators in clear cell and endometrioid carcinoma of the ovary and the endometrium. PLoS One, 13(2), 1–17. 10.1371/journal.pone.0192881 PMC5815611 29451900

[wsbm1511-bib-0083] Hikasa, H. , & Sokol, SY. (2013). Wnt signaling in vertebrate axis specification. Cold Spring Harb Perspectives in Biol, 5(1), 1–20. 10.3390/genes11050538. PMC3579404 22914799

[wsbm1511-bib-0084] Hnisz, D. , Schuijers, J. , Lin, C. Y. , Weintraub, A. S. , Abraham, B. J. , Lee, T. I. , … Young, R. A. (2015). Convergence of developmental and oncogenic signaling pathways at transcriptional super‐enhancers. Molecular Cell, 58(2), 362–370. 10.1016/j.molcel.2015.02.014 25801169 PMC4402134

[wsbm1511-bib-0085] Hoffmans, R. , Städeli, R. , & Basler, K. (2005). Pygopus and legless provide essential transcriptional coactivator functions to armadillo/beta‐catenin. Current Biology, 15(13), 1207–1211. 16005293 10.1016/j.cub.2005.05.054

[wsbm1511-bib-0086] Hoggard, L. R. , Zhang, Y. , Zhang, M. , Panic, V. , Wisniewski, J. A. , & Ji, H. (2015). Rational design of selective small‐molecule inhibitors for β‐catenin/B‐cell lymphoma 9 protein–protein interactions. Journal of the American Chemical Society, 137(38), 12249–12260. 10.1021/jacs.5b04988 26352795

[wsbm1511-bib-0087] Hoogeboom, D. , Essers, M. A. G. , Polderman, P. E. , Voets, E. , Smits, L. M. M. , & Burgering, B. M. T. (2008). Interaction of FOXO with β‐catenin inhibits β‐catenin/T cell factor activity. Journal of Biological Chemistry, 283(14), 9224–9230. 10.1074/jbc.M706638200 18250171

[wsbm1511-bib-0088] Hossain, M. Z. , Yu, Q. , Xu, M. , & Sen, J. M. (2008). ICAT expression disrupts ‐catenin‐TCF interactions and impairs survival of thymocytes and activated mature T cells. International Immunology, 20(7), 925–935. 10.1093/intimm/dxn051 18511409 PMC2556852

[wsbm1511-bib-0089] Hovanes, K. , Li, T. W. , Munguia, J. E. , Truong, T. , Milovanovic, T. , Lawrence Marsh, J. , … Waterman, M. L. (2001). Beta‐catenin‐sensitive isoforms of lymphoid enhancer factor‐1 are selectively expressed in colon cancer. Nature Genetics, 28(1), 53–57. 10.1038/88264 11326276

[wsbm1511-bib-0090] Hoverter, N. P. , Ting, J.‐H. , Sundaresh, S. , Baldi, P. , & Waterman, M. L. (2012). A WNT/p21 circuit directed by the C‐clamp, a sequence‐specific DNA binding domain in TCFs. Molecular and Cellular Biology, 32(18), 3648–3662. 10.1128/mcb.06769-11 22778133 PMC3430198

[wsbm1511-bib-0091] Huang, M.‐Y. , Chang, H.‐J. , Chung, F.‐Y. , Yang, M.‐J. , Yang, Y.‐H. , Wang, J. , & Lin, S.‐R. (2010). MMP13 is a potential prognostic marker for colorectal cancer MING‐YII. Anticancer Research, 31(12), 1265–1270. 10.3892/or_00000978 20878116

[wsbm1511-bib-0092] Huang, S. , Hölzel, M. , Knijnenburg, T. , Schlicker, A. , Roepman, P. , McDermott, U. , … Bernards, R. (2012). MED12 controls the response to multiple cancer drugs through regulation of TGF‐β receptor signaling. Cell, 151(5), 937–950. 10.1016/j.cell.2012.10.035 23178117 PMC3672971

[wsbm1511-bib-0093] Huber, A. H. , Nelson, W. J. , & Weis, W. I. (1997). Three‐dimensional structure of the armadillo repeat region of β‐catenin. Cell, 90(5), 871–882. 10.1016/S0092-8674(00)80352-9 9298899

[wsbm1511-bib-0094] Huber, O. , Korn, R. , McLaughlin, J. , Ohsugi, M. , Herrmann, B. G. , & Kemler, R. (1996). Nuclear localization of β‐catenin by interaction with transcription factor LEF‐1. Mechanisms of Development, 59(1), 3–10. 10.1016/0925-4773(96)00597-7 8892228

[wsbm1511-bib-0095] Idogawa, M. , Yamada, T. , Honda, K. , Sato, S. , Imai, K. , & Hirohashi, S. (2005). Poly(ADP‐ribose) polymerase‐1 is a component of the oncogenic T‐cell factor‐4/beta;‐catenin complex. Gastroenterology, 128(7), 1919–1936. 10.1053/j.gastro.2005.03.007 15940627

[wsbm1511-bib-0096] Ito, K. , Lim, A. C. B. , Salto‐Tellez, M. , Motoda, L. , Osato, M. , Chuang, L. S. H. , … Ito, Y. (2008). RUNX3 attenuates β‐catenin/T cell factors in intestinal tumorigenesis. Cancer Cell, 14(3), 226–237. 10.1016/j.ccr.2008.08.004 18772112

[wsbm1511-bib-0097] Jackstadt, R. , & Sansom, O. J. (2016). Mouse models of intestinal cancer. Journal of Pathology, 238(2), 141–151. 10.1002/path.4645 26414675 PMC4832380

[wsbm1511-bib-0098] Janody, F. , Martirosyan, Z. , Benlali, A. , & Treisman, J. E. (2003). Two subunits of the Drosophila mediator complex act together to control cell affinity. Development, 130(16), 3691–3701. 10.1242/dev.00607 12835386

[wsbm1511-bib-0099] Janovská, P. , & Bryja, V. (2017). Wnt signalling pathways in chronic lymphocytic leukaemia and B‐cell lymphomas. British Journal of Pharmacology, 174(24), 4701–4715. 10.1111/bph.13949 28703283 PMC5727250

[wsbm1511-bib-0100] Jenny, F. H. , & Basler, K. (2014). Powerful Drosophila screens that paved the wingless pathway. Fly (Austin), 8(4), 218–225. 10.4161/19336934.2014.985988 25565425 PMC4594357

[wsbm1511-bib-0101] Jho, E. , Zhang, T. , Domon, C. , Joo, C. , Freund, J. , & Costantini, F. (2002). Wnt/β ‐catenin/Tcf signaling induces the transcription of Axin2, a negative regulator of the signaling pathway Wnt/ß‐catenin/Tcf signaling induces the transcription of Axin2, a negative regulator of the signaling pathway. Molecular and Cellular Biology, 22(4), 1172–1183. 10.1128/MCB.22.4.1172 11809808 PMC134648

[wsbm1511-bib-0102] Jubb, A. M. , Chalasani, S. , Frantz, G. D. , Smits, R. , Grabsch, H. I. , Kavi, V. , … Koeppen, H. (2006). Achaete‐scute like 2 (ascl2) is a target of Wnt signalling and is upregulated in intestinal neoplasia. Oncogene, 25(24), 3445–3457. 10.1038/sj.onc.1209382 16568095

[wsbm1511-bib-0103] Jürgens, G. , Wieschaus, E. , Nüsslein‐Volhard, C. , & Kluding, H. (1984). Mutations affecting the pattern of the larval cuticle in *Drosophila melanogaster* ‐ II. Zygotic loci on the third chromosome. Wilhelm Roux's Archives of Developmental Biology, 193(5), 283–295. 10.1007/BF00848157 28305338

[wsbm1511-bib-0104] Kahler, R. A. , & Westendorf, J. J. (2003). Lymphoid enhancer factor‐1 and β‐catenin inhibit Runx2‐dependent transcriptional activation of the osteocalcin promoter. Journal of Biological Chemistry, 278(14), 11937–11944. 10.1074/jbc.M211443200 12551949

[wsbm1511-bib-0105] Kahn, M. (2014). Can we safely target the WNT pathway? Nature Reviews Drug Discovery, 13(7), 513–532. 24981364 10.1038/nrd4233 PMC4426976

[wsbm1511-bib-0106] Kaidi, A. , Williams, A. C. , & Paraskeva, C. (2007). Interaction between β‐catenin and HIF‐1 promotes cellular adaptation to hypoxia. Nature Cell Biology, 9(2), 210–217. 10.1038/ncb1534 17220880

[wsbm1511-bib-0107] Kelly, K. F. , Ng, D. Y. , Jayakumaran, G. , Wood, G. A. , Koide, H. , & Doble, B. W. (2011). β‐Catenin enhances Oct‐4 activity and reinforces pluripotency through a TCF‐independent mechanism. Cell Stem Cell, 8(2), 214–227. 10.1016/j.stem.2010.12.010 21295277 PMC3465368

[wsbm1511-bib-0108] Kelly, O. G. , Pinson, K. I. , & Skarnes, W. C. (2004). The Wnt co‐receptors Lrp5 and Lrp6 are essential for gastrulation in mice. Development, 131(12), 2803–2815. 10.1242/dev.01137 15142971

[wsbm1511-bib-0109] Kessler, R. , Hausmann, G. , & Basler, K. (2009). The PHD domain is required to link Drosophila Pygopus to legless/beta‐catenin and not to histone H3. Mechanisms of Development, 126(8–9), 752–759. 10.1016/j.mod.2009.04.003 19493659

[wsbm1511-bib-0110] Khare, R. , Leaman, R. , & Lu, Z. (2014). Accessing biomedical literature in the current information landscape. Methods in Molecular Biology, 1159, 47–75. 10.1007/978-1-4939-0709-0 24788259 PMC4593617

[wsbm1511-bib-0111] Kim, C.‐H. , Neiswender, H. , Baik, E. J. , Xiong, W. C. , & Mei, L. (2008). β‐Catenin interacts with MyoD and regulates its transcription activity. Molecular and Cellular Biology, 28(9), 2941–2951. 10.1128/mcb.01682-07 18316399 PMC2293083

[wsbm1511-bib-0112] Kim, S. , Xu, X. , Hecht, A. , & Boyer, T. G. (2006). Mediator is a transducer of Wnt/β‐catenin signaling. Journal of Biological Chemistry, 281(20), 14066–14075. 10.1074/jbc.M602696200 16565090

[wsbm1511-bib-0113] Kimura, K. , Ikoma, A. , Shibakawa, M. , Shimoda, S. , Harada, K. , Saio, M. , … Mizokami, M. (2017). Safety, tolerability, and preliminary efficacy of the anti‐fibrotic small molecule PRI‐724, a CBP/β‐catenin inhibitor, in patients with hepatitis C virus‐related cirrhosis: A single‐center, open‐label, dose escalation phase 1 trial. eBioMedicine, 23, 79–87. 10.1016/j.ebiom.2017.08.016 28844410 PMC5605374

[wsbm1511-bib-0114] Kinzler, K. W. , Nilbert, M. C. , Su, L. K. , Vogelstein, B. , Bryan, T. M. , Levy, D. B. , … Nakamura, Y. (1991). Identification of FAP locus genes from chromosome 5q21. Science, 253(5020), 661–665. 10.1126/science.1651562 1651562

[wsbm1511-bib-0115] Klemm, S. L. , Shipony, Z. , & Greenleaf, W. J. (2019). Chromatin accessibility and the regulatory epigenome. Nature Reviews Genetics, 20(4), 207–220. 10.1038/s41576-018-0089-8 30675018

[wsbm1511-bib-0116] Kokal, M. , Mirzakhani, K. , Pungsrinont, T. , & Baniahmad, A. (2020). Mechanisms of androgen receptor agonist‐ and antagonist‐mediated cellular senescence. Cancers (Basel), 12, E1833. 32650419 10.3390/cancers12071833 PMC7408918

[wsbm1511-bib-0117] Kormish, J. D. , Sinner, D. , & Zorn, A. M. (2009). Interactions between SOX factors and Wnt/β‐catenin signaling in development and disease. Developmental Dynamics, 239(1), 56–68. 10.1002/dvdy.22046 PMC3269784 19655378

[wsbm1511-bib-0118] Kouzmenko, A. P. , Takeyama, K. I. , Ito, S. , Furutani, T. , Sawatsubashi, S. , Maki, A. , … Kato, S. (2004). Wnt/β‐catenin and estrogen signaling converge in vivo. Journal of Biological Chemistry, 279(39), 40255–40258. 10.1074/jbc.C400331200 15304487

[wsbm1511-bib-0119] Kramps, T. , Peter, O. , Brunner, E. , Nellen, D. , Froesch, B. , Chatterjee, S. , … Basler, K. (2002). Wnt/wingless signaling requires BCL9/legless‐mediated recruitment of pygopus to the nuclear beta‐catenin‐TCF complex. Cell, 109(1), 47–60. http://www.ncbi.nlm.nih.gov/pubmed/11955446 11955446 10.1016/s0092-8674(02)00679-7

[wsbm1511-bib-0120] Kushner, P. J. , Agard, D. A. , Greene, G. L. , Scanlan, T. S. , Shiau, A. K. , Uht, R. M. , & Webb, P. (2000). Estrogen receptor pathways to AP‐1. Journal of Steroid Biochemistry & Molecular Biology, 74, 311–317. 11162939 10.1016/s0960-0760(00)00108-4

[wsbm1511-bib-0121] Lee, H. H. , & Frasch, M. (2000). Wingless effects mesoderm patterning and ectoderm segmentation events via induction of its downstream target sloppy paired. Development, 127(24), 5497–5508. 11076769 10.1242/dev.127.24.5497

[wsbm1511-bib-0123] Li, B. , Rheaume, C. , Teng, A. , Bilanchone, V. , Munguia, J. E. , Hu, M. , … Dai, X. (2007). Developmental phenotypes and reduced Wnt signaling in mice deficient for pygopus 2. Genesis, 45(5), 318–325. 10.1002/dvg 17458864

[wsbm1511-bib-0124] Li, J. , & Wang, C. Y. (2008). TBL1‐TBLR1 and β‐catenin recruit each other to Wnt target‐gene promoter for transcription activation and oncogenesis. Nature Cell Biology, 10(2), 160–169. 10.1038/ncb1684 18193033

[wsbm1511-bib-0125] Li, T. W.‐H. , Ting, J.‐H. T. , Yokoyama, N. N. , Bernstein, A. , van de Wetering, M. , & Waterman, M. L. (2006). Wnt activation and alternative promoter repression of LEF1 in Colon Cancer. Molecular and Cellular Biology, 26(14), 5284–5299. 10.1128/mcb.00105-06 16809766 PMC1592719

[wsbm1511-bib-0126] Li, V. S. W. , Ng, S. S. , Boersema, P. J. , Low, T. Y. , Karthaus, W. R. , Gerlach, J. P. , … Clevers, H. (2012). Wnt signaling through inhibition of β‐catenin degradation in an intact Axin1 complex. Cell, 149(6), 1245–1256. 10.1016/j.cell.2012.05.002 22682247

[wsbm1511-bib-0127] Licht, J. D. (2001). AML1 and the AML1‐ETO fusion protein in the pathogenesis of t(8;21) AML. Oncogene, 20(40 REV. ISS. 4, 5660–5679. 10.1038/sj.onc.1204593 11607817

[wsbm1511-bib-0128] Liu, F. , Chu, E. Y. , Watt, B. , Zhang, Y. , Gallant, N. M. , Andl, T. , … Millar, S. E. (2008). Wnt/beta‐catenin signaling directs multiple stages of tooth morphogenesis. Developmental Biology, 313(1), 210–224. 10.1016/j.ydbio.2007.10.016 18022614 PMC2843623

[wsbm1511-bib-0129] Ludlam, W. H. , Taylor, M. H. , Tanner, K. G. , Denu, J. M. , Goodman, R. H. , & Smolik, S. M. (2002). The Acetyltransferase activity of CBP is required for wingless activation and H4 acetylation in *Drosophila melanogaster* . Molecular and Cellular Biology, 22(11), 3832–3841. 10.1128/mcb.22.11.3832-3841.2002 11997517 PMC133831

[wsbm1511-bib-0130] Lyashenko, N. , Winter, M. , Migliorini, D. , Biechele, T. , Moon, R. T. , & Hartmann, C. (2011). Differential requirement for the dual functions of β‐catenin in embryonic stem cell self‐renewal and germ layer formation. Nature Cell Biology, 13(7), 753–761. 10.1038/ncb2260 21685890 PMC3130149

[wsbm1511-bib-0131] Lyou, Y. , Habowski, A. N. , Chen, G. T. , & Waterman, M. L. (2017). Inhibition of nuclear Wnt signalling: Challenges of an elusive target for cancer therapy. British Journal of Pharmacology, 174(24), 4589–4599. 10.1111/bph.13963 28752891 PMC5727325

[wsbm1511-bib-0132] Ma, H. , Nguyen, C. , Lee, K. S. , & Kahn, M. (2005). Differential roles for the coactivators CBP and p300 on TCF/β‐catenin‐mediated survivin gene expression. Oncogene, 24(22), 3619–3631. 10.1038/sj.onc.1208433 15782138

[wsbm1511-bib-0133] Madan, B. , McDonald, M. J. , Foxa, G. E. , Diegel, C. R. , Williams, B. O. , & Virshup, D. M. (2018). Bone loss from Wnt inhibition mitigated by concurrent alendronate therapy. Bone Research, 6(1), 17. 10.1038/s41413-018-0017-8 29844946 PMC5968037

[wsbm1511-bib-0134] Mani, M. , Carrasco, D. E. , Zhang, Y. , Takada, K. , Gatt, M. E. , Dutta‐Simmons, J. , … Carrasco, D. R. (2009). BCL9 promotes tumor progression by conferring enhanced proliferative, metastatic, and angiogenic properties to cancer cells. Cancer Research, 69(19), 7577–7586. 10.1158/0008-5472.CAN-09-0773 19738061 PMC4321734

[wsbm1511-bib-0135] Maretto S. , Cordenonsi M. , Dupont S. , Braghetta P. , Broccoli V. , Hassan A. B. , … Piccolo S. (2003). Mapping Wnt/ ‐catenin signaling during mouse development and in colorectal tumors. *Proceedings of the National Academy of Sciences,* 100(6), 3299–3304. 10.1073/pnas.0434590100. PMC152286 12626757

[wsbm1511-bib-0136] Mathur, R. , Alver, B. H. , San Roman, A. K. , Wilson, B. G. , Wang, X. , Agoston, A. T. , … Roberts, C. W. M. (2017). ARID1A loss impairs enhancer‐mediated gene regulation and drives colon cancer in mice. Nature Genetics, 49(2), 296–302. 10.1038/ng.3744 27941798 PMC5285448

[wsbm1511-bib-0137] Matthews, J. M. , & Visvader, J. E. (2003). LIM‐domain‐binding protein 1: A multifunctional cofactor that interacts with diverse proteins. EMBO Reports, 4(12), 1132–1137. 10.1038/sj.embor.7400030 14647207 PMC1326422

[wsbm1511-bib-0138] Mayall, T. P. , Sheridan, P. L. , Montminy, M. R. , & Jones, K. A. (1997). Distinct roles for P‐CREB and LEF‐1 in TCRα enhancer assembly and activation on chromatin templates in vitro. Genes and Development, 11(7), 887–899. 10.1101/gad.11.7.887 9106660

[wsbm1511-bib-0139] Mayer, C. D. , de La Giclais, S. M. , Alsehly, F. , & Hoppler, S. (2020). Diverse LEF/TCF expression in human colorectal Cancer correlates with altered Wnt‐regulated Transcriptome in a meta‐analysis of patient biopsies. Genes, 11(5), 538. 10.3390/genes11050538 PMC7288467 32403323

[wsbm1511-bib-0140] Mazzoni, S. M. , & Fearon, E. R. (2014). AXIN1 and AXIN2 variants in gastrointestinal cancers. Cancer Letters, 355(1), 1–8. 10.1016/j.canlet.2014.09.018 25236910 PMC4298141

[wsbm1511-bib-0141] Mccrea, P. D. , Turck, C. W. , & Gumbiner, B. (1991). A homolog of the armadillo protein in Drosophila (plakoglobin) associated with E‐cadherin. Science, 254(5036), 1359–1361. 10.1126/science.1962194 1962194

[wsbm1511-bib-0142] McMahon, A. P. , & Moon, R. T. (1989). Ectopic expression of the proto‐oncogene int‐1 in Xenopus embryos leads to duplication of the embryonic axis. Cell, 58(6), 1075–1084. 10.1016/0092-8674(89)90506-0 2673541

[wsbm1511-bib-0143] Menche, J. , Sharma, A. , Kitsak, M. , Ghiassian, S. D. , Vidal, M. , Loscalzo, J. , & Barabási, A. L. (2015). Uncovering disease‐disease relationships through the incomplete interactome. Science, 347(6224), 841. 10.1126/science.1257601 PMC4435741 25700523

[wsbm1511-bib-0144] Merika, M. , & Thanos, D. (2001). Enhanceosomes. Current Opinion in Genetics and Development, 11(2), 205–208. 10.1016/S0959-437X(00)00180-5 11250145

[wsbm1511-bib-0145] Mevel, R. , Draper, J. E. , Lie‐A‐Ling, M. , Kouskoff, V. , & Lacaud, G. (2019). RUNX transcription factors: Orchestrators of development. Development (Cambridge), 146, 1–19. 10.1242/dev.148296 31488508

[wsbm1511-bib-0146] Mieszczanek, J. , de la Roche, M. , & Bienz, M. (2008). A role of Pygopus as an anti‐repressor in facilitating Wnt‐dependent transcription. Proceedings of the National Academy of Sciences of the United States of America, 105(49), 19324–19329. 10.1073/pnas.0806098105 19036929 PMC2614760

[wsbm1511-bib-0147] Mieszczanek, J. , van Tienen, L. M. , Ibrahim, A. E. K. , Winton, D. J. , & Bienz, M. (2019). Bcl9 and Pygo synergise downstream of Apc to effect intestinal neoplasia in FAP mouse models. Nature Communications, 10(1), 724. 10.1038/s41467-018-08164-z PMC6374407 30760710

[wsbm1511-bib-0148] Milán, M. , Diaz‐Benjumea, F. J. , & Cohen, S. M. (1998). Beadex encodes an LMO protein that regulates Apterous LIM‐homeodomain activity in Drosophila wing development: A model for LMO oncogene function. Genes and Development, 12(18), 2912–2920. 10.1101/gad.12.18.2912 9744867 PMC317163

[wsbm1511-bib-0149] Miller, T. C. R. , Rutherford, T. J. , Birchall, K. , Chugh, J. , Fiedler, M. , & Bienz, M. (2014). Competitive binding of a benzimidazole to the histone‐binding pocket of the Pygo PHD finger. ACS Chemical Biology, 9(12), 2864–2874. 10.1021/cb500585s 25323450 PMC4330097

[wsbm1511-bib-0150] Miller, T. C. R. , Rutherford, T. J. , Johnson, C. M. , Fiedler, M. , & Bienz, M. (2010). Allosteric remodelling of the histone H3 binding pocket in the Pygo2 PHD finger triggered by its binding to the B9L/BCL9 co‐factor. Journal of Molecular Biology, 401(5), 969–984. 10.1016/j.jmb.2010.07.007 20637214 PMC2927781

[wsbm1511-bib-0151] Mitsiadis, T. a. , & Graf, D. (2009). Cell fate determination during tooth development and regeneration. Birth Defects Research. Part C, Embryo Today: Reviews, 87(3), 199–211. 10.1002/bdrc.20160 19750524

[wsbm1511-bib-0152] Mittal, P. , & Roberts, C. W. M. (2020). The SWI/SNF complex in cancer—Biology, biomarkers and therapy. Nature Reviews Clinical Oncology, 17, 435–448. 10.1038/s41571-020-0357-3 PMC8723792 32303701

[wsbm1511-bib-0153] Mohan, M. , Herz, H. M. , Takahashi, Y. H. , Lin, C. , Lai, K. C. , Zhang, Y. , … Shilatifard, A. (2010). Linking H3K79 trimethylation to Wnt signaling through a novel Dot1‐containing complex (DotCom). Genes and Development, 24(6), 574–589. 10.1101/gad.1898410 20203130 PMC2841335

[wsbm1511-bib-0154] Molenaar, M. , Van De Wetering, M. , Oosterwegel, M. , Peterson‐Maduro, J. , Godsave, S. , Korinek, V. , … Clevers, H. (1996). XTcf‐3 transcription factor mediates β‐catenin‐induced axis formation in xenopus embryos. Cell, 86(3), 391–399. 10.1016/S0092-8674(00)80112-9 8756721

[wsbm1511-bib-0155] Moor, A. E. , Anderle, P. , Cantù, C. , Rodriguez, P. , Wiedemann, N. , Baruthio, F. , … Aguet, M. (2015). BCL9/9L‐β‐catenin signaling is associated with poor outcome in colorectal Cancer. eBioMedicine, 2(12), 1932–1943. 10.1016/j.ebiom.2015.10.030 26844272 PMC4703711

[wsbm1511-bib-0156] Moparthi, L. , Pizzolato, G. , & Koch, S. (2019). Wnt activator FOXB2 drives the neuroendocrine differentiation of prostate cancer. Proceedings of the National Academy of Sciences of the United States of America, 116(44), 22189–22195. 10.1073/pnas.1906484116 31611391 PMC6825314

[wsbm1511-bib-0157] Moreira, S. , Polena, E. , Gordon, V. , Abdulla, S. , Mahendram, S. , Cao, J. , … Doble, B. W. (2017). A single TCF transcription factor, regardless of its activation capacity, is sufficient for effective Trilineage differentiation of ESCs. Cell Reports, 20(10), 2424–2438. 10.1016/j.celrep.2017.08.043 28877475

[wsbm1511-bib-0158] Mosimann, C. , Hausmann, G. , & Basler, K. (2006). Parafibromin/hyrax activates Wnt/Wg target gene transcription by direct association with beta‐catenin/Armadillo. Cell, 125(2), 327–341. 10.1016/j.cell.2006.01.053 16630820

[wsbm1511-bib-0159] Motta, S. , & Pappalardo, F. (2012). Mathematical modeling of biological systems. Briefings in Bioinformatics, 14(4), 411–422. 10.1093/bib/bbs061 23063928

[wsbm1511-bib-0160] Mouradov, D. , Sloggett, C. , Jorissen, R. N. , Love, C. G. , Li, S. , Burgess, A. W. , … Sieber, O. M. (2014). Colorectal cancer cell lines are representative models of the main molecular subtypes of primary cancer. Cancer Research, 74(12), 3238–3247. 10.1158/0008-5472.CAN-14-0013 24755471

[wsbm1511-bib-0161] Mukherjee, S. , Chaturvedi, P. , Rankin, SA. , Fish, MB. , Wlizla, M. , Paraiso, KD. , … Zorn, A. Sox17 and β‐catenin co‐occupy Wnt‐responsive enhancers to govern the endodermal gene regulatory network. eLife, (2020). 9, 1–26. 10.1101/2020.02.19.956565 PMC7498262 32894225

[wsbm1511-bib-0162] Nair, M. , Nagamori, I. , Sun, P. , Mishra, D. P. , Rhéaume, C. , Li, B. , … Dai, X. (2008). Nuclear regulator Pygo2 controls spermiogenesis and histone H3 acetylation. Developmental Biology, 320(2), 446–455. 10.1016/j.ydbio.2008.05.553 18614164 PMC2553271

[wsbm1511-bib-0163] Nateri, A. S. , Spencer‐Dene, B. , & Behrens, A. (2005). Interaction of phosphorylated c‐Jun with TCF4 regulates intestinal cancer development. Nature, 437(7056), 281–285. 10.1038/nature03914 16007074

[wsbm1511-bib-0164] Noordermeer, J. , Klingensmith, J. , Perrimon, N. , & Nusse, R. (1994). Dishevelled and armadillo act in the wingless signalling pathway in Drosophila. Nature, 367(6458), 80–83. 10.1038/367080a0 7906389

[wsbm1511-bib-0165] Nusse, R. , Brown, A. , Papkoff, J. , Scambler, P. , Shackleford, G. , McMahon, A. , … Varmus, H. (1991). A new nomenclature for int‐1 and related genes: The Wnt gene family. Cell, 64(2), 231. 10.1016/0092-8674(91)90633-A 1846319

[wsbm1511-bib-0166] Nusse, R. , & Varmus, H. E. (1982). Many tumors induced by the mouse mammary tumor virus contain a provirus integrated in the same region of the host genome. Cell, 31(1), 99–109. 10.1016/0092-8674(82)90409-3 6297757

[wsbm1511-bib-0167] Nusse, R. , & Clevers, H. (2017). Wnt/β‐catenin signaling, disease, and emerging therapeutic modalities. Cell, 169(6), 985–999. 10.1016/j.cell.2017.05.016 28575679

[wsbm1511-bib-0168] Nusse, R. , Van Ooyen, A. , Cox, D. , Fung, Y. K. T. , & Varmus, H. (1984). Mode of proviral activation of a putative mammary oncogene (int‐1) on mouse chromosome 15. Nature, 307(5947), 131–136. 10.1038/307131a0 6318122

[wsbm1511-bib-0169] Olson, L. E. , Tollkuhn, J. , Scafoglio, C. , Krones, A. , Zhang, J. , Ohgi, K. A. , … Rosenfeld, M. G. (2006). Homeodomain‐mediated β‐catenin‐dependent switching events dictate cell‐lineage determination. Cell, 125(3), 593–605. 10.1016/j.cell.2006.02.046 16678101

[wsbm1511-bib-0170] Orsulic, S. , & Peifer, M. (1996). An in vivo structure‐function study of Armadillo, the β‐catenin homologue, reveals both separate and overlapping regions of the protein required for cell adhesion and for wingless signaling. Journal of Cell Biology, 134(5), 1283–1300. 10.1083/jcb.134.5.1283 8794868 PMC2120977

[wsbm1511-bib-0171] Ozawa, M. , Baribault, H. , & Kemler, R. (1989). The cytoplasmic domain of the cell adhesion molecule uvomorulin associates with three independent proteins structurally related in different species. The EMBO Journal, 8(6), 1711–1717. 10.1002/j.1460-2075.1989.tb03563.x 2788574 PMC401013

[wsbm1511-bib-0172] Papaioannou, V. E. (2014). The t‐box gene family: Emerging roles in development, stem cells and cancer. Development (Cambridge), 141(20), 3819–3833. 10.1242/dev.104471 PMC4197708 25294936

[wsbm1511-bib-0173] Parker, D. S. , Jemison, J. , & Cadigan, K. M. (2002). Pygopus, a nuclear PHD‐finger protein required for wingless signaling in Drosophila. Development (Cambridge, England), 129(11), 2565–2576. http://www.ncbi.nlm.nih.gov/pubmed/12015286 10.1242/dev.129.11.2565 12015286

[wsbm1511-bib-0174] Parker, D. S. , Ni, Y. Y. , Chang, J. L. , Li, J. , & Cadigan, K. M. (2008). Wingless signaling induces widespread chromatin remodeling of target loci. Molecular and Cellular Biology, 28(5), 1815–1828. 10.1128/mcb.01230-07 18160704 PMC2258762

[wsbm1511-bib-0175] Peifer, M. , Rauskolb, C. , Williams, M. , Riggleman, B. , & Wieschaus, E. (1991). The segment polarity gene armadillo interacts with the wingless signaling pathway in both embryonic and adult pattern formation. Development, 111(4), 1029–1043. 1879348 10.1242/dev.111.4.1029

[wsbm1511-bib-0176] Peifer, M. , Sweeton, D. , Casey, M. , & Wieschaus, E. (1994). Wingless signal and Zeste‐white 3 kinase trigger opposing changes in the intracellular distribution of Armadillo. Development, 120(2), 369–380. 8149915 10.1242/dev.120.2.369

[wsbm1511-bib-0177] Pérez‐Sánchez, A. , Barrajón‐Catalán, E. , Ruiz‐Torres, V. , Agulló‐Chazarra, L. , Herranz‐López, M. , Valdés, A. , … Micol, V. (2019). Rosemary (*Rosmarinus officinalis*) extract causes ROS‐induced necrotic cell death and inhibits tumor growth in vivo. Scientific Reports, 9(1), 1–11. 10.1038/s41598-018-37173-7 30692565 PMC6349921

[wsbm1511-bib-0178] Pezone, A. , Zuchegna, C. , Tramontano, A. , Romano, A. , Russo, G. , de Rosa, M. , … Avvedimento, E. V. (2019). RNA stabilizes transcription‐dependent chromatin loops induced by nuclear hormones. Scientific Reports, 9(1), 1–12. 10.1038/s41598-019-40123-6 30850627 PMC6408484

[wsbm1511-bib-0179] Powell, S. M. , Zilz, N. , Beazer‐Barclay, Y. , Bryan, T. M. , Hamilton, S. R. , Thibodeau, S. N. , … Kinzler, K. W. (1992). APC mutations occur early during colorectal tumorigenesis. Nature, 359(6392), 235–237. 10.1038/359235a0 1528264

[wsbm1511-bib-0180] Prieve, M. G. , & Waterman, M. L. (1999). Nuclear localization and formation of β‐catenin–lymphoid enhancer factor 1 complexes are not sufficient for activation of gene expression. Molecular and Cellular Biology, 19(6), 4503–4515. 10.1128/mcb.19.6.4503 10330189 PMC104408

[wsbm1511-bib-0181] Proffitt, K. D. , Madan, B. , Ke, Z. , Pendharkar, V. , Ding, L. , Lee, M. A. , … Virshup, D. M. (2013). Pharmacological inhibition of the Wnt acyltransferase PORCN prevents growth of WNT‐driven mammary cancer. Cancer Research, 73(2), 502–507. 10.1158/0008-5472.CAN-12-2258 23188502

[wsbm1511-bib-0182] Ramain, P. , Khechumian, R. , Khechumian, K. , Arbogast, N. , Ackermann, C. , & Heitzler, P. (2000). Interactions between chip and the achaete/scute‐daughterless heterodimers are required for pannier‐driven proneural patterning. Molecular Cell, 6(4), 781–790. 10.1016/S1097-2765(05)00079-1 11090617

[wsbm1511-bib-0183] Reinhold, M. I. , & Naski, M. C. (2007). Direct interactions of Runx2 and canonical Wnt signaling induce FGF18. Journal of Biological Chemistry, 282(6), 3653–3663. 10.1074/jbc.M608995200 17158875

[wsbm1511-bib-0184] Reményi, A. , Schöler, H. R. , & Wilmanns, M. (2004). Combinatorial control of gene expression. Nature Structural and Molecular Biology, 11(9), 812–815. 10.1038/nsmb820 15332082

[wsbm1511-bib-0185] Remeseiro, S. , Hörnblad, A. , & Spitz, F. (2016). Gene regulation during development in the light of topologically associating domains. Wiley Interdisciplinary Reviews: Developmental Biology, 5(2), 169–185. 10.1002/wdev.218 26558551

[wsbm1511-bib-0186] Renko, M. , Fiedler, M. , Rutherford, T. J. , Schaefer, J. V. , Plückthun, A. , & Bienz, M. (2019). Rotational symmetry of the structured Chip/LDB‐SSDP core module of the Wnt enhanceosome. Proceedings of the National Academy of Sciences of the United States of America, 116(42), 20977–20983. 10.1073/pnas.1912705116 31570581 PMC6800368

[wsbm1511-bib-0187] Restrepo, S. , & Basler, K. (2011). Morphogen gradients: Expand and repress. Current Biology, 21(19), R815–R817. 10.1016/j.cub.2011.08.041 21996505

[wsbm1511-bib-0188] Riggleman, B. , Schedl, P. , & Wieschaus, E. (1990). Spatial expression of the Drosophila segment polarity gene armadillo is posttranscriptionally regulated by wingless. Cell, 63(3), 549–560. 10.1016/0092-8674(90)90451-J 2225066

[wsbm1511-bib-0189] Rijsewijk, F. , Schuermann, M. , Wagenaar, E. , Parren, P. , Weigel, D. , & Nusse, R. (1987). The Drosophila homology of the mouse mammary oncogene int‐1 is identical to the segment polarity gene wingless. Cell, 50(4), 649–657. 10.1016/0092-8674(87)90038-9 3111720

[wsbm1511-bib-0190] Rocha, P. P. , Scholze, M. , Bleiß, W. , & Schrewe, H. (2010). Med12 is essential for early mouse development and for canonical Wnt and Wnt/PCP signaling. Development, 137(16), 2723–2731. 10.1242/dev.053660 20630950

[wsbm1511-bib-0191] Roose, J. , Huls, G. , Van Beest, M. , Moerer, P. , Van Der Horn, K. , Goldschmeding, R. , … Clevers, H. (1999). Synergy between tumor suppressor APC and the β‐catenin‐Tcf4 target Tcf1. Science, 285(5435), 1923–1926. 10.1126/science.285.5435.1923 10489374

[wsbm1511-bib-0192] Rosenbluh, J. , Nijhawan, D. , Cox, A. G. , Li, X. , Neal, J. T. , Schafer, E. J. , … Hahn, W. C. (2012). β‐Catenin‐driven cancers require a YAP1 transcriptional complex for survival and tumorigenesis. Cell, 151(7), 1457–1473. 10.1016/j.cell.2012.11.026 23245941 PMC3530160

[wsbm1511-bib-0193] Rubinfeld, B. , Souza, B. , Albert, I. , Müller, O. , Chamberlain, S. H. , Masiarz, F. R. , … Polakis, P. (1993). Association of the APC gene product with β‐catenin. Science, 262(5140), 1731–1734. 10.1126/science.8259518 8259518

[wsbm1511-bib-0194] Sachinidis, A. , Fleischmann, B. K. , Kolossov, E. , Wartenberg, M. , Sauer, H. , & Hescheler, J. (2003). Cardiac specific differentiation of mouse embryonic stem cells. Cardiovascular Research, 58(2), 278–291. 12757863 10.1016/s0008-6363(03)00248-7

[wsbm1511-bib-0195] Sampietro, J. , Dahlberg, C. L. , Cho, U. S. , Hinds, T. R. , Kimelman, D. , & Xu, W. (2006). Crystal structure of a beta‐catenin/BCL9/Tcf4 complex. Molecular Cell, 24(2), 293–300. 10.1016/j.molcel.2006.09.001 17052462

[wsbm1511-bib-0196] Sansom, O. J. , Reed, K. R. , Van De Wetering, M. , Muncan, V. , Winton, D. J. , Clevers, H. , & Clarke, A. R. (2005). Cyclin D1 is not an immediate target of β‐catenin following Apc loss in the intestine. Journal of Biological Chemistry, 280(31), 28463–28467. 10.1074/jbc.M500191200 15946945

[wsbm1511-bib-0197] Santoro, I. M. , Yi, T. M. , & Walsh, K. (1991). Identification of single‐stranded‐DNA‐binding proteins that interact with muscle gene elements. Molecular and Cellular Biology, 11(4), 1944–1953. 10.1128/mcb.11.4.1944 2005890 PMC359879

[wsbm1511-bib-0198] Sato, T. , van Es, J. H. , Snippert, H. J. , Stange, D. E. , Vries, R. G. , van den Born, M. , … Clevers, H. (2011). Paneth cells constitute the niche for Lgr5 stem cells in intestinal crypts. Nature, 469(7330), 415–418. 10.1038/nature09637 21113151 PMC3547360

[wsbm1511-bib-0199] Saxena, M. , Kalathur, R. K. R. , Rubinstein, N. , Vettiger, A. , Sugiyama, N. , Neutzer, M. , … Christofori, G. (2020). A Pygopus 2‐histone interaction is critical for cancer cell de‐differentiation and progression in malignant breast cancer. Cancer Research, 1(603), 1–31. 10.1158/1078-0432.CCR-19-2706 32586983

[wsbm1511-bib-0200] Schuijers, J. , Junker, J. P. , Mokry, M. , Hatzis, P. , Koo, B. K. , Sasselli, V. , … Clevers, H. (2015). Ascl2 acts as an R‐spondin/wnt‐responsive switch to control stemness in intestinal crypts. Cell Stem Cell, 16(2), 158–170. 10.1016/j.stem.2014.12.006 25620640

[wsbm1511-bib-0201] Schuijers, J. , Mokry, M. , Hatzis, P. , Cuppen, E. , & Clevers, H. (2014). Wnt‐induced transcriptional activation is exclusively mediated by TCF/LEF. The EMBO Journal, 33(2), 146–156. 10.1002/embj.201385358 24413017 PMC3989608

[wsbm1511-bib-0202] Schwab, K. R. , Patterson, L. T. , Hartman, H. a. , Song, N. , Lang, R. a. , Lin, X. , & Potter, S. S. (2007). Pygo1 and Pygo2 roles in Wnt signaling in mammalian kidney development. BMC Biology, 5, 15. 10.1186/1741-7007-5-15 17425782 PMC1858683

[wsbm1511-bib-0203] Schwarz‐Romond, T. , Fiedler, M. , Shibata, N. , Butler, P. J. G. , Kikuchi, A. , Higuchi, Y. , & Bienz, M. (2007). The DIX domain of Dishevelled confers Wnt signaling by dynamic polymerization. Nature Structural and Molecular Biology, 14(6), 484–492. 10.1038/nsmb1247 17529994

[wsbm1511-bib-0204] Sekiya, T. , & Zaret, K. S. (2007). Repression by Groucho/TLE/Grg proteins: Genomic site recruitment generates compacted chromatin in vitro and impairs activator binding in vivo. Molecular Cell, 28(2), 291–303. 10.1016/j.molcel.2007.10.002 17964267 PMC2083644

[wsbm1511-bib-0205] Shtutman, M. , Zhurinsky, J. , Simcha, I. , Albanese, C. , D'Amico, M. , Pestell, R. , & Ben‐Ze'ev, A. (1999). The cyclin D1 gene is a target of the β‐catenin/LEF‐1 pathway. Proceedings of the National Academy of Sciences of the United States of America, 96(10), 5522–5527. 10.1073/pnas.96.10.5522 10318916 PMC21892

[wsbm1511-bib-0206] Sierra, J. , Yoshida, T. , Joazeiro, C. A. , & Jones, K. A. (2006). The APC tumor suppressor counteracts β‐catenin activation and H3K4 methylation at Wnt target genes. *Genes and* . Development, 20(5), 586–600. 10.1101/gad.1385806 PMC1410807 16510874

[wsbm1511-bib-0207] Sinner, D. , Rankin, S. , Lee, M. , & Zorn, A. M. (2004). Sox17 and beta‐catenin cooperate to regulate the transcription of endodermal genes. Development, 131(13), 3069–3080. 10.1242/dev.01176 15163629

[wsbm1511-bib-0208] Soutourina, J. (2018). Transcription regulation by the mediator complex. Nature Reviews Molecular Cell Biology, 19(4), 262–274. 10.1038/nrm.2017.115 29209056

[wsbm1511-bib-0209] Städeli, R. , & Basler, K. (2005). Dissecting nuclear wingless signalling: Recruitment of the transcriptional co‐activator Pygopus by a chain of adaptor proteins. Mechanisms of Development, 122(11), 1171–1182. 16169192 10.1016/j.mod.2005.07.004

[wsbm1511-bib-0210] Su, L. K. , Vogelstein, B. , & Kinzler, K. W. (1993). Association of the APC tumor suppressor protein with catenins. Science, 262(5140), 1734–1737. 10.1126/science.8259519 8259519

[wsbm1511-bib-0211] Sweeney, K. , Cameron, E. R. , & Blyth, K. (2020). Complex interplay between the RUNX transcription factors and Wnt/β‐catenin pathway in Cancer: A tango in the night. Molecules and Cells, 43(2), 188–197. 10.14348/molcells.2019.0310 32041394 PMC7057843

[wsbm1511-bib-0212] Tago, K. , Nakamura, T. , Nishita, M. , Hyodo, J. , Nagai, S. , Murata, Y. , … Akiyama, T. (2000). Inhibition of Wnt signaling by ICAT, a novel beta‐catenin‐interacting protein. Genes & Development, 14(14), 1741–1749. 10898789 PMC316784

[wsbm1511-bib-0213] Takada, K. , Zhu, D. , Bird, G. H. , Sukhdeo, K. , Zhao, J.‐J. , Mani, M. , … Carrasco, D. R. (2012). Targeted disruption of the BCL9/β‐catenin complex inhibits oncogenic Wnt signaling. Science Translational Medicine, 4(148), 148ra117. 10.1126/scitranslmed.3003808 PMC3631420 22914623

[wsbm1511-bib-0214] Takemaru, K. , Yamaguchi, S. , Lee, Y. S. , Zhang, Y. , Carthew, R. W. , & Moon, R. T. (2003). Chibby, a nuclear beta‐catenin‐associated antagonist of the Wnt/Wingless pathway. Nature, 422(6934), 905–909. 12712206 10.1038/nature01570

[wsbm1511-bib-0215] Takemaru, K.‐I. , & Moon, R. T. (2000). The transcriptional coactivator Cbp interacts with beta‐catenin to activate gene expression. The Journal of Cell Biology, 149(2), 249–254. 10.1083/jcb.149.2.249 10769018 PMC2175158

[wsbm1511-bib-0216] Tanaka, E. M. , & Reddien, P. W. (2011). The cellular basis for animal regeneration. Developmental Cell, 21(1), 172–185. 10.1016/j.devcel.2011.06.016 21763617 PMC3139400

[wsbm1511-bib-0217] Tang, N. , Song, W. X. , Luo, J. , Luo, X. , Chen, J. , Sharff, K. A. , … He, T. C. (2009). BMP‐9‐induced osteogenic differentiation of mesenchymal progenitors requires functional canonical Wnt/β‐catenin signalling. Journal of Cellular and Molecular Medicine, 13(8 B), 2448–2464. 10.1111/j.1582-4934.2008.00569.x 19175684 PMC4940786

[wsbm1511-bib-0218] Tetsu, O. , & McCormick, F. (1999). β‐Catenin regulates expression of cyclin D1 in colon carcinoma cells. Nature, 398(6726), 422–426. 10.1038/18884 10201372

[wsbm1511-bib-0219] Thompson, B. , Townsley, F. , Rosin‐Arbesfeld, R. , Musisi, H. , & Bienz, M. (2002). A new nuclear component of the Wnt signalling pathway. Nature Cell Biology, 4(5), 367–373. 10.1038/ncb786 11988739

[wsbm1511-bib-0220] Tortelote, G. G. , Reis, R. R. , de Almeida Mendes, F. , & Abreu, J. G. (2017). Complexity of the Wnt/β‐catenin pathway: Searching for an activation model. Cellular Signalling, 40(August), 30–43. 10.1016/j.cellsig.2017.08.008 28844868

[wsbm1511-bib-0221] Townsley, F. M. , Thompson, B. , & Bienz, M. (2004). Pygopus residues required for its binding to legless are critical for transcription and development. The Journal of Biological Chemistry, 279(7), 5177–5183. 14612447 10.1074/jbc.M309722200

[wsbm1511-bib-0222] Townsley, F. M. , Cliffe, A. , & Bienz, M. (2004). Pygopus and legless target Armadillo/beta‐catenin to the nucleus to enable its transcriptional co‐activator function. Nature Cell Biology, 6(7), 626–633. 10.1038/ncb1141 15208637

[wsbm1511-bib-0223] Tsai, MC. , Huang, CC. , Wei, YC. , Liu, TT. , Lin, MT. , Yi, LN. , … Tai, MH. (2020). Combined chibby and β‐catenin predicts clinical outcomes in patients with hepatocellular carcinoma. International Journal of Molecular Sciences, 21(6), 1–15. 10.3390/ijms21062060 PMC7139567 32192213

[wsbm1511-bib-0224] Vadlamudi, U. (2005). PITX2, beta‐catenin and LEF‐1 interact to synergistically regulate the LEF‐1 promoter. Journal of Cell Science, 118(6), 1129–1137. 10.1242/jcs.01706 15728254

[wsbm1511-bib-0225] Valenta, T. , Degirmenci, B. , Moor, A. E. , Herr, P. , Zimmerli, D. , Moor, M. B. , … Basler, K. (2016). Wnt ligands secreted by subepithelial Mesenchymal cells are essential for the survival of intestinal stem cells and gut homeostasis. Cell Reports, 15(5), 911–918. 10.1016/j.celrep.2016.03.088 27117411

[wsbm1511-bib-0226] Valenta, T. , Gay, M. , Steiner, S. , Draganova, K. , Zemke, M. , Hoffmans, R. , … Basler, K. (2011). Probing transcription‐specific outputs of β‐catenin in vivo. Genes & Development, 25(24), 2631–2643. 10.1101/gad.181289.111 22190459 PMC3248684

[wsbm1511-bib-0227] Valenta, T. , Hausmann, G. , & Basler, K. (2012). The many faces and functions of β‐catenin: β‐catenin: A life by, beyond, and against the Wnt canon. The EMBO Journal, 31(12), 2714–2736. 10.1038/emboj.2012.150 22617422 PMC3380220

[wsbm1511-bib-0228] van Amerongen, R. (2020). Celebrating discoveries in Wnt signaling: How one man gave wings to an entire field. Cell, 181, 1–5. 10.1016/j.cell.2020.03.033 32234518

[wsbm1511-bib-0229] Van Beest, M. , Dooijes, D. , Van De Wetering, M. , Kjaerulff, S. , Bonvin, A. , Nielsen, O. , & Clevers, H. (2000). Sequence‐specific high mobility group box factors recognize 10‐12‐base pair minor groove motifs. Journal of Biological Chemistry, 275(35), 27266–27273. 10.1074/jbc.M004102200 10867006

[wsbm1511-bib-0230] Van de Wetering, M. , Castrop, J. , Korinek, V. , & Clevers, H. (1996). Extensive alternative splicing and dual promoter usage generate Tcf‐1 protein isoforms with differential transcription control properties. Molecular and Cellular Biology, 16(3), 745–752. 10.1128/mcb.16.3.745 8622675 PMC231054

[wsbm1511-bib-0231] van de Wetering, M. , Cavallo, R. , Dooijes, D. , Van Beest, M. , Van Es, J. , Loureiro, J. , … Clevers, H. (1997). Armadillo coactivates transcription driven by the product of the Drosophila segment polarity gene dTCF. Cell, 88(6), 789–799. 10.1016/S0092-8674(00)81925-X 9118222

[wsbm1511-bib-0232] van de Wetering, M. , Sancho, E. , Verweij, C. , de Lau, W. , Oving, I. , Hurlstone, A. , … Clevers, H. (2002). The beta‐catenin/TCF‐4 complex imposes a crypt progenitor phenotype on colorectal cancer cells. Cell, 111(2), 241–250 http://www.ncbi.nlm.nih.gov/pubmed/12408868 12408868 10.1016/s0092-8674(02)01014-0

[wsbm1511-bib-0233] van Kappel, E. C. , & Maurice, M. M. (2017). Molecular regulation and pharmacological targeting of the β‐catenin destruction complex. British Journal of Pharmacology, 174(24), 4575–4588. 10.1111/bph.13922 28634996 PMC5727331

[wsbm1511-bib-0234] Van Meyel, D. J. , O'Keefe, D. D. , Jurata, L. W. , Thor, S. , Gill, G. N. , & Thomas, J. B. (1999). Chip and Apterous physically interact to form a functional complex during Drosophila development. Molecular Cell, 4(2), 259–265. 10.1016/S1097-2765(00)80373-1 10488341

[wsbm1511-bib-0235] van Meyel, D. J. , Thomas, J. B. , & Agulnick, A. D. (2003). Ssdp proteins bind to LIM‐interacting co‐factors and regulate the activity of LIM‐homeodomain protein complexes in vivo. Development, 130(9), 1915–1925. 10.1242/dev.00389 12642495

[wsbm1511-bib-0236] Van Oss, S. B. , Cucinotta, C. E. , & Arndt, K. M. (2017). Emerging insights into the roles of the Paf1 complex in gene regulation. Trends in Biochemical Sciences, 42(10), 788–798. 10.1016/j.tibs.2017.08.003 28870425 PMC5658044

[wsbm1511-bib-0237] van Tienen, L. M. , Mieszczanek, J. , Fiedler, M. , Rutherford, T. J. , Bienz, M. , Labhart, T. , … Park, P. (2017). Constitutive scaffolding of multiple Wnt enhanceosome components by legless/BCL9. eLife, 6, 477–488. 10.7554/eLife.20882 PMC5352222 28296634

[wsbm1511-bib-0238] Varnat, F. , Siegl‐Cachedenier, I. , Malerba, M. , Gervaz, P. , Ruiz, I. , & Altaba, A. (2010). Loss of WNT‐TCF addiction and enhancement of HH‐GLI1 signalling define the metastatic transition of human colon carcinomas. EMBO Molecular Medicine, 2(11), 440–457. 10.1002/emmm.201000098 20941789 PMC3394505

[wsbm1511-bib-0239] Velten, L. , Haas, S. F. , Raffel, S. , Blaszkiewicz, S. , Islam, S. , Hennig, B. P. , … Steinmetz, L. M. (2017). Human haematopoietic stem cell lineage commitment is a continuous process. Nature Cell Biology, 19(4), 271–281. 10.1038/ncb3493 28319093 PMC5496982

[wsbm1511-bib-0240] Verdone, L. , Agricola, E. , Caserta, M. , & Di Mauro, E. (2006). Histone acetylation in gene regulation. Briefings in Functional Genomics and Proteomics, 5(3), 209–221. 10.1093/bfgp/ell028 16877467

[wsbm1511-bib-0241] Vlaming, H. , Welsem, T. , Graaf, E. L. , Ontoso, D. , Altelaar, A. M. , San‐Segundo, P. A. , … Leeuwen, F. (2014). Flexibility in crosstalk between H2B ubiquitination and H3 methylation in vivo. EMBO Reports, 15(11), 1220–1221. 10.15252/embr.201471110 PMC4253848 25141862

[wsbm1511-bib-0242] Waltzer, L. , & Bienz, M. (1998). Drosophila CBP represses the transcription factor TCF to antagonize wingless signalling. Nature, 395, 521–525. 9774110 10.1038/26785

[wsbm1511-bib-0243] Waterman, M. L. , Fischer, W. H. , & Jones, K. A. (1991). A thymus‐specific member of the HMG protein family regulates the human T cell receptor Cα enhancer. Genes and Development, 5(4), 656–669. 10.1101/gad.5.4.656 2010090

[wsbm1511-bib-0244] Wehrli, M. , Dougan, S. T. , Caldwell, K. , O'Keefe, L. , Schwartz, S. , Valzel‐Ohayon, D. , … DiNardo, S. (2000). Arrow encodes an LDL‐receptor‐related protein essential for wingless signalling. Nature, 407(6803), 527–530. 10.1038/35035110 11029006

[wsbm1511-bib-0245] Whyte, J. L. , Smith, A. A. , & Helms, J. A. (2012). Wnt signaling and injury repair. Cold Spring Harbor Perspectives in Biology, 4(8), a008078. 10.1101/cshperspect.a008078 22723493 PMC3405869

[wsbm1511-bib-0246] Wiese, K. E. , Nusse, R. , & van Amerongen, R. (2018). Wnt signalling: Conquering complexity. Development (Cambridge), 145(12), 1–9. 10.1242/dev.165902 29945986

[wsbm1511-bib-0247] Willis, T. G. , Zalcberg, I. R. , Coignet, L. J. , Wlodarska, I. , Stul, M. , Jadayel, D. M. , … Dyer, M. J. (1998). Molecular cloning of translocation t(1;14)(q21;q32) defines a novel gene (BCL9) at chromosome 1q21. Blood, 91(6), 1873–1881 http://www.ncbi.nlm.nih.gov/pubmed/9490669 9490669

[wsbm1511-bib-0248] Wisniewski, J. A. , Yin, J. , Teuscher, K. B. , Zhang, M. , & Ji, H. (2016). Structure‐based design of 1,4‐Dibenzoylpiperazines as β‐catenin/B‐cell lymphoma 9 protein–protein interaction inhibitors. ACS Medicinal Chemistry Letters, 7, 508–513. 10.1021/acsmedchemlett.5b00284 27190602 PMC4867476

[wsbm1511-bib-0249] Wu, B. , Piloto, S. , Zeng, W. , Hoverter, N. P. , Schilling, T. F. , & Waterman, M. L. (2013). Ring finger protein 14 is a new regulator of TCF/β‐catenin‐mediated transcription and colon cancer cell survival. EMBO Reports, 14(4), 347–355. 10.1038/embor.2013.19 23449499 PMC3615654

[wsbm1511-bib-0250] Xi, M. , Chen, T. , Wu, C. , Gao, X. , Wu, Y. , Luo, X. , … Sun, H. (2019). CDK8 as a therapeutic target for cancers and recent developments in discovery of CDK8 inhibitors. European Journal of Medicinal Chemistry, 164, 77–91. 10.1016/j.ejmech.2018.11.076 30594029

[wsbm1511-bib-0251] Xu, W. , Wang, Z. , Zhang, W. , Qian, K. , Li, H. , Kong, D. , … Tang, Y. (2015). Mutated K‐ras activates CDK8 to stimulate the epithelial‐to‐mesenchymal transition in pancreatic cancer in part via the Wnt/β‐catenin signaling pathway. Cancer Letters, 356(2), 613–627. 10.1016/j.canlet.2014.10.008 25305448

[wsbm1511-bib-0252] Xu, W. , & Kimelman, D. (2007). Mechanistic insights from structural studies of β‐catenin and its binding partners. Journal of Cell Science, 120(19), 3337–3344. 10.1242/jcs.013771 17881495

[wsbm1511-bib-0253] Yamamoto, H. , Ihara, M. , Matsuura, Y. , & Kikuchi, A. (2003). Sumoylation is involved in β‐catenin‐dependent activation of Tcf‐4. EMBO Journal, 22(9), 2047–2059. 10.1093/emboj/cdg204 12727872 PMC156076

[wsbm1511-bib-0254] Yang, F. , Li, X. , Sharma, M. , Sasaki, C. Y. , Longo, D. L. , Lim, B. , & Sun, Z. (2002). Linking β‐catenin to androgen‐signaling pathway. Journal of Biological Chemistry, 277(13), 11336–11344. 10.1074/jbc.M111962200 11792709

[wsbm1511-bib-0255] Yi, F. , Pereira, L. , Hoffman, J. A. , Shy, B. R. , Yuen, C. M. , Liu, D. R. , & Merrill, B. J. (2011). Opposing effects of Tcf3 and Tcf1 control Wnt stimulation of embryonic stem cell self‐renewal. Nature Cell Biology, 13(7), 762–770. 10.1038/ncb2283 21685894 PMC3129424

[wsbm1511-bib-0256] Yochum, G. S. (2011). Multiple Wnt/ß‐catenin responsive enhancers align with the MYC promoter through long‐range chromatin loops. PLoS One, 6(4), e18966. 10.1371/journal.pone.0018966 21533051 PMC3080403

[wsbm1511-bib-0257] Yoda, A. , Kouike, H. , Okano, H. , & Sawa, H. (2005). Components of the transcriptional mediator complex are required for asymmetric cell division in *C.elegans* . Development, 132(8), 1885–1893. 10.1242/dev.01776 15790964

[wsbm1511-bib-0258] Yokoyama, N. N. , Pate, K. T. , Sprowl, S. , & Waterman, M. L. (2010). A role for YY1 in repression of dominant negative LEF‐1 expression in colon cancer. Nucleic Acids Research, 38(19), 6375–6388. 10.1093/nar/gkq492 20525792 PMC2965227

[wsbm1511-bib-0259] Yumoto, F. , Nguyen, P. , Sablin, E. P. , Baxter, J. D. , Webb, P. , & Fletterick, R. J. (2012). Structural basis of coactivation of liver receptor homolog‐1 by β‐catenin. Proceedings of the National Academy of Sciences of the United States of America, 109(1), 143–148. 10.1073/pnas.1117036108 22187462 PMC3252924

[wsbm1511-bib-0260] Zhang, X. , Peterson, K. A. , Liu, X. S. , Mcmahon, A. P. , & Ohba, S. (2013). Gene regulatory networks mediating canonical wnt signal‐directed control of pluripotency and differentiation in embryo stem cells. Stem Cells, 31(12), 2667–2679. 10.1002/stem.1371 23505158 PMC3830733

[wsbm1511-bib-0261] Zhou, H. , Kim, S. , Ishii, S. , & Boyer, T. G. (2006). Mediator modulates Gli3‐dependent sonic hedgehog signaling. Molecular and Cellular Biology, 26(23), 8667–8682. 10.1128/mcb.00443-06 17000779 PMC1636813

[wsbm1511-bib-0262] Zimmerli, D. , Borrelli, C. , Jauregi‐Miguel, A. , Söderholm, S. , Brütsch, S. , Doumpas, N. , … Cantù, C. (2020). TBX3 acts as tissue‐specific component of the Wnt/β‐catenin enhanceosome. eLife, 9, 1–17. 10.1101/2020.04.22.053561 PMC7434441 32808927

[wsbm1511-bib-0263] Zimmerli, D. , Hausmann, G. , Cantù, C. , & Basler, K. (2017). Pharmacological interventions in the Wnt pathway: Inhibition of Wnt secretion versus disrupting the protein–protein interfaces of nuclear factors. British Journal of Pharmacology, 174(24), 4600–4610. 10.1111/bph.13864 28521071 PMC5727313

[wsbm1511-bib-0264] Zorn, A. M. , Barish, G. D. , Williams, B. O. , Lavender, P. , Klymkowsky, M. W. , & Varmus, H. E. (1999). Regulation of Wnt signaling by sox proteins: XSox17α/β and XSox3 physically interact with β‐catenin. Molecular Cell, 4(4), 487–498. 10.1016/S1097-2765(00)80200-2 10549281

